# Battle of the Sex Chromosomes: Competition between X and Y Chromosome-Encoded Proteins for Partner Interaction and Chromatin Occupancy Drives Multicopy Gene Expression and Evolution in Muroid Rodents

**DOI:** 10.1093/molbev/msaa175

**Published:** 2020-07-13

**Authors:** Charlotte Moretti, Mélina Blanco, Côme Ialy-Radio, Maria-Elisabetta Serrentino, Clara Gobé, Robin Friedman, Christophe Battail, Marjorie Leduc, Monika A Ward, Daniel Vaiman, Frederic Tores, Julie Cocquet

**Affiliations:** 1 Institut Cochin, INSERM U1016, CNRS UMR8104, Université de Paris, Paris, France; 2 Ohana Biosciences, Cambridge, MA, USA; 3 Univ. Grenoble Alpes, CEA, INSERM, IRIG, Biology of Cancer and Infection UMR_S 1036, 38000 Grenoble, France; 4 Plateforme Protéomique 3P5, Institut Cochin, INSERM U1016, CNRS UMR8104, Université de Paris, Paris, France; 5 Institute for Biogenesis Research, John A. Burns School of Medicine, University of Hawaii, Honolulu, HI, USA; 6 Plateforme de Bio-informatique, Institut Imagine, Université de Paris, Paris, France; 7 Institut de Génomique Fonctionnelle de Lyon, Université de Lyon, CNRS UMR 5242, Ecole Normale Supérieure de Lyon, Université Claude Bernard Lyon 1, Lyon, France

**Keywords:** transmission distorters, sex ratio, sex chromosomes, spermatogenesis, rodent, gene regulation, multicopy genes, intragenomic conflict, H3K4 methylation

## Abstract

Transmission distorters (TDs) are genetic elements that favor their own transmission to the detriments of others. *Slx/Slxl1* (*Sycp3*-*like-X-linked* and *Slx-like1*) and *Sly* (*Sycp3-like-Y-linked*) are TDs, which have been coamplified on the X and Y chromosomes of *Mus* species. They are involved in an intragenomic conflict in which each favors its own transmission, resulting in sex ratio distortion of the progeny when *Slx/Slxl1* versus *Sly* copy number is unbalanced. They are specifically expressed in male postmeiotic gametes (spermatids) and have opposite effects on gene expression: *Sly* knockdown leads to the upregulation of hundreds of spermatid-expressed genes, whereas *Slx/Slxl1*-deficiency downregulates them. When both *Slx/Slxl1* and *Sly* are knocked down, sex ratio distortion and gene deregulation are corrected. *Slx/Slxl1* and *Sly* are, therefore, in competition but the molecular mechanism remains unknown. By comparing their chromatin-binding profiles and protein partners, we show that SLX/SLXL1 and SLY proteins compete for interaction with H3K4me3-reader SSTY1 (*Spermiogenesis-specific-transcript-on-the-Y1*) at the promoter of thousands of genes to drive their expression, and that the opposite effect they have on gene expression is mediated by different abilities to recruit SMRT/N-Cor transcriptional complex. Their target genes are predominantly spermatid-specific multicopy genes encoded by the sex chromosomes and the autosomal *Speer/Takusan*. Many of them have coamplified with not only *Slx/Slxl1/Sly* but also *Ssty* during muroid rodent evolution. Overall, we identify *Ssty* as a key element of the X versus Y intragenomic conflict, which may have influenced gene content and hybrid sterility beyond *Mus* lineage since *Ssty* amplification on the Y predated that of *Slx/Slxl1/Sly*.

## Introduction

Transmission distorters (TDs) are genetic elements that favor their own transmission and are passed on to the next generation with a frequency that departs from the classic Mendelian pattern of inheritance. The skewed transmission (also called meiotic drive) generally occurs during gametogenesis, as a consequence of meiosis and postmeiotic competition between haploid gametes. Transmission distortion is often at the cost of individual fitness and TDs are also named “selfish genes” as they often act by disabling/killing noncarrier gametes (for reviews, see Partridge and Hurst 1998; Helleu et al. 2015; Lindholm et al. 2016; Courret et al. 2019; Zanders and Unckless 2019). Occurrence of TDs on the sex chromosomes leads to a sex-ratio distortion in the progeny. Because the two sexes are usually produced in equal numbers (Fisher 1930; Hamilton 1967), the skewed sex ratio is generally corrected by evolution and selection of suppressor genes, located either on an autosome or on the other sex chromosome. This intragenomic conflict leads to a genetic arm race, in which TDs and drive suppressors compete to alter/restore Mendelian transmission. It can significantly modify gene content and genome structure, and consequently create hybrid incompatibility, lineage divergence and eventually speciation (for recent reviews, see Lindholm et al. 2016; Larson et al. 2018; O'Neill MJ and O'Neill RJ 2018; Courret et al. 2019; Zanders and Unckless 2019).

Intragenomic conflicts and the presence of TDs in the genome are usually unraveled during mating of different subspecies or in genetically modified (mutant) animals, that is, when the balance between TDs and suppressors is disrupted. One detectable molecular signature of past or ongoing intragenomic conflicts is the coamplification of TDs and suppressors, which translates into striking copy number variants between subspecies (O'Neill MJ and O'Neill RJ 2018; Ellison and Bachtrog 2019).

*Slx/Slxl1* (*Sycp3 like X-linked* and *Slx-like 1*) and *Sly* (*Sycp3-like Y-linked*) multicopy genes are a paradigm of an intragenomic conflict between the sex chromosomes in the heterogametic sex. *Sly* is a mouse Y chromosome-encoded multicopy gene and *Slx/Slxl1* are its X-encoded homologs, encoded by two distinct ampliconic gene clusters, *Slx* and *Slxl1*. *Sly* and *Slx/Slxl1* are related to the autosomal gene *Sycp3* (*Synaptonemal complex protein 3*), which encodes an essential component of the synaptonemal complex during meiosis. The sequence similarity between SLY/SLX/SLXL1 and SYCP3 proteins is lower than among SLY/SLX/SLXL1 proteins (∼29% identities between SLY and SYCP3, ∼18% between SLX/SLXL1 and SYCP3, and ∼40% between SLX/SLXL1 and SLY). *Sly* and *Slx/Slxl1* are specific to the mouse lineage. It is presumed that *Sycp3* was initially duplicated on the rodent X chromosome, followed by neofunctionalization of the X chromosome-encoded copy, and emergence of a copy on the Y chromosome. Both X and Y versions were rapidly and massively coamplified during *Mus* evolution reaching hundreds of copies in some subgroups such as *Mus musculus* (Ellis et al. 2007, 2011; Scavetta and Tautz 2010; Morgan and Pardo-Manuel de Villena 2017; Kruger et al. 2019).

Their contribution to an ongoing intragenomic conflict between the mouse sex chromosomes has been unraveled by functional studies of *Slx/Slxl1* knocked-down (*Slx/Slxl1-*KD) and *Sly* knocked-down (*Sly*-KD) mice: *Sly* knockdown leads to sperm differentiation defects and near sterility with a higher production of female offspring for *Sly*-KD males able to sire progeny (Cocquet et al. 2009), and most of these defects are corrected when *Slx/Slxl1* are also knocked down in these mice (Cocquet et al. 2012). Conversely, *Slx*/*Slxl1* deficiency alone results in abnormal sperm differentiation and a skewed sex ratio toward the production of male progeny that are corrected by addition of a *Sly* knockdown (Cocquet et al. 2010, 2012). Male mice with duplication of *Slx* and *Slxl1* genes have been shown to produce a higher number of female progeny (Kruger et al. 2019). All these data show that a balanced copy number between *Sly* and *Slx/Slxl1* is necessary for normal sperm differentiation and equal transmission of X- and Y-bearing gametes to the progeny.

Interestingly, in naturally occurring mouse hybrid zones or in cases of interspecific crosses, impaired spermatogenesis and sex ratio distortion have been reported (Macholan et al. 2008; Good et al. 2010; Turner et al. 2012); unbalanced *Slx/Slxl1* versus *Sly* copy number could contribute to these phenotypes.

*Slx/Slxl1* and *Sly* genes are only expressed in postmeiotic (haploid) spermatids but because these cells develop in a pseudosyncytium (and therefore share most of their cytoplasmic content), both SLX/SLXL1 and SLY proteins are detected in X- and Y-bearing spermatids (Reynard et al. 2007, 2009; Cocquet et al. 2012). We have shown that SLY regulates gene expression predominantly via limiting expression of X and Y chromosome-encoded genes, including *Slx/Slxl1*, in spermatids (Cocquet et al. 2009). It has also been found to directly activate or repress autosomal spermatid-expressed genes (Moretti et al. 2017). Conversely, deletion of *Slx/Slxl1* genes leads to deregulation of nearly a thousand genes, in particular the downregulation of ∼100 X-linked genes and of the Y-encoded multicopy genes *Sly* and *Ssty* (*Spermiogenesis-specific transcript on the Y*) (Kruger et al. 2019). As a result, *Slx/Slxl1* deficiency corrects the XY gene upregulation induced by *Sly* knockdown showing that *Slx/Slxl1* and *Sly* have antagonistic effects on XY gene expression. Collectively, these functional studies indicate a competition between *Slx/Slxl1* and *Sly* gene products that warrants further characterization at the molecular level.

In the present study, we investigated the molecular roles of SLX/SLXL1 and SLY proteins by comparing their genomic locations, target genes, and protein partners. Based on our results, we propose a model that shows that SLX/SLXL1 and SLY compete for partner interaction with SSTY at the promoter of thousands of genes, in particular of spermatid-specific multicopy genes which have been coamplified with *Slx/Slxl1*, *Sly*, and *Ssty* during muroid rodent evolution.

## Results

### SLX/SLXL1 Genomic Targets Significantly Overlap with Active Epigenetic Marks and the Transcription Start Sites of SLY-Enriched Genes

**Fig. 1.**
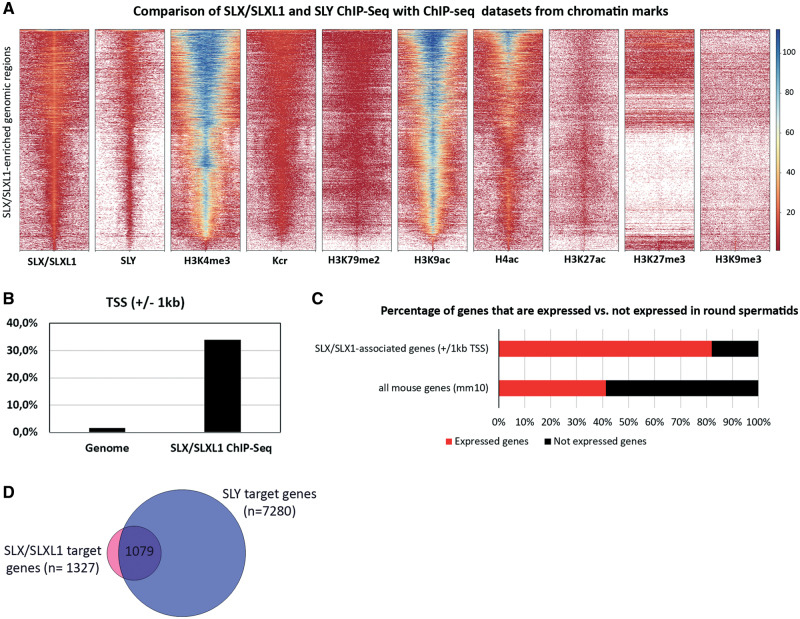
SLX/SLXL1 proteins are enriched at the TSS of the same spermatid-expressed genes as SLY, and correlate with active epigenetic marks, similarly to SLY. (*A*) Heatmap comparison of SLX/SLXL1 ChIP-Seq with SLY, Kcr, H3K4me3, H3K79me2, H3K9ac, H4ac, H3K27ac, H3K27me3, and H3K9me3 ChIP-Seq data sets, all performed in WT round spermatids. Each heatmap shows the scores (*k*-means) associated with SLX/SLXL1-enriched genomic regions (5 kb downstream to 5 kb upstream the center of the interval). The color code associated with enrichment values is indicated on the right. (*B*) Graphic representation of SLX/SLXL1 enrichment at the TSS of genes (±1 kb) (χ^2^, *P* < 0.0001). (*C*) Percentage of genes that are expressed (red) and not expressed (black) in round spermatids, among all mouse genes (mm10 genome version) or among SLX/SLXL1-associated genes. SLX/SLXL1 is significantly enriched at the start (i.e., TSS ± 1 kb) of genes expressed in round spermatids (χ^2^, *P* < 0.0001). (*D*) Venn diagram representing the number of common target genes between SLX/SLXL1 and SLY in WT round spermatids (same conditions as SLY-ChIP-Seq from Moretti et al. [2017]).

SLY has previously been shown to bind to the transcription start sites (TSS) of spermatid-expressed genes and to colocalize with chromatin marks associated with the promoter of expressed genes, such as H3K4me3 (trimethylation of histone H3 lysine 4), Kcr (histone lysine crotonylation), H3K9ac (acetylation of histone H3 lysine 9), H3K27ac (acetylation of histone H3 lysine 27), and H4ac (acetylation of histone H4) (Moretti et al. 2017). To start investigating the molecular role of SLX/SLXL1, we performed SLX/SLXL1 chromatin immunoprecipitation followed by high-throughput sequencing (ChIP-Seq) analysis on purified round spermatids from wild-type (WT) males, using the same material and conditions as used before to identify SLY genomic targets (Moretti et al. 2017) (supplementary table S1, Supplementary Material online). First, we compared SLX/SLXL1 ChIP-Seq profile with that of SLY ChIP-Seq and of eight different histone marks obtained in WT round spermatids. We observed that most of SLX/SLXL1 genomic targets are among SLY genomic targets and overlap with active chromatin marks, such as Kcr, H3K4me3, H3K9ac, and histone lysine 79 dimethylation (H3K79me2) (fig. 1*A* and supplementary fig. S1, Supplementary Material online).

When annotating SLX/SLXL1 genomic targets, we observed a significant enrichment of SLX/SLXL1 at the start of genes (i.e., ∼34% overlap with ±1 kb of TSS, χ^2^, *P* < 0.0001) (fig. 1*B*). Remarkably, most of these genes are spermatid-expressed genes (i.e., ∼82% vs. ∼41%, χ^2^, *P* < 0.0001) (fig. 1*C*). Comparison with SLY target genes showed that most of SLX/SLXL1 target genes are also SLY target genes (∼81%) but representing only a limited number of SLY total targets (∼15%) (fig. 1*D*).

### *Sly* Knockdown Leads to an Increase in SLX/SLXL1 Genomic Distribution, in Particular at the TSS of Multicopy Genes of Chromosomes X, Y, 5 and 14

When *Sly* is knocked down, *Slx/Slxl1* transcription has been shown to be upregulated and the nuclear fraction of SLX/SLXL1 protein is highly increased (Cocquet et al. 2009, 2012). To determine the consequences of *Sly* knockdown on SLX/SLXL1 genomic distribution we performed, in parallel, SLX/SLXL1 ChIP-Seq in WT and *Sly*-KD spermatids in three independent experiments (see supplementary table S2, Supplementary Material online).

**Fig. 2.**
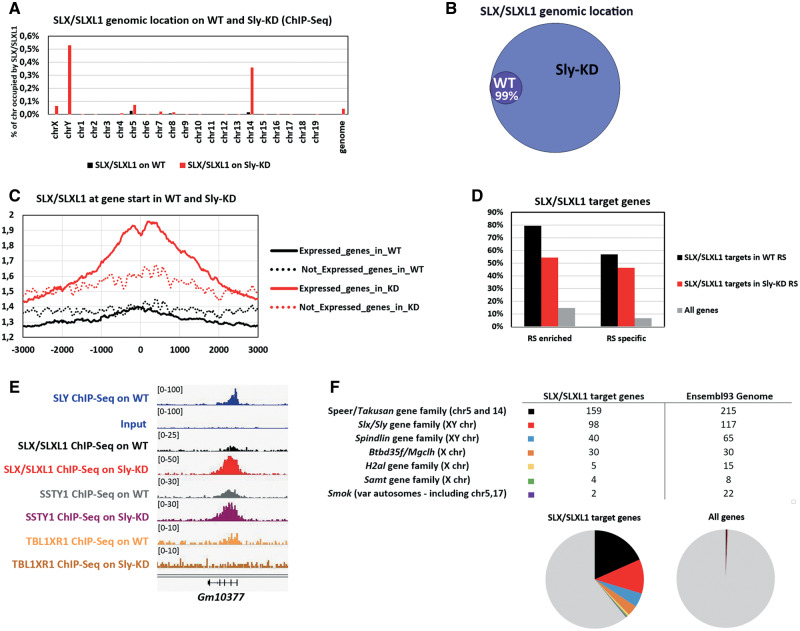
*Sly* knockdown leads to an increase in SLX/SLXL1 at the TSS of multicopy spermatid-expressed genes. Unless stated otherwise, the data presented in this figure represent the intersection of three independent experiments (i.e., regions found in common in the replicates); see supplementary figures S2 and S3, Supplementary Material online, for individual data. (*A*) SLX/SLXL1 genomic location (% of coverage of each chromosome) as identified by ChIP-Seq on WT and *Sly*-KD round spermatids. (*B*) Venn diagram showing the overlap between SLX/SLXL1 genomic targets in WT and *Sly*-KD round spermatids. (*C*) Graphic representation of SLX/SLXL1 ChIP-Seq profile in WT (black lines) and *Sly*-KD (red lines) round spermatids showing the average enrichment of SLX/SLXL1 around the TSS of genes expressed (unbroken lines) and not expressed (dotted lines) in round spermatids. (*D*) Percentage of SLX/SLXL1 target genes (TSS ± 1 kb) in WT round spermatids (black) and in *Sly*-KD round spermatids (red) that are enriched in round spermatids (upregulated >2× compared with pachytene spermatocytes) or specifically/de novo expressed in round spermatids (RS specific), compared with all mm10 genes (χ^2^, SLX/SLXL1 ChIP-Seq on WT, RS-enriched *P* < 0.0001; SLX/SLXL1 ChIP-Seq on WT, RS-specific *P* < 0.0001; SLX/SLXL1 ChIP-Seq on Sly-KD, RS-enriched *P* < 0.0001; SLX/SLXL1 ChIP-Seq on WT, RS-enriched *P* < 0.0001). (*E*) IGV screenshot showing SLY ChIP profile in WT round spermatids compared with its input (from Moretti et al. [2017]), SLX/SLXL1 (Exp1), SSTY1 (Exp1), and TBL1XR1 ChIP profile in WT and *Sly-*KD round spermatids. The genomic region shown is a portion of chromosome 14 encompassing *Gm10377*, a gene of *Takusan* family. (*F*) Number of SLX/SLXL1 target genes (in *Sly-*KD spermatids) belonging to multicopy gene families (gene copy number found using Ensembl Protein Domain ID). The pie charts underneath show the proportion of each indicated multicopy gene family compared with that of other genes (in gray) among SLX/SLXL1 target genes (left) and among all Ensembl93 mm10 genes (right).

Despite differences in the number of obtained peaks (probably due to modifications in the experimental procedure, see supplementary table S2, Supplementary Material online), overlaps between the three sets of experiments were high (supplementary fig. S2*A*, Supplementary Material online) and each pairwise comparison of SLX/SLXL1 ChIP-Seq between WT and *Sly*-KD round spermatids gave similar results: SLX/SLXL1 coverage was found broader in *Sly*-KD spermatids compared with WT spermatids (∼15-fold increase in coverage in *Sly*-KD vs. WT spermatids in Exp1, ∼17-fold increase in Exp2, and ∼6-fold increase in Exp3) with ∼99% of SLX/SLXL1 genomic location in WT spermatids maintained in *Sly*-KD spermatids when only considering regions in common in all WT replicates or in all *Sly*-KD replicates (fig. 2*A* and *B*; supplementary fig. S2*B* and *C*, Supplementary Material online). When focusing on annotated regions, the number of SLX/SLXL1 target genes increases dramatically in *Sly-*KD spermatids compared with WT spermatids (∼11-fold increase in the overlap of three experiments; ∼8-fold increase in Exp1, ∼15-fold increase in Exp2, and ∼6-fold increase in Exp3) (supplementary figs. S2*D*, S3*A*, and S3*B*, Supplementary Material online).

In both WT and *Sly-*KD spermatids, SLX/SLXL1 is enriched at the start of genes that are expressed in spermatids (fig. 2*C* and supplementary fig. S3*A*, Supplementary Material online) and, more specifically, upregulated in spermatids or specifically expressed in spermatids (fig. 2*D*). Besides, SLX/SLXL1 enrichment at those genes is higher when *Sly* is knocked down (fig. 2*C* and *E*). Strikingly, SLX/SLXL1 target genes are predominantly located on chromosomes 5, 14, X, and Y (fig. 2*A* and supplementary fig. S3*B*, Supplementary Material online); and, many of them are, in fact, multicopy genes (often ampliconic), amplified up to hundreds of copies in a particular chromosomal region. Those multicopy genes/gene families include *Slx/Sly* and *Spindlin* gene families, which are located on chromosomes X and Y, and several X-encoded gene families such as *H2al*, *Samt*, and *Mgclh* (aka *Btbd35f* or *Gmcl1l*) gene families. The most represented gene family is *Speer*/*Takusan* gene family, with an estimated 215 copies on *M. musculus* chromosomes 5 and 14 (Ensembl93), of which 159 are found among SLX/SLXL1 target genes when *Sly* is knocked down (fig. 2*F*). Other multicopy gene families were found on various autosomes, such as *Smok* gene family (i.e., 22 members found in Ensembl93, including *Smok2a*, *Smok2b*, *Smok3a*, *Smok3b*, and *Smok3c*; fig. 2*F*, see also supplementary fig. S6*D*, Supplementary Material online). All in all, we found that those multicopy gene families represent ∼39% of SLX/SLXL1 target genes in *Sly*-KD round spermatids (vs. 1% of all Ensembl93 genes, χ^2^, *P* < 0.0001). All of them are spermatid specific or highly upregulated at the spermatid stage (supplementary fig. S4, Supplementary Material online).

**Fig. 3.**
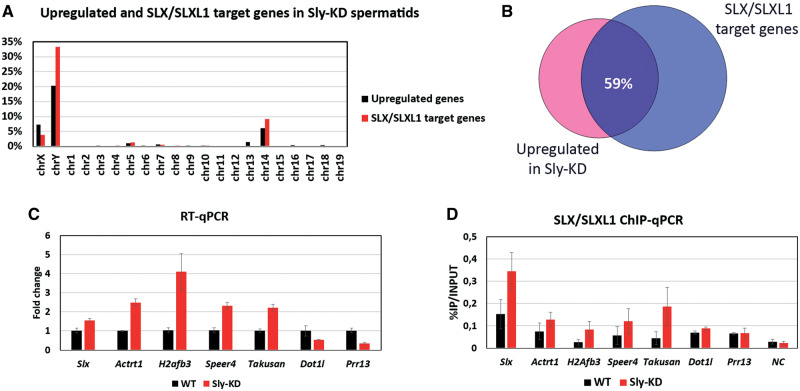
Genes enriched in SLX/SLXL1 are upregulated in *Sly-*KD round spermatids. (*A*) In black: location of upregulated genes identified *Sly*-KD compared with WT spermatids (>1.5×, *P* < 0.05 from Cocquet et al. [2009]; revisited in Moretti et al. [2017]). In red: location of SLX/SLXL1 target genes identified by ChIP-Seq (in common in the three SLX/SLXL1 ChIP-Seq Exp on *Sly*-KD round spermatids). (*B*) Venn diagram representing the percentage of genes found upregulated in *Sly*-KD spermatids that are among SLX/SLXL1 target genes (in common in the three SLX/SLXL1 ChIP-Seq Exp on *Sly*-KD round spermatids). (*C*, *D*) Examples of genes that are enriched in SLX/SLXL1 and are upregulated in *Sly*-KD round spermatids. (*C*) RT-qPCR quantification performed on WT and *Sly*-KD round spermatids. The graph represents the mean ± SEM (after normalization with β-actin, *n* = 3–4 samples per genotype). For all, a significant difference between *Sly-*KD and WT samples was found (*t*-test, *P*-value < 0.05). RT-qPCR data for *Actrt1* are from Cocquet et al. (2012); RT-qPCR data for *H2afb3* and *Dot1l* are from Moretti et al. (2017). (*D*) SLX/SLXL1 ChIP-qPCR performed on WT and *Sly*-KD round spermatids. The *Y*-axis represents the mean enrichment (% IP/input) ± SEM (*n* = 3–4 samples per genotype).

Interestingly, comparison of SLX/SLXL1 target genes in *Sly*-KD round spermatids (genes in common in the three ChIP-Seq replicates) with the genes found upregulated in *Sly*-KD round spermatids (Moretti et al. 2017) showed a significant overlap between the two data sets (i.e., ∼59% of genes in common, χ^2^, *P* < 0.0001) (fig. 3*A* and *B*). By reverse transcription followed by quantitative polymerase chain reaction (RT-qPCR) and ChIP-qPCR, we confirmed that many of SLX/SLXL1 target genes, whether single copy (such as the X-encoded genes *H2afb3* and *Actrt1*) or multicopy spermatid-expressed genes (such as *Slx* itself and *Speer/Takusan*), are found upregulated in *Sly*-KD round spermatids (fig. 3*C* and *D*) indicating a direct competition between SLX/SLXL1 and SLY proteins at the start of those genes to regulate their expression. Genes found downregulated in *Sly*-KD round spermatids, such as *Prr13* and *Dot1l*, or an intragenic negative control region (NC) are not enriched in SLX/SLXL1.

### SLX/SLXL1 and SLY Can Both Interact with the H3K4me3 Readers SSTY/SPIN but Are Not in the Same Complex

To unravel the mechanisms driving SLY and SLX/SLXL1 genomic binding competition, we sought for SLY and SLX/SLXL1 protein partners. We have previously shown that SLY and SLX/SLXL1 interact with SSTY1 and SSTY2 proteins (Comptour et al. 2014). By label-free histone peptide pull-down followed by mass spectrometry, Eberl et al. (2013) had also found that SSTY1/2, SLX/SLXL1, and SLY are part of a protein complex that interacts with H3K4me3 in mouse testes. SSTY1/2 proteins are related to SPIN1, a known H3K4me3 reader (Yang et al. 2012) which was also found to interact with H3K4me3 in mouse testes in the same study (Eberl et al. 2013). SPIN1 contains three structurally conserved domains (i.e., Spin/Tudor-like domains) (Wang et al. 2011) and the amino acid residues described to be necessary for H3K4me3 recognition are conserved between SPIN1, SSTY1, and SSTY2 (supplementary fig. S5*A*, Supplementary Material online). Collectively these data suggest that SSTY proteins interact with H3K4me3 via their Tudor-like domains. By immunoprecipitation, we observed that both SLX/SLXL1 and SLY are also able to interact with SPIN1 in the testes (supplementary fig. S5*B*, Supplementary Material online—see also fig. 5*B*); yet, we did not find SLX/SLXL1 and SLY in the same protein complex suggesting that they individually interact with SSTY/SPIN1 proteins (supplementary fig. S5*C*, Supplementary Material online), in agreement with recent findings from Kruger et al. (2019).

### SSTY1, SLX/SLXL1, and SLY Are Enriched at the TSS of the Same Spermatid-Expressed Genes

SSTY1 is a spermatid-specific nuclear protein that is upregulated in absence of SLY (Reynard et al. 2009) and has been shown to colocalize with the sex chromosomes in spermatids (Comptour et al. 2014). We therefore suspected that SSTY1 may be involved in SLY/SLX/SLXL1 regulation of sex chromosome gene expression.

**Fig. 4.**
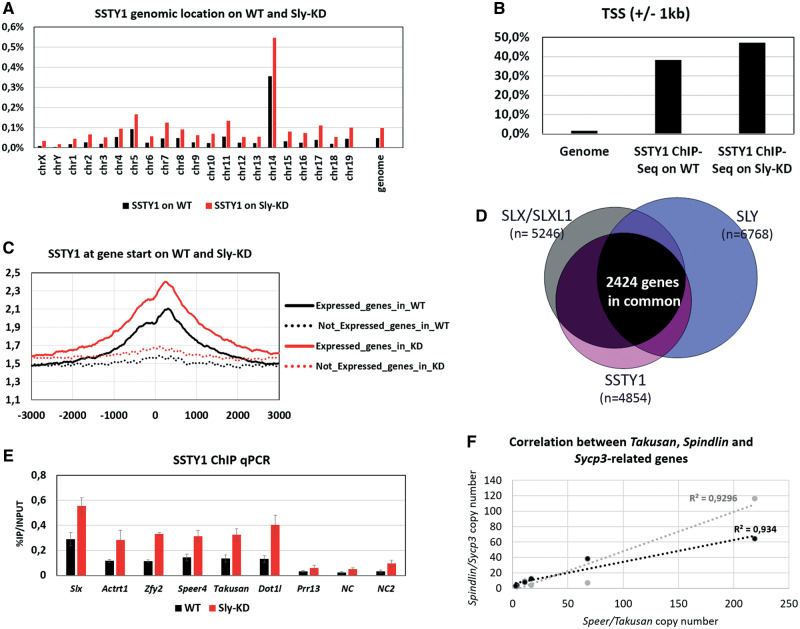
SSTY1 colocalizes with SLX/SLXL1 and SLY at the TSS of spermatid-expressed genes. Unless stated otherwise, the data presented in this figure represent the intersection of two independent experiments (i.e., regions found in common in the replicates); see supplementary figure S6, Supplementary Material online, for individual data. (*A*) SSTY1 genomic location (% of coverage of each chromosome) as identified by ChIP-Seq on WT (black) and *Sly*-KD (red) round spermatids. (*B*) Graphic representation of SSTY1 enrichment at the TSS of genes (±1 kb) in WT and *Sly*-KD spermatids (χ^2^, *P* < 0.0001 for both). (*C*) Graphic representation of SSTY ChIP-Seq profile in WT (black lines) and *Sly-*KD (red lines) round spermatids showing the average enrichment around the TSS of genes expressed (unbroken lines) and not expressed (dotted lines) in round spermatids. (*D*) Venn diagrams representing the number of common target genes between SLY (all target genes in WT round spermatids), SSTY1, and SLX/SLXL1 (all target genes in WT and *Sly*-KD round spermatids). The number of target genes presented for SLY is slightly different than in figure 1*D* (i.e., 6,768 genes instead of 7,280) because, for the sake of appropriate comparison between data sets, the bioinformatics pipeline chosen was the one described in supplementary tables S2 and S3, Supplementary Material online (and not supplementary table S1, Supplementary Material online). (E) SSTY1 ChIP-qPCR performed on WT and *Sly*-KD round spermatids. The *Y*-axis represents the mean enrichment (% IP/input) ± SEM (*n* = 3–4 samples per genotype). A significant difference (*t*-test, *P* < 0.05) was found for all regions shown on the graph except *Prr13* and *Actrt1*. (*F*) Scatter plot showing numbers of coding *Speer/Takusan* genes corresponding to *Sycp3*- or *Spindlin*-related genes in rodents (respectively, gray and black dots) from supplementary table S5, Supplementary Material online. *R*^2^ indicates the coefficient of each regression line.

To test for SSTY1 involvement, we investigated its genomic localization by ChIP-Seq in WT and *Sly*-KD round spermatids (fig. 4*A*, supplementary fig. S6*A*–*C* and table S3, Supplementary Material online). Annotation of its genomic targets showed a significant enrichment of SSTY1 at the start of genes (i.e., 38% and 47% overlap with ±1 kb of TSS in WT and *Sly*-KD spermatids; fig. 4*B* and supplementary fig. S6*D*–*F*, Supplementary Material online), in particular of spermatid-expressed genes (fig. 4*C*), similarly to SLX/SLXL1 and SLY (supplementary fig. S6*F* and *G*, Supplementary Material online). In fact, comparison of target genes confirmed that SSTY1 protein shares many target genes with SLY and SLX/SLXL1 (fig. 4*D* and supplementary fig. S6*H*, Supplementary Material online). This is particularly striking in *Sly*-KD spermatids where SSTY1 is upregulated along with SLX/SLXL1, and both are enriched at the same target genes (81% of targets in common), in particular spermatid-specific multicopy genes of chromosome 5, 14, X, and Y (figs. 2*E* and 4*E*, supplementary fig. S6*I*, Supplementary Material online). Consistent with its upregulation, SSTY1 enrichment at gene start is significantly higher in *Sly*-KD than in WT spermatids (figs. 2*E* and 4*C*, supplementary fig. S6*I*, Supplementary Material online). These observations were confirmed by ChIP-qPCR in which all tested SSTY target genes (The X chromosome-encoded genes *Slx* and *Actrt1*, the Y chromosome-encoded gene *Zfy2*, and autosomal genes *Speer4*, *Takusan* and *Dot1l*) showed a stronger enrichment in *Sly*-KD than in WT spermatids (fig. 4*E*). *Speer*/*Takusan* gene family appears to be a preferential target of SLY, SLX/SLXL1, and SSTY: 104 *Speer/Takusan* genes are among the 10% of genes with highest SLY peak (Moretti et al. 2017), and 175 are among all SLY, SLX/SLXL1, and SSTY1 common targets (in bold, supplementary table S4, Supplementary Material online).

### *Slxl1*, *Sly*, *Ssty*, and Their Target Genes Were Coamplified during Rodent Evolution

Intrigued by the high copy number of *Speer/Takusan* genes in *M. musculus* genome, we next searched for the presence of orthologs in other species, using Takusan Ensembl protein domain (i.e., PTHR21558). We found genes coding for proteins of the same family only in six other muroid rodent species (supplementary table S5, Supplementary Material online). *Speer/Takusan* originated from a copy of *Dlg5* (discs large MAGUK scaffold protein 5) but have diverged and form a distinct gene family. On the contrary to *Speer/Takusan* genes, *Dlg5* is a single-copy gene, present in many species. Interestingly, the rat, in which an ancestral copy of *Slxl1* (but not of *Sly*) was identified (Kruger et al. 2019), appears to have a high gene copy number of *Speer/Takusan* as well as of *Ssty.* We next compared coding gene copy numbers in the seven muroid rodent species (including *M. musculus*) listed in supplementary table S5, Supplementary Material online, and found that *Speer/Takusan* copy number showed a strong correlation with that of *Sycp3*-related genes (*R*^2^ = 0.9293, *P* = 0.0005) and *Spindlin*-related genes (*R*^2^ = 0.934, *P* = 0.0004) (fig. 4*F*) suggesting coamplification of *Slx/Sly*, *Ssty*, and *Takusan*.

It is worth mentioning that several sex chromosome-encoded multicopy genes are found among SLX/SLXL1, SLY, and SSTY common targets, such as *Mgclh* or *H2al* multicopy genes. Their copy number also shows a strong positive correlation with that of *Sycp3*-related genes (using the seven rodent species listed in supplementary table S5, Supplementary Material online, respectively, *R*^2^ = 0.9971, *P* < 0.0001 for *Mgclh*; *R*^2^ = 09572, *P* = 0.0001 for *H2al*) in line with a study which reported that these sex chromosome-encoded genes were amplified along with *Sly* (Ellis et al. 2011). Their correlation with that of *Spindlin*-related coding gene number is also positive but less strong (respectively, *R*^2^ = 0.7406, *P* = 0.0129 for *Mgclh*; *R*^2^ = 0.6162 *P* = 0.0365 for *H2al*).

### SLY but Not SLX/SLXL1 Interacts with SMRT/N-Cor Repressive Complex and Affects Its Genomic Location

**Fig. 5.**
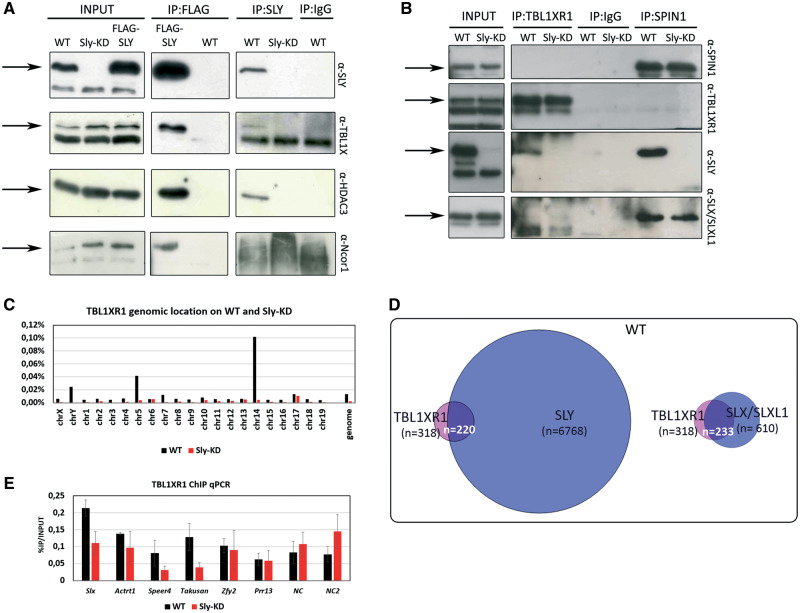
SMRT/N-Cor proteins interact preferentially with SLY and are enriched at TSS of the same genes. (*A*) Western blot detection with SLY, TBL1X, HDAC3, or N-COR1 antibody following immunoprecipitation (IP) with SLY or FLAG antibody on WT, FLAG-SLY, or *Sly*-KD testis. Immunoprecipitation with IgG was used as negative control. Input samples represent protein extracts before immunoprecipitation. Arrows indicate bands at the right size. (*B*) Western blot detection with SPIN1, TBL1XR1, SLY, or SLX/SLXL1 antibody following immunoprecipitation (IP) with TBL1XR1 or SPIN1 antibody on WT or *Sly*-KD testis. Immunoprecipitation with IgG was used as negative control. Input samples represent protein extracts before immunoprecipitation. Arrows indicate bands at the right size. (*C*) TBL1XR1 genomic location (% of coverage of each chromosome) as identified by ChIP-Seq on WT and *Sly*-KD round spermatids. (*D*) Venn diagrams representing the number of common target genes between TBL1XR1, SLX/SLXL1 (Exp1 see supplementary table S2, Supplementary Material online), and SLY (from Moretti et al. [2017]) in WT round spermatids. (*E*) TBL1XR1 ChIP-qPCR performed on WT and *Sly-*KD round spermatids. The *Y*-axis represents the mean enrichment (% IP/input) ± SEM (*n* = 3–4 samples per genotype). A significant difference (*t*-test, *P* < 0.05) was found for *Slx, Speer4*, and *Takusan* genes.

Our data showed that SSTY1 is a key component of the intragenomic conflict between SLY and SLX/SLXL1. We next searched for additional protein partners involved in gene regulation. We previously found that SLY interacts with TBL1XR1 (*Transducin Beta Like 1 X-Linked Receptor 1*), a member of the repressor complex SMRT/N-Cor (*Silencing Mediator of Retinoic acid and Thyroid hormone receptor/Nuclear Receptor Corepressor complex*) (Moretti et al. 2017). We therefore sought here to characterize this protein complex and test whether SLX/SLXL1 is involved. By immunoprecipitation with SLY antibody on WT testes and with FLAG antibody on FLAG–SLY transgenic testes, we first confirmed that SLY is in a protein complex with other members of SMRT/N-Cor, that is, TBL1X (*Transducin Beta Like 1 X-Linked*), HDAC3 (*Histone Deacetylase 3*), and NCor1 (*Nuclear Receptor Corepressor 1*) (fig. 5*A*). When we looked at SLX/SLXL1 and SPIN1, however, they were not pulled down by antibodies against TBL1XR1 (fig. 5*B*) nor TBL1X (data not shown), suggesting that only SLY interacts with the SMRT/N-Cor complex. We next investigated the pattern of expression and genomic location of SMRT/N-Cor proteins. Both TBL1X and TBL1XR1 are ubiquitously expressed (supplementary fig. S7*A*, Supplementary Material online) and, in male germ cells, they are expressed throughout spermatogenesis (supplementary fig. S7*B* and *C*, Supplementary Material online). Overall, TBL1XR1 level is higher than TLB1X, particularly in spermatogonia and Sertoli cells (supplementary fig. S7*B* and *C*, Supplementary Material online). No major differences in their pattern of expression or level were observed between WT and *Sly*-KD samples (supplementary fig. S7*C* and *D*, Supplementary Material online). By ChIP performed on round spermatids, we found that, in WT, TBL1XR1 is preferentially found on chromosomes 5, 14, and Y (fig. 5*C*). Overall, the number of TBL1XR1-enriched regions found was low (maybe due to the fact that SMRT/N-Cor level of expression is not high in spermatids) and only a few hundreds of target genes (±1 kb of TSS) were identified (supplementary table S6, Supplementary Material online). Yet, the majority (∼70%) of TBL1XR1 target genes is shared with SLY and SLX/SLXL1 (figs. 2*E* and 5*D*), and many of them are spermatid-expressed multicopy genes, such as *Slx/Sly*, *Mgclh*, and *Speer/Takusan* genes. In *Sly*-KD spermatids, we found that the number of TBL1XR1 genomic targets is much lower (fig. 5*C* and supplementary table S6, Supplementary Material online) and TBL1XR1 enrichment at gene starts is decreased (figs. 2*E* and 5*E*). These results indicate that TBL1XR1 enrichment at TSS depends on SLY.

## Discussion

Sex chromosome drive is presumed to occur in numerous species but characterization of this phenomenon is rare due to the usually complex organization of the genes involved and the fact that sex chromosome drive is unraveled in specific conditions, such as mating between isolated subspecies or genetic engineering. In many species such as mouse, great ape, cat, pig, human, horse, or fruit fly, sex chromosomes encode multicopy genes that are highly and/or specifically expressed during spermatogenesis (Mueller et al. 2008; Davis et al. 2015; Nam et al. 2015; Ghenu et al. 2016; Janečka et al. 2018; Lucotte et al. 2018; Bachtrog et al. 2019; Ellison and Bachtrog 2019); this genomic feature is presumed to be a consequence of an X versus Y antagonism. In the mouse, an intragenomic conflict between the sex chromosomes has long been suspected (Conway et al. 1994), and a genetic basis of this conflict has been identified in the last decade with the study of *Slx/Slxl1* and *Sly* multicopy genes, respectively, encoded by the mouse X and Y chromosomes (Cocquet et al. 2009, 2012). Although the consequences of an unbalanced numbers of *Slx/Slxl1* versus *Sly* copies (whether by knocking-down, deleting or overexpressing those genes in mouse models) on gene expression, transmission distortion, and sperm differentiation have been well described (Cocquet et al. 2009, 2012; Moretti et al. 2017; Kruger et al. 2019), the underlying molecular mechanism of the competition remained unclear. In the present study, we show that SLX/SLXL1 and SLY proteins compete for localization at the promoters of the same set of spermatid-expressed genes. Many of their target genes are multicopy, whether encoded by the sex chromosomes or by autosomes, and have been coamplified with *Slx/Slxl1/Sly* in rodents, indicating that competition between X- and Y-encoded proteins had a large impact on gene expression and content in some rodent species.

### Competition between SLX/SLXL1 and SLY to Localize at the Start of Spermatid-Expressed Genes Is Driven by Interaction with the Y Chromosome-Encoded Protein SSTY

We have previously observed that, in WT spermatids, SLY is enriched at the TSS of thousands spermatid-expressed genes whereas SLX/SLXL1 are predominantly cytoplasmic (Cocquet et al. 2009, 2012; Moretti et al. 2017). Here, we show that the small fraction of SLX/SLXL1 proteins that is nuclear is found at the same TSS as SLY, though with a weak enrichment and limited to only a few of SLY target genes. The engineered absence of SLY is necessary to fully unravel the hiding war at stake between SLX/SLXL1 and SLY. Indeed, when *Sly* is knocked down, *Slx/Slxl1* become upregulated and SLX/SLXL1 protein level in the spermatid nuclei increases; as a result the number of their target genes and enrichment at gene start are considerably higher than in WT, and SLX/SLXL1 proteins occupy the genomic sites vacated by SLY.

It has been discussed before that SLY and SLX/SLXL1 isoelectric points are too acidic to be compatible with direct interaction with DNA (Comptour et al. 2014). So how do they localize to gene starts and what drives them to these particular genomic sites? Both SLX/SLXL1 and SLY interact with H3K4me3 readers SSTY1 and SPIN1 (Comptour et al. 2014; Kruger et al. 2019) (present study), and we show here by ChIP-Seq that SSTY1, SLY, and SLX/SLXL1 proteins are enriched at the same genomic regions, that is, the TSS of thousands of expressed genes that are enriched in active chromatin marks, in particular H3K4me3 and Kcr. Searches for a consensus DNA sequence to which SLX/SLXL1 and SLY would preferentially bind failed to give a very specific motif (data not shown). Collectively, these data strongly suggest that SLY and SLX/SLXL1 enrichment at the TSS of spermatid-expressed genes is driven by interaction with SSTY, and that SSTY is an essential actor of the conflict.

SSTY belongs to the SPINDLIN protein family which also includes the autosomally encoded (and conserved through evolution) SPIN1 and several X-encoded SPIN proteins. X-linked S*pindlin* genes show a significant copy number expansion in the mouse lineage, though less impressive in numbers compared with *Ssty* hundreds of copies in *M. musculus* (Ellis et al. 2011). It remains to be determined if and how X-linked S*pindlin* genes are part of the conflict.

### Proposed Model Explaining How SLX/SLXL1 and SLY Have Opposite Effects on the Expression of Spermatid Genes

**Fig. 6.**
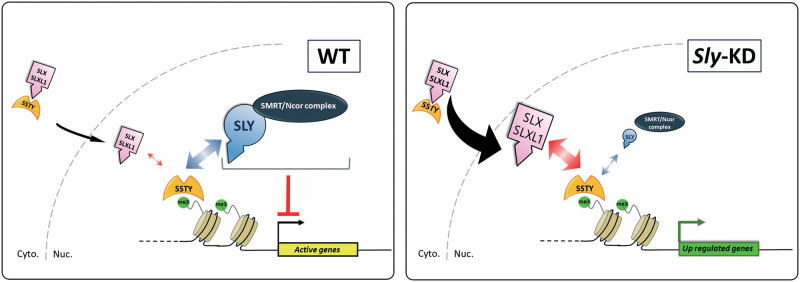
Working model: competition between SLX/SLXL1 and SLY for partner interaction and chromatin occupancy drives multicopy gene expression in mouse spermatids. SSTY proteins bind to H3K4me3-enriched TSS of expressed (active) genes in spermatids, in particular of multicopy spermatid-specific genes such as *Slx/Sly*, *Ssty*, *Mgclh*, *H2al*, and *Samt* genes on the sex chromosomes, or *Speer/Takusan* genes on autosomes. In WT spermatids (left panel), SLX/SLXL1 and SLY proteins compete for interaction with SSTY in the cytoplasm and in the nucleus. With SLX/SLXL1 being predominantly cytoplasmic, SLY occupies most of SSTY-bound TSS. SLY recruits SMRT/N-Cor repressive transcriptional complex which restrains (but does not switch off) the expression of multicopy genes. When *Sly* is knocked down (right panel), *Slx/Slxl1* genes are upregulated and SLX/SLXL1 protein level in the nuclei is higher. SLX/SLXL1 proteins are now the main partners of SSTY and, most of SSTY-bound TSS are devoid of SLY and occupied by SLX/SLXL1 proteins. As SMRT/N-Cor protein complex preferentially interacts with SLY but not with SLX/SLXL1, SMRT/N-Cor recruitment at these TSS is diminished, which leads to upregulation of target genes.

Based on the present data and others (Cocquet et al. 2009, 2010, 2012; Eberl et al. 2013; Moretti et al. 2017; Kruger et al. 2019), we propose a model explaining how SLY and SLX/SLXL1 have opposite effects on the expression of spermatid-specific genes (fig. 6). First, SSTY recognizes H3K4me3 at the TSS of spermatid-expressed genes. SLX/SLXL1 and SLY compete to interact with SSTY at these sites but, in a WT situation, most of TSS-bound SSTY proteins recruit SLY. In turn, SLY recruits SMRT/N-Cor protein complex (i.e., TBL1X, N-Cor1, and the histone deacetylase HDCA3) and limits (but does not switch off) expression of target genes. When SLY is decreased or knocked down, SLX/SLXL1 protein level is increased; SLX/SLXL1 proteins are now the main partners of SSTY and are recruited at the TSS vacated by SLY. Importantly, SLX/SLXL1 were not found to interact with SMRT/N-Cor proteins. As a result, SMRT/N-Cor recruitment at gene starts is diminished, and target genes are upregulated (fig. 6).

The fact that SLY interacts with H3K4me3 readers but recruits the repressive transcriptional complex SMRT/N-Cor reconciles the observation that SLY, which can be predominantly considered as a gene repressor, colocalizes with H3K4me3, an active chromatin mark. An interesting parallel can be drawn with the regulation of Notch target genes: Oswald et al. (2016) have indeed shown that N-Cor and the H3K4 methyltransferase KMT2D compete for interaction with SHARP, partner and corepressor (with RBP-J transcription factor) of Notch target genes.

While SLY predominant effect is to maintain/repress gene expression, SLX/SLXL1 can be viewed as activators of gene expression (Cocquet et al. 2012). The comparison of SLX/SLXL1 “direct” target genes (identified by ChIP-Seq in the present study) and the genes found deregulated (up and down) in *Slx/Slxl1* KO (knocked-out) spermatids (Kruger et al. 2019) confirms this model: There is indeed a strong positive correlation between SLX/SLXL1 target genes and the genes found downregulated in the KO (i.e., 103/335 of downregulated genes are SLX/SLXL1 targets, χ^2^, *P* < 0.0001) and an inverted correlation with upregulated genes (2/641 upregulated genes are SLX/SLXL1 targets, χ^2^, *P* = 0.0001). The mechanism by which SLX/SLXL1 activate gene expression when recruited at gene TSS remains to be investigated. Although SLX/SLXL1 may act by sole competition with the repressor SLY, it is more plausible that these proteins also recruit activators of gene expression/transcription factors. Indeed, *Slx/Slxl1* knockdown corrects (at least partially) the gene upregulation induced by *Sly* knockdown (Cocquet et al. 2012) suggesting that SLX/SLXL1 themselves have a positive effect on gene expression. It is worth noting that SLX and SLXL1 proteins may have distinct functions/molecular mechanism as suggested by Kruger et al. (2019), yet both are able to interact with SSTY and SPIN1 (Comptour et al. 2014; Kruger et al. 2019). Development of specific and ChIP-efficient antibodies should allow identify their individual chromatin profiles and contribute to address this question.

Interestingly, in a mouse model lacking the entire Y chromosome long arm (MSYq) which encompasses all copies of *Sly* and *Ssty* genes, XY spermatid genes (including *Slx/Slxl1*) are upregulated as in *Sly-*KD mice. Yet, SLX/SLXL1 protein signal in MSYq- spermatids appears restricted to the cytoplasm (Comptour et al. 2014). This observation suggests two points: First, it indicates that SSTY (or another yet to be identified MSYq-encoded product) is essential for SLX/SLXL1 presence in the nucleus. Whether SLY nuclear location relies on the same mechanism is an open question. Secondly, it suggests that factors other than SLX/SLXL1, SLY, and SSTY are involved in the regulation of the same set of genes. Functional studies of SSTY, SPIN1, and X-linked SPINDLIN should allow to expand our current model.

### SLX/SLXL1 versus SLY Competition Drives the Expression and Evolution of Spermatid-Specific Multicopy Genes Located on the Sex Chromosomes and Autosomes

Our data show that SLX/SLXL1 versus SLY competition—via their interaction with SSTY—drives the expression of hundreds of genes (thousands, if one considers one copy equals one gene), many of which are multicopy. Among them are sex chromosome-encoded spermatid-specific genes such as *Slx/Sly* themselves and *Ssty*, *Mgclh* (*Btbd35f*), and *H2al* gene families. All of them have been reported to have coamplified during mouse evolution (Ellis et al. 2011; Yogo et al. 2012; Morgan and Pardo-Manuel de Villena 2017; Molaro et al. 2018). Here, we show that appearance and massive amplification of the autosome-encoded gene family *Speer/Takusan* in rodents (>200 *Speer*/*Takusan* on *M. musculus* chromosomes 5 and 14, see also supplementary table S5, Supplementary Material online) coincide with that of *Slx/Sly* and *Ssty* genes. Similarly to the above-mentioned sex chromosome-encoded multicopy genes, *Speer*/*Takusan* are highly expressed in postmeiotic germ cells and are located in a H3K9me3-rich environment (Spiess et al. 2003; Moretti et al. 2016). Collectively, these data strongly suggest that the X versus Y intragenomic conflict in which *Slx/Slxl1*, *Sly*, and *Ssty* are engaged has significantly influenced expression and copy number of several sex chromosome-encoded as well as autosomal genes in rodents.

There are several lines of evidence indicating that this intragenomic conflict also contributes to hybrid incompatibility and lineage divergence. F1 hybrid males from interspecific crosses between *M. musculus* females and *M. domesticus* males are sterile; because *M. domesticus* Y chromosome encompasses fewer *Sly* copies than *M. musculus* Y chromosome, these F1 sterile^MxD^ hybrid males have an unbalanced *Slx/Slxl1* versus *Sly* copy number, mimicking *Sly* knockdown. Their phenotypes include sex ratio distortion, spermatogenesis defects, and male hypo/infertility and are associated with X chromosome gene upregulation, as well as *Speer/Takusan* upregulation (Good et al. 2010; Larson et al. 2017), similarly to what is observed in *Sly* knocked-down mice (Cocquet et al. 2009; Moretti et al. 2017). Importantly, in a naturally occurring house mouse hybrid zone (i.e., between *Mus musculus musculus* and *M. m. domesticus*), sex ratio distortion and reduced male fertility are also observed (Macholan et al. 2008; Turner et al. 2012) and associated with an excess of *Slx/Slxl1* versus *Sly* copy number.

Our present findings demonstrate that another Y-encoded multicopy gene, *Ssty*, which appeared on the rodent Y chromosome before *Sly* (Ellis et al. 2007, 2011), is involved in this conflict and has coamplified with *Speer/Takusan* genes. Imbalance between *Ssty* copy number and that of X or autosomal multicopy genes should therefore be considered when investigating hybrid incompatibility/lineage divergence and could impact rodent species other than mice, because both *Ssty* and *Speer/Takusan* are multicopy in the rat.

### *Smok* Autosomal Transmission Responder Is a Target of the Mouse X versus Y Intragenomic Conflict

Our observation that *Smok* genes (i.e., *Smok2a*, *Smok2b*, *Smok3a*, etc.) are enriched in SLX/SLXL1 and SSTY1 (but not in SLY) is intriguing as a mutant form of *Smok* on chromosome 17 (called *Smok^Tcr^*) has been shown to be the transmission responder *Tcr* in one of the best characterized examples of autosomal transmission distortion in mice, the *t-*haplotype (Herrmann et al. 1999; Lyon 2003). There are several other similarities between t-haplotype system and *Sly* versus *Slx/Slxl1* conflict. In the *t-*haplotype system, the same loci can give rise to transmission distortion and sterility depending on gene dosage of distorters: heterozygosity of distorters being associated with transmission distortion and decreased fertility whereas homozygosity being associated with sterility (Herrmann et al. 1999). Similarly, unbalanced copy numbers of *Sly* versus *Slx/Slxl1* lead to transmission distortion and decreased fertility, when mild, and to sterility, when severe (Moriwaki et al. 1988; Styrna et al. 1991; Burgoyne et al. 1992; Conway et al. 1994; Touré et al. 2004). In both cases, transmission distortion is associated with sperm motility defects: The motility of sperm bearing a Y chromosome with a partial MSYq deletion (and therefore decreased level of *Sly* and *Ssty*) is lower than that of X-bearing spermatozoa (Grzmil et al. 2007; Rathje et al. 2019); and in males heterozygous for the *t-*haplotype region, the motility of WT sperm is poorer than that of *Tcr*-bearing spermatozoa. The *t*-haplotype is a typical example of a killer meiotic drive system, in which selfish genes/loci increase their own transmission via destroying noncarrier gametes (Herrmann et al. 1999; Veron et al. 2009; Lindholm et al. 2016; Bravo Nunez et al. 2018). The underlying mechanism of transmission distortion in *Slx/Slxl1* versus *Sly* is not understood, but the genetic interaction with *t-*haplotype genes should be interesting to investigate.

### SLX/SLXL1/SLY/SSTY Target Genes Are Potential Transmission Distorters

As said above, many of SLX/SLXL1/SLY/SSTY target genes are spermatid-expressed multicopy genes, whether sex chromosome-encoded or autosomal. This can be viewed as a way to globally control the expression of potential TDs, which would act via increasing their own expression and gene dosage. Identification of *Smok* genes among SLX/SLXL1/SLY/SSTY target genes adds further evidence to the hypothesis that *Slx/Sly* system could be intertwined with other transmission distortion systems.

Interestingly, a recent analysis of spermatid-expressed transcripts at the single-cell level has identified thousands of genes which are not equally shared among spermatids, called GIMs for GenoInformative Markers (Bhutani et al. 2019). Comparisons with our data reveal that more GIMs than non-GIMs are found among SLX/SLXL1/SLY/SSTY targets—this is true for autosomal genes (*P* < 2.2 × 10^−16^) as well as X-encoded genes (*P* = 2.8 × 10^−4^). Of note, multicopy genes are not specifically addressed in GIM data set, yet one can find an enrichment of *Speer*/*Takusan* (31 confident GIMs vs. 83 nonconfident/remaining, *P* = 2.9 × 10^−9^) and *Smok* genes (8 confident GIMs vs. 0 nonconfident/remaining genes, *P* = 2.7 × 10^−7^) among GIMs versus non-GIMs. Therefore, SLX/SLXL1/SLY/SSTY control the expression of several sex chromosome-encoded and autosome-encoded genes, products of which are not equally shared among spermatids, and could therefore be transmission distorters and/or responders.

It is worth mentioning that we did not find the X-encoded *Toll-like receptor Tlr7* and *Tlr8*, which have been recently implicated in sperm motility and sex ratio distortion in the mouse (Umehara et al. 2019; Navarro-Costa et al. 2020), among SLX/SLXL1, SLY, or SSTY target genes, suggesting distinct pathways.

In conclusion, our study provides a better comprehension of the X versus Y chromosome conflict which exists in mice and suggests that it may occur in other rodents. Indeed, we show evidence for a direct competition between X- and Y-encoded proteins (i.e., SLX/SLXL1 and SLY) at the promoters of the genes they regulate and identify another Y-encoded protein, SSTY, as a key element of this competition. Importantly, whereas *Sly* is restricted to the mouse lineage, acquisition of *Ssty* predated that of *Sly* (Ellis et al. 2007, 2011), and its presence correlates with that of *Speer/Takusan*, the intriguing spermatid-expressed autosomal gene family which has been remarkably amplified in some rodents. Our data also indicate that other TDs, whether sex chromosome-encoded or autosomal, are among SLX/SLXL1/SLY/SSTY target genes, in particular genes of the *t-*haplotype system. Further studies will be needed to understand the involvement of these genes, specifically of *Ssty* and *Speer/Takusan*, in the conflict, and the evolutionary impact they may have had on rodent species other than mice.

Finally, X versus Y antagonism has been previously described in other species (mainly *Drosophila*), and competition at the molecular level often appears to be driven by RNA interference (Bachtrog et al. 2019; Courret et al. 2019). In the model described here, X versus Y antagonism is mediated at the protein level, but the picture is likely more complex and one cannot exclude contribution of noncoding RNA.

## Materials and Methods

### Mice

All animals used in the present study were on >90% C57BL/6 background and processed at adult age (between 2- and 6-month-old males). *Sly-*KD and Flag-SLY mice were obtained as described in Cocquet et al. (2009) and Moretti et al. (2017). For all experiments, WT controls were of same age and background (i.e., nontransgenic siblings from the same mating). Animal procedures were subjected to local ethical review (Comite d’Ethique pour l’Experimentation Animale, Universite Paris Descartes; registration number CEEA34.JC.114.12, APAFIS 14214-2017072510448522v26).

### Germ Cell Purification by FACS or Elutriation

Round spermatids were purified from adult testis by fluorescence-activated cell sorting (FACS) or elutriation. For FACS-based cell separation, testicular cells were isolated from one adult male per experiment following a protocol adapted from Bastos et al. (2005) and described in Comptour et al. (2014).

For centrifugal elutriation-based cell separation, testicular cells were isolated from two to three adult mice per experiment, as described previously (Cocquet et al. 2009). Fractions enriched in different testis cell types were separated with a J6-ME elutriator (Beckman) with conditions described in Cocquet et al. (2009). Following FACS or elutriation, collected cells were washed in 1× Phosphate-buffered saline (PBS), then cell pellets were either directly snapped frozen in liquid nitrogen or fixed in 1% formaldehyde (SIGMA) for 10 min in PBS, then incubated with 125 mM glycine, washed twice with PBS prior to being snapped frozen in liquid nitrogen and transferred to -80 °C. To assess cell purity in isolated fractions, ∼50,000 cells were spread onto a superfrost slide and fixed with 4% buffered paraformaldehyde (PFA) for 10 min. After three washes in PBS, slides were dried and mounted in a medium containing DAPI (4,6-diamidino-2- phenylindole) (VECTASHIELD Mounting Medium with DAPI, Vectorlab, Burlingame, CA). Round spermatids were recognized based on their typical nucleus morphology and size (i.e., round spermatids have a small and round nucleus characterized by the presence of one or two chromocenters (see, e.g., Comptour et al. [2014] for pictures).

### ChIP-Seq

ChIP-Seq was performed to assess genomic location of SLX/SLXL1, SSTY1, and TBL1XR1 (see parameters in supplementary tables S1–S3 and S6, Supplementary Material online). The data sets generated and/or analyzed during the current study are available in the SRA repository (https://www.ncbi.nlm.nih.gov/sra/ ; last accessed April 30, 2020) with the following project numbers: PRJNA627396, PRJNA275694, PRJNA62757, and PRJNA643726.

To compare SLX/SLXL1- and SLY-binding sites, SLX/SLXL1 ChIP-Seq was performed on the same WT spermatid chromatin samples and under the same conditions as previously reported for SLY ChIP-Seq (Moretti et al. 2017). In brief, ∼10 million of FACS-sorted enriched fractions of WT round spermatids (with a purity >90%) were fixed with 1% formaldehyde for 15 min then quenched with 125 mM glycine. Chromatin was isolated by adding lysis buffer, followed by disruption with a Dounce homogenizer. Lysates were sonicated and the DNA sheared to an average length of 300–500 bp. An aliquot of chromatin (30 μg) was precleared with protein A agarose beads (Invitrogen). Genomic DNA regions of interest were isolated using 12 μg of antibody against SLX/SLXL1 (Reynard et al. 2007). Complexes were washed, eluted from the beads with SDS buffer, and subjected to RNase and proteinase K treatment. Crosslinks were reversed by incubation overnight at 65 °C, and ChIP DNA was purified by phenol–chloroform extraction and ethanol precipitation. Preparation and sequencing of the library were performed as described previously (Moretti et al. 2017), and the input used was the same WT round spermatid sample used in Moretti et al. (2017) (see supplementary table S1, Supplementary Material online).

To compare SLX/SLXL1-binding sites in WT and *Sly-*KD round spermatids, three independent experiments were performed using iDeal-ChIP-Seq for transcription factor kit (Diagenode). Experimental procedures differed between experiments (see supplementary table S2, Supplementary Material online, for details). Best results (highest number of peaks, lowest False Discovery Rate) were obtained using experimental condition 1. In brief, 10 million of WT or *Sly-*KD elutriated round spermatids were frozen, then fixed with 1% formaldehyde for 10 min then quenched with 125 mM glycine. After PBS wash, cells were sonicated in iS1b shearing buffer (with protease inhibitor cocktail, iDeal-ChIP-Seq for transcription factor kit, Diagenode) for 1 × 8 cycles (30 s “ON,” 30 s “OFF”) using Bioruptor Pico sonication device (Diagenode Cat# B01060001). Following a 10-min centrifugation at 16,000 × g, supernatants were collected and mixed with beads and antibody, following instructions from the manufacturer (iDeal-ChIP-Seq for transcription factor kit; Diagenode). Two micrograms of antibody against SLX/SLXL1 (Reynard et al. 2007) was used per 10 million of cells. Following purification, DNA was resuspended with 20 µl of water. DNA concentration was quantified using Qubit ds DNA HS kit (Thermo Fisher Scientific, Q32854). Libraries were then prepared from input and immunopurified DNA with 1 ng as starting material using MicroPlex Library Preparation Kit v2 (Diagenode Cat# C05010013), then purified using Agencout AMPPure XP (Beckman Coulter) and quantified using Qubit dsDNA HS Assay Kit (Thermo Fisher Scientific, Q32854). Libraries were pooled equimolarly and sequenced by Diagenode on an Illumina Hiseq 2500 or 3000/4000 instrument with 50-bp single-end reads at a depth of coverage per sample ranging from 30M to 70M. SSTY1 and TBL1XR1 ChIP-Seq analyses on WT and *Sly-*KD round spermatids were performed using the same protocol (see supplementary tables S3 and S6, Supplementary Material online, for details).

### Bioinformatic Analyses

#### ChIP-Seq Analyses

To compare SLX/SLXL1 genomic location with that of SLY in WT round spermatids, the bioinformatic analysis was performed using the parameters described in Moretti et al. (2017) as follows. SLX/SLXL1 ChIP sequences (50-nt reads, single end) were aligned to the mouse genome (GRCm38/mm10) using Burrows Wheeler Alignment (BWA) algorithm version 0.6.1 (Li and Durbin 2010). For reads with multiple good alignments, one alignment was reported at random. Alignments were extended in silico at their 3′-ends to a length of 200 bp, and assigned to 32-nt bins along the genome. SLX/SLXL1 peaks were called using MACS algorithm (v1.4.2) (Zhang et al. 2008) with a cutoff of *P* = 1e − 7 (see supplementary table S1, Supplementary Material online).

All other ChIP-Seq (and corresponding input sequences) were analyzed as follow. Reads were aligned to the mouse genome (GRCm38/mm10) using BWA algorithm version 0.7.12-r1039 (Li and Durbin 2010) with “mem” command. Reads which do not map uniquely on the genome are distributed to one location picked randomly. Briefly, BWA-MEM algorithm works by seeding alignments with maximal exact matches (MEMs) and then extending seeds with the affine-gap Smith–Waterman algorithm. In case of multiple primary alignments, -c INT discards an MEM if it has more than INT occurrence in the genome. The maximum is set to 10,000. Peaks were then called with MACS (Zhang et al. 2008) using default parameters (*P* value cutoff for peak detection = 1e − 05; mfold parameters = 10.32) (see supplementary tables S2, S3, and S6, Supplementary Material online).

Analysis of published round spermatids ChIP-Seq data sets were performed using the same parameters. Data sets for H3K4me3, H3K27me, Kcr, H3K9ac, H4ac, H3K27ac, 5hmC, and H3K9me3 were acquired from several published reports (Hammoud et al. 2009; Tan et al. 2011; Erkek et al. 2013; Gan et al. 2013; Bryant et al. 2015).

Graphic representation of ChIP-Seq comparisons was done using Venn Diagram tool (available at http://cistrome.dfci.harvard.edu/ap/root; last accessed April 30, 2020). Heatmaps were produced using ComputeMatrix and Plot HeatMap tools (Ramirez et al. 2016) (available at the Galaxy website: https://mississippi.snv.jussieu.fr/; last accessed April 30, 2020). Graphic representations of ChIP-Seq data were performed using IGV (Integrative nomics Viewer, https://www.broadinstitute.org/igv/; last accessed April 30, 2020). Comparison of ChIP-Seq data sets was performed by comparing the number of intervals in common and performing a χ^2^ statistical test.

#### Gene Analyses

BED files containing ChIP-Seq regions were intersected with Ensembl80 gene coordinates (TSS ± 1 kb). Graphical representation of enrichment around the start of genes (expressed and not expressed in round spermatids) was done using Profiler and ComputeMatrix tools (Ramirez et al. 2016) (available at the Galaxy website https://mississippi.snv.jussieu.fr/; last accessed April 30, 2020) and round spermatids RNA-Seq data from Gan et al. (2013) reanalyzed using the last release of the mouse genome mm10 and gene annotation Ensembl77 (Moretti et al. 2016). Correlation with gene expression was performed using the same data sets. Venn diagrams were drawn using Venn Diagram Plotter. It is important to keep in mind that the number of gene copies annotated in the reference genome may not be exact (and indeed differs between versions of genome annotation).

Multicopy gene families (fig. 2) were searched using Protein Family ID from Ensembl Biomart 93. Families with eight or more members and among SLX/SLXL1 target genes (common to the three ChIP-Seq replicates performed in *Sly-*KD spermatids) were kept, that is, *Sycp3-*related gene family (PTHR19368, all genes except *Sycp3* were found on chrX and chrY), *Takusan* gene family (PTHR21558, most genes were found on chr5 and 14), *Spindlin* gene family (PTHR10405, all genes except *Spin1* were found on chrX and chrY), *H2al* gene family (PTHR23430_SF11, all genes except *H2al2a* were found on chrX), *Samt* gene family (PTHR12002_SF76, all genes were found on chrX), *Btbd35f* gene family (aka *Mgclh*, PTHR23231_SF3, all genes were found on chrX), and *Smok* gene family (genes found on various autosomes—including chr5 and 17). Comparison of the number of coding genes for *Sypc3*, *Spindlin*, and *Speer/Takusan* families between *M. musculus* and other rodents (fig. 4) was performed using Ensembl99 CrossSpecies Protein Family tool.

The comparison of SLY, SLX/SLXL1, and SSTY target genes (TSS ± 1 kb) (fig. 4*C* and supplementary table S4, Supplementary Material online) was performed using SLY target genes from SLY ChIP-Seq on WT round spermatids from Moretti et al. (2017) and SLX/SLXL1 and SSTY1 target genes from all SLX/SLXL1 ChIP-Seq and SSTY1 ChIP-Seq data sets performed on WT and *Sly-*KD round spermatids. All data sets (including SLY ChIP-Seq) were analyzed with the parameters indicated in supplementary tables S2 and S3, Supplementary Material online.

Comparison of SLX/SLXL1 target genes (genes identified by ChIP-Seq, in common in the three *Sly*-KD replicates) with genes that are deregulated in *Sly*-KD spermatids was performed using microarray data from Cocquet et al. (2009) and reanalyzed in Moretti et al. (2017) using Ensembl77.

Comparison of SLX/SLXL1 target genes with genes that are deregulated in *Slx/Slxl1* KO spermatids was performed using data published in Kruger et al. (2019).

Comparison of SLX/SLXL1/SLY/SSTY1 target genes with GIMs was performed using the list of common 2,424 target genes (supplementary table S4, Supplementary Material online) and data from Bhutani et al. (2019) accessible at bioRxiv: https://www.biorxiv.org/ (last accessed April 30, 2020). GIMs and non-GIM controls were taken from supplementary tables S1 and S3 of Bhutani et al.(2019), respectively. Sex chromosome GIMs taken from table 2 were supplemented by a set of confident non-GIMs on sex chromosomes (shown outside the main clusters in supplementary figure S3 of Bhutani et al. [2019]). *Speer/Takusan* genes were all those from PFAM family ID PF04822. For autosomal GIMs, the significance of overlaps was calculated for each of three control sets: random genes (disregarding expression in spermatogenesis), spermatid-expressed non-GIM controls, and expression-matched non-GIM controls using a *z*-test based on the mean and standard deviation of the overlap in 20 random control sets. To be as conservative as possible, the least significant *P*-value across these comparisons was reported. For sex chromosomes, there were no expression-matched controls available so the overlap for GIMs was compared with non-GIMs using a Fisher’s exact test.

### ChIP-qPCR and RT-qPCR

For ChIP, input and immunoprecipitated purified DNA were diluted and analyzed by RT-qPCR using Roche LightCycler 480 and SensiFast No-Rox kit mix (Bioline) following instructions from the manufacturer. Primers were designed to amplify regions across the TSS of indicated genes, except for NC and NC2 which represent negative control regions located, respectively, 170 and 40 kb away from any TSS. The sequences and qPCR condition for *Actrt1* ChIP primers can be found in Soboleva et al. (2012) (for other primers, see supplementary table S7, Supplementary Material online).

RT-qPCR was performed on RNA extracted from WT- and *Sly-*KD-elutriated round spermatids and reversed-transcribed as described in Cocquet et al. (2009). The sequences and qPCR conditions for *β-actin* primers are from Cocquet et al. (2009), for *Slx* primers are from Ellis et al. (2005), for *Actrt1* primers are from Cocquet et al. (2012), for *H2afb3* primers are from Soboleva et al. (2012), and for *Dot1l* H9LAN primers are from Dottermusch-Heidel et al. (2014) (for other primers, see supplementary table S7, Supplementary Material online). Student’s *t*-test was used for all qPCR statistical analyses.

### Protein Extraction from Testes

Protein extractions from testes were performed as previously described in Moretti et al. (2017). Briefly, flash-frozen testes were ground and resuspended in 1:9 w/v ice-cold extraction buffer (20 mM Tris/HCl pH 8.0, 150 mM NaCl, 5 mM ethylenediaminetetraacetic acid, 0,5% Igepal A-630 [Sigma-Aldrich], 1× protease inhibitor cocktail [Sigma-Aldrich], 1 mM phenylmethanesulfonyl fluoride, and 1 mM sodium orthovanade). After 30 min of incubation at 4 °C, samples were centrifuged at maximum speed for 10 min. The supernatant was collected and immediately used for immunoprecipitation.

### Co-immunoprecipitation Assay

Co-immunoprecipitation assays against FLAG-tagged SLY were performed as previously described in Moretti et al. (2017) with minor modifications. Briefly, 20 µl packed gel volume, corresponding to 40 µl of magnetic beads, was used for one immunoprecipitation experiment. Beads were washed with Tris-buffered saline (TBS) solution (50 mM Tris/HCl pH 7.4, 150 mM NaCl). Cell lysate was added to the magnetic beads and incubated for 2 h at 4 °C on a rotating wheel. Beads were washed two times with TBS-0.5% Tween solution before target protein elution. Elution was performed using 0.1 M glycine/HCl, pH 3.0. The pH was neutralized using triethylammonium bicarbonate buffer (pH 8.4).

For other proteins, co-immunoprecipitation assay was performed as previously described in Moretti et al. (2017) with minor modifications. Briefly, for each immunoprecipitation experiment, 2 µg of antibodies against SLY (Reynard et al. 2009), against SLX/SLXL1 (Reynard et al. 2007), against SPIN1 (orb193004, Biorbyt) and 2 µl of antibodies against TBL1XR1 (ab24550, Abcam) were used with 20 µl of protein A/G-coated beads (PAG 0463, Ademtech). Beads were washed three times in 1× PBS pH 7.5 with 0,65% Tween, prior to antibody incubation at 4 °C for 30 min under agitation. Beads were then incubated 30 min at room temperature in 20 mM dimethyl pimelimidate pH 9 and crosslinking reaction was quenched using 50 mM Tris/HCl pH 7.5. Antibody coupled beads were incubated with whole-testis extracts for 1 h and washed three times in 1× PBS pH 7.5 with 0,65% Tween. Elution was performed using 0.1 M glycine/HCl, pH 3.0. The pH was neutralized using triethylammonium bicarbonate buffer (pH 8.4).

### Western Blot

Western blot experiments on whole testes were performed as described in Comptour et al. (2014) and Moretti et al. (2017). Fifteen microliters of immunoprecipitated samples and 5 µl of input were denatured in 1× NuPAGE LDS sample buffer (Life Technologies) at 95 °C during 10 min. Antibody against SLY (Reynard et al. 2009) and against SLX/SLXL1 (Reynard et al. 2007) was diluted 1/3,000; anti-TUBULIN was diluted 1/5,000 (05-661, Millipore); and anti-TBLX1R1 (ab24550, Abcam), anti-HDAC3 (ab7030, Abcam), anti-TBL1X (ab24548, Abcam), anti-SSTY1 (Comptour et al. 2014), anti-SPIN1 (orb193004, Biorbyt), and anti-NCor1 (ab3482, Abcam) were diluted 1/1,000.

### Immunohistochemistry

Colorimetric (DAB, 3,3′-diaminobenzidine) histochemistry was performed as described in Comptour et al. (2014) with minor modifications and using the Novolink polymer detection system (7140-K, Leica Micro-systems). In brief, 4-µm-paraffin sections of testes were dewaxed in xylene and rehydrated in a graded series of alcohol baths. The sections were first incubated for 30 min at 95 °C and then incubated for 10 min at room temperature in 0.01 M sodium citrate solution (pH 6) for antigen retrieval. Slides were washed under running water for 10 min and incubated for 30 min in a blocking solution (1× PBS, 1% BSA, 0.1% Triton). Antibody against TBL1XR1 (ab24550, Abcam) and TBL1X (ab24548, Abcam) was diluted at 1/50. Using a wet chamber, slides were incubated overnight at 4 °C with antibody diluted in blocking buffer. The subsequent stages were performed as described by the manufacturer (Novolink polymer detection, Leica), and the sections were counterstained with Mayer’s hematoxylin solution for 2 min (Merck, Millipore). Sections were dehydrated in a graded series of alcohol bath and a final xylene bath.

## Supplementary Material

Supplementary data are available at *Molecular Biology and Evolution* online.

## Supplementary Material

msaa175_supplementary_data
