# Supplementary material for: Battle of the Sex Chromosomes: Competition between X and Y Chromosome-Encoded Proteins for Partner Interaction and Chromatin Occupancy Drives Multicopy Gene Expression and Evolution in Muroid Rodents
Source: Mol Biol Evol. 2020 Jul 13;37(12):3453–68. doi: 10.1093/molbev/msaa175 (PMC7743899; doi:10.1093/molbev/msaa175)
Supplement: msaa175_supplementary_data [file msaa175_supplementary_data.pdf]

Supporting Figure Legend

Figure S1. Complement to Figure 1A. Graphic representation of the comparison between SLX/SLXL1 ChIP-Seq and ChIP-Seq datasets from 8 chromatin marks (Kcr, H3K4me3, H3K79me2, H3K9ac, H4ac, H3K27ac, H3K27me3 and H3K9me3) and from SLY, all performed in wild type round spermatids. Numbers indicate the number of intervals in common between SLX/SLXL1 ChIP-Seq and other datasets; the red lines represents values expected at the genome level by chance for each mark. All of them show a significant difference with theoretical values (3 stars, $\chi 2$= P<0.0001). For H3K27me3, the correlation is inverse indicating that SLX/SLXL1 is depleted in H3K27me3-enriched regions.

Figure S2. Complement to Figure 2. A. Venn diagrams representing the overlap between the 3 ChIP-Seq experiments using anti-SLX/SLXL1 antibody in WT and *Sly*-KD round spermatids. B. Venn diagrams showing SLX/SLXL1 genomic location overlap between WT and *Sly*-KD round spermatids for each experiment. C. Graphic representation of SLX/SLXL1 genomic location (% of coverage of each chromosome) as identified by ChIP-Seq on WT and *Sly*-KD round spermatids for each of the 3 sets of experiments. D. Venn diagrams showing the overlap between SLX/SLXL1 target genes in WT and *Sly*-KD round spermatids for each experiment.

Figure S3. Complement to Figure 2. A. Graphic representation of SLX/SLXL1 ChIP-Seq profile in WT (black lines) and *Sly*-KD (red lines) round spermatids showing the average enrichment of SLX/SLXL1 around the TSS of genes expressed (unbroken lines) and not expressed (dotted lines) in round spermatids, for each set of experiment. B. Graphic representation of the chromosome location of SLX/SLXL1 target genes (TSS +/-1kb) as identified by ChIP-Seq on WT and *Sly*-KD round spermatids for each of the 3 sets of experiments. C. IGV screenshot showing SLX/SLXL1 ChIP profile in WT and *Sly*-KD round spermatids for all 3 independent experiments. The genomic region shown is a portion of chromosome 14 encompassing *Gm10377*, a gene of *Takusan* family.

Figure S4. A. Schematic of gene family expression as found by RNA-Seq on Sertoli cells (SE) and mouse purified germ cells fractions (analysis of GSE3500518; (Gan, et al. 2013) described in (Moretti, et al. 2016)). PriSG-A: primitive spermatogonia; SG-A: A spermatogonia; SG-B: B spermatogonia; lepSC: leptotene spermatocytes; pacSC: pachytene spermatocytes; rST: round spermatids; eST: elongating spermatids. The Y-axis indicates RPKM values.

Figure S5. A. Alignment of SSTY1 (NP_033246), SSTY2 (AAH87891) and SPIN1 (NP_666155) mouse proteins with CLUSTALW. The 3 Spin/Tudor-like domains are underscored. In pink: residues forming the H3K4me3-binding aromatic cage of Spin/Tudor-like domain II. In green: residues important for or facilitating binding to H3R8me2a. In yellow, other residues predicted to be important for H3K4me3 recognition [from (Yang, et al. 2012; Su, et al. 2014)]. B. Western blot detection with SLY, SLX/SLXL1 or SPIN1 antibody following immunoprecipitation (IP) with SLX/SLXL1 or SLY antibody on wild-type (WT) or *Sly*-KD testis. Immunoprecipitation with IgG was used as negative control. Input samples represent protein extracts before immunoprecipitation. Arrows indicate bands at the right size. SPIN1 is immunoprecipitated with anti-SLX/SLXL1 antibody (upper panel) or anti-SLY antibody (bottom panel). C. Western

blot detection with SLY antibody following immunoprecipitation (IP) with SLX/SLXL1 antibody on wild-type (WT) or *Sly*-KD testis. Immunoprecipitation with IgG was used as negative control. Input samples represent protein extracts before immunoprecipitation. Arrows indicate bands at the right size. SLY is not pulled-down by anti-SLX/SLXL1 antibody.

Figure S6. Complement to Figure 4. A. Venn diagrams representing the overlap between SSTY1 ChIP-Seq replicates (Exp1 and 2) in WT and *Sly*-KD round spermatids. B. Graphic representation of SSTY1 genomic location (% of coverage of each chromosome) as identified by ChIP-Seq on WT and *Sly*-KD round spermatids for each of the 2 sets of experiments (Exp1 and Exp2). C. Venn diagrams showing SSTY1 ChIP-Seq genomic location overlap between WT and *Sly*-KD round spermatids for experiment 1 (Exp1), experiment 2 (Exp2) and regions in common between the two replicates. D. Graphic representation of the chromosome location of SSTY1 target genes (TSS +/-1kb) as identified by ChIP-Seq on WT and *Sly*-KD round spermatids for Exp1, Exp2 and found in both experiments (merge of both replicates). E. Venn diagrams representing the percentage of SSTY1 target genes in common in WT and *Sly*-KD round spermatids (merge of 2 replicates). F. Genomic annotation of SLY, SLX/SLXL1 and SSTY1 ChIP-Seq. Data shown are of SLY ChIP-Seq in WT (Moretti, et al. 2017), SSTY1 (Exp1) and SLX/SLXL1 (Exp1) ChIP-Seq in WT or *Sly*-KD round spermatids. G. Venn diagrams representing the number of common target genes between SSTY1 (Exp1), SLX/SLXL1 (Exp1), and SLY in WT (Moretti, et al. 2017), or between SSTY1 (Exp1) and SLX/SLXL1 (Exp1) in *Sly*-KD round spermatids. H. Heatmap comparison of SLY ChIP-Seq with SSTY1 (Exp1 and 2) and SLX/SLXL1 (Exp1, 2 and 3) ChIP-Seq datasets, performed in wild type (WT) or *Sly*-KD (KD) round spermatids. Each heatmap shows the scores (k-means) associated with SLY-enriched genomic regions (1kb downstream to 1kb upstream the center of the interval). The color code associated with enrichment values is indicated on the right. I. IGV screenshot showing SLY ChIP profile in WT round spermatids compared to its input (from (Moretti, et al. 2017)), SLX/SLXL1 (Exp1) and SSTY1 (Exp1) ChIP profiles in WT and Sly-KD round spermatids. The genomic regions shown are portions of chromosome X encompassing *Slxl1,* of chromosome Y showing *Sly* and *Ssty2,* of chromosome 5 showing *Speer4a,* of chromosome 17 showing *Smok2a* and *Smok2b,* and of chromosome 10 showing *Sycp3. Slxl1, Sly, Ssty2, Speer4a* are target genes of SLY, SLX/SLXL1 and SSTY1; *Smok2a* and *Smok2b* are target genes of SLX/SLXL1 and SSTY1; and *Sycp3* is a target gene of SLY only.

Figure S7. A. Expression of *Tbl1xr1* and *Tbl1x* in mouse tissues based on mouse ENCODE transcriptome data (obtained from NCBI). The Y-axis indicates RPKM values. B. Schematic of *Tbl1xr1* and *Tbl1x* expression as found by RNA-Seq on Sertoli cells (SE) and mouse purified germ cells fractions (analysis of GSE3500518; (Gan, et al. 2013) described in (Moretti, et al. 2016)). PriSG-A: primitive spermatogonia; SG-A: A spermatogonia; SG-B: B spermatogonia; lepSC: leptotene spermatocytes; pacSC: pachytene spermatocytes; rST: round spermatids; eST: elongating spermatids. The Y-axis indicates RPKM values. C. Immunohistochemistry detection of TBL1XR1 and TBL1X proteins in WT and and *Sly*-KD testicular sections. TBL1XR1 and TBL1X signals were revealed with DAB (3,3'-diaminobenzidine) and hematoxylin was used

to counterstain nuclei. ‘P’ stands for pachytene spermatocytes, ‘M’ stands for spermatocytes in metaphase, ‘RS’ for round spermatids, ‘ES’ for elongating spermatids, and ‘CS’ for condensing spermatids. Pictures were taken using the same parameters. Scale bar indicates 20µm. D. Western blot analysis of TBL1XR1 in WT and *Sly*-KD testis. Anti-tubulin (TUB) was used to normalize the samples. Ponceau staining is also shown underneath to confirm that similar protein quantities were loaded in each well. No significant change in TBL1XR1 level was observed.

Figure S1

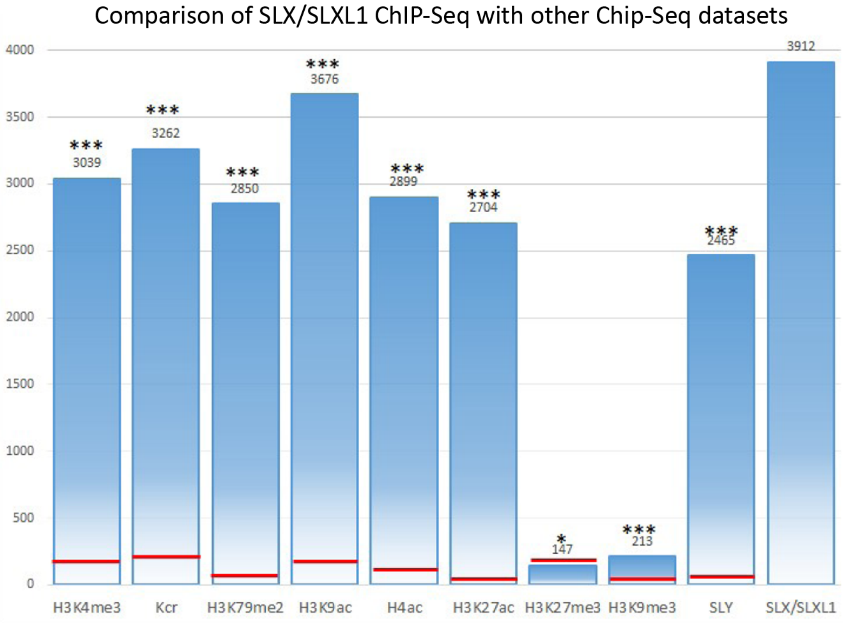

Comparison of SLX/SLXL1 ChIP-Seq with other Chip-Seq datasets
4000
3500
3000
2500
2000
1500
1000
500
0
***
3039
***
3262
***
2850
***
3676
***
2899
***
2704
*
147
***
213
***
2465
3912
H3K4me3
Kcr
H3K79me2
H3K9ac
H4ac
H3K27ac
H3K27me3
H3K9me3
SLY
SLX/SLXL1

Figure S2

A

Overlap between the 3 replicates of SLX/SLXL1 ChIP-Seq

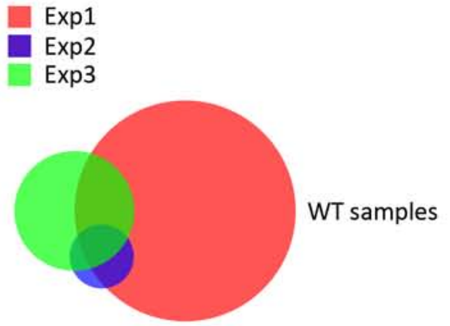

WT replicates	Exp1	Exp2	Exp3
Exp1	100%	21%	17%
Exp2	68%	100%	31%
Exp3	50%	35%	100%

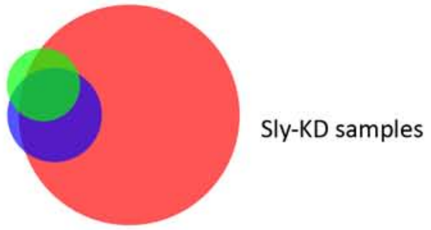

Sly-KD replicates	Exp1	Exp2	Exp3
Exp1	100%	31%	12%
Exp2	92%	100%	31%
Exp3	95%	83%	100%

B

SLX/SLXL1 genomic location

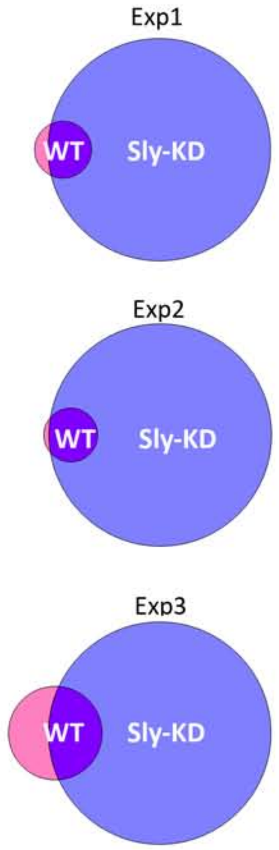

C

SLX/SLXL1 genomic location in WT and Sly-KD round spermatids (ChIP-Seq)

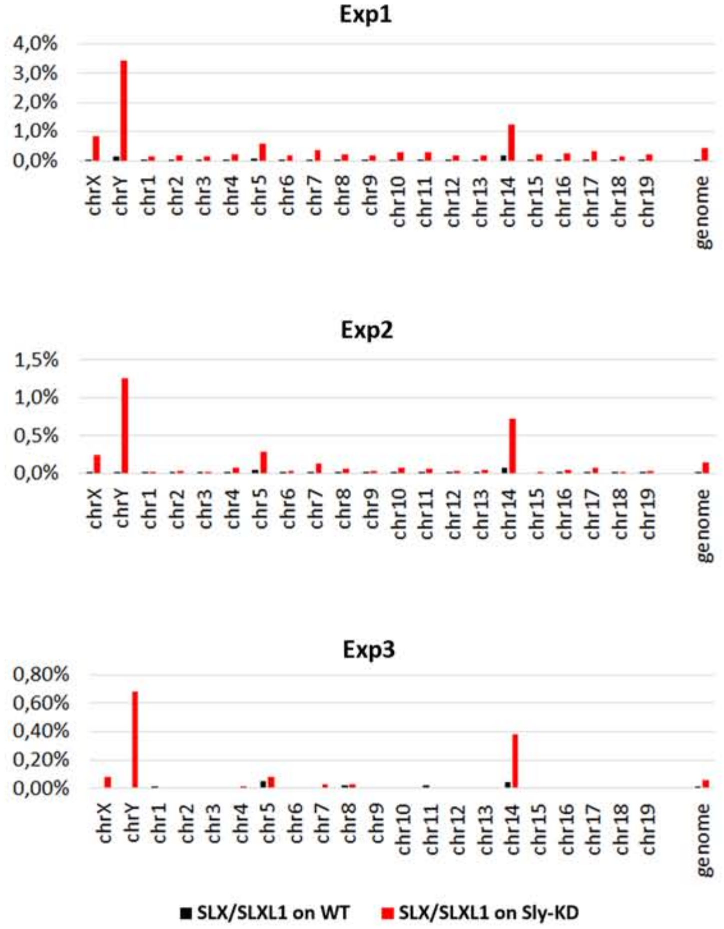

D

SLX/SLXL1 target genes

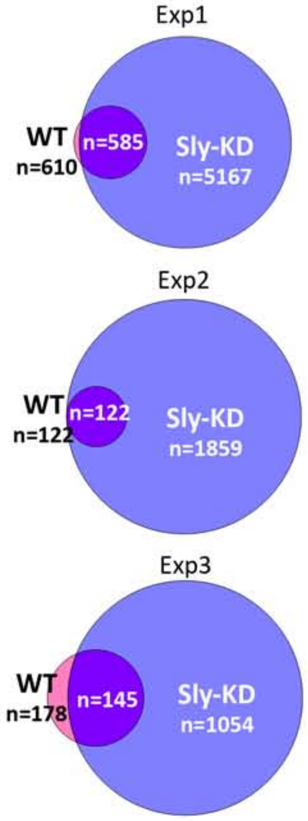

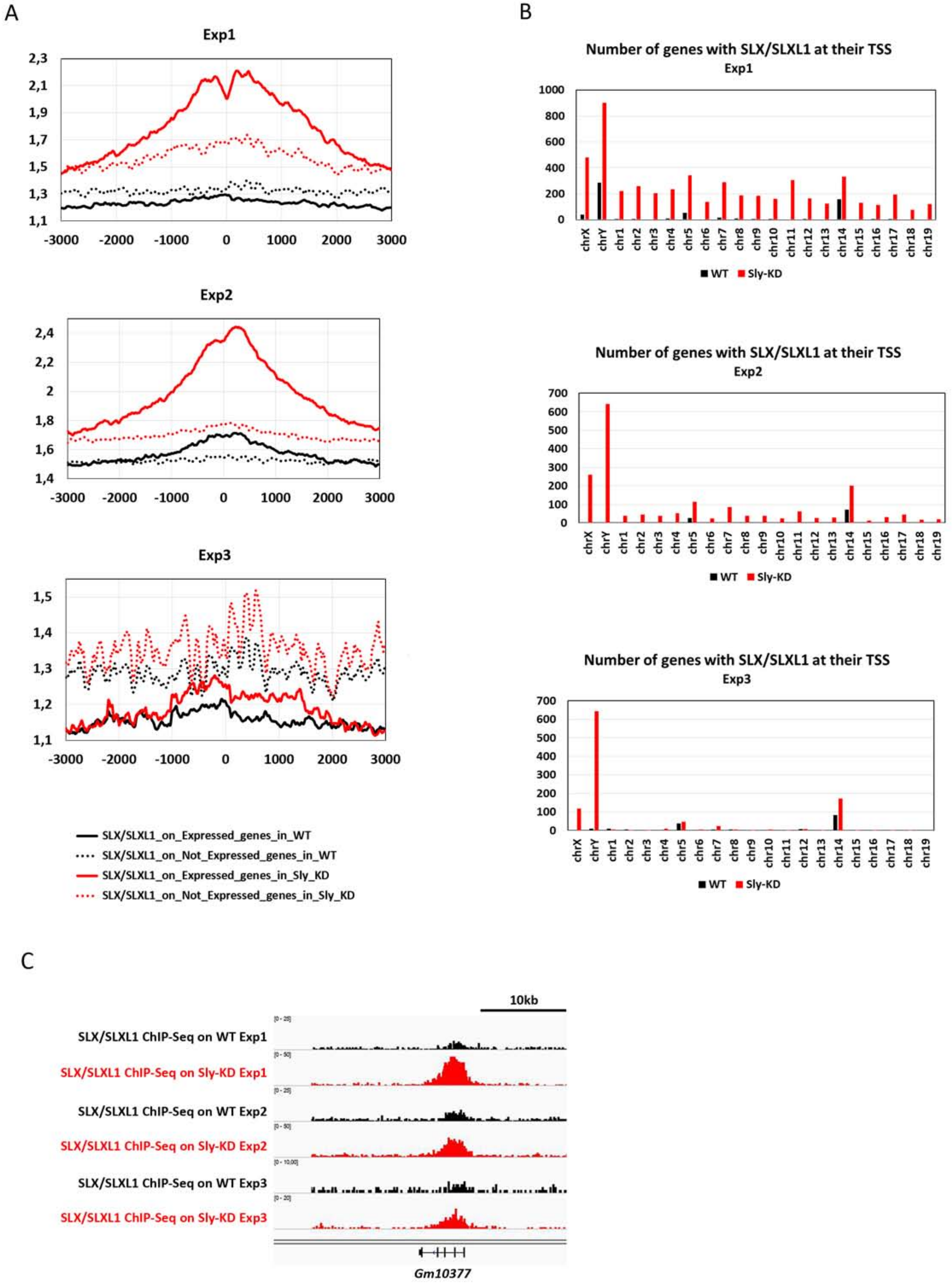
A
Exp1
Exp2
Exp3
-3000
-2000
-1000
0
1000
2000
3000
SLX/SLXL1_on_Expressed_genes_in_WT
SLX/SLXL1_on_Not_Expressed_genes_in_WT
SLX/SLXL1_on_Expressed_genes_in_Sly_KD
SLX/SLXL1_on_Not_Expressed_genes_in_Sly_KD
B
Number of genes with SLX/SLXL1 at their TSS
Exp1
Exp2
Exp3
chrX chrY chr1 chr2 chr3 chr4 chr5 chr6 chr7 chr8 chr9 chr10 chr11 chr12 chr13 chr14 chr15 chr16 chr17 chr18 chr19
WT
Sly-KD
C
10kb
SLX/SLXL1 ChIP-Seq on WT Exp1
SLX/SLXL1 ChIP-Seq on Sly-KD Exp1
SLX/SLXL1 ChIP-Seq on WT Exp2
SLX/SLXL1 ChIP-Seq on Sly-KD Exp2
SLX/SLXL1 ChIP-Seq on WT Exp3
SLX/SLXL1 ChIP-Seq on Sly-KD Exp3
Gm10377

Figure S4

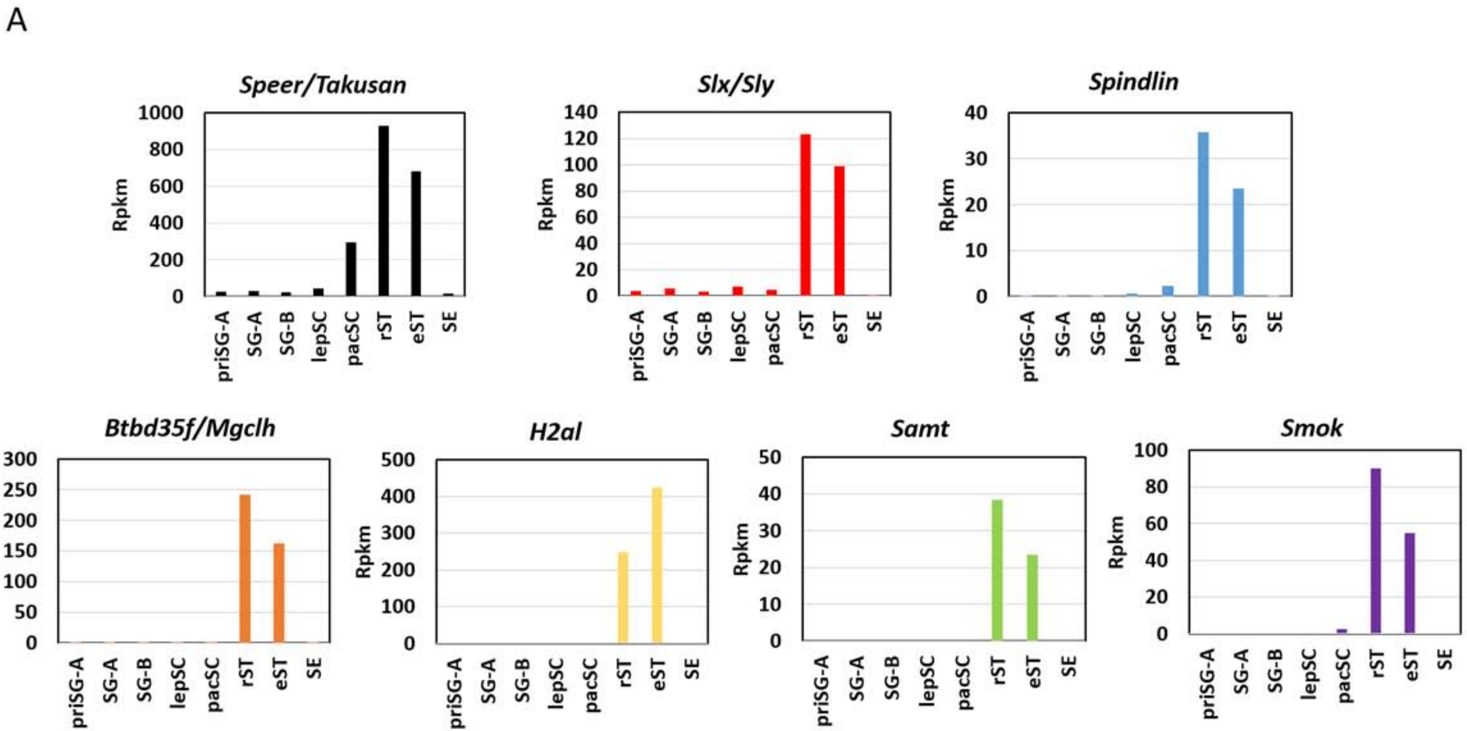
A
Speer/Takusan
Slx/Sly
Spindlin
Btbd35f/Mgclh
H2al
Samt
Smok
Rpkm
priSG-A
SG-A
SG-B
lepSC
pacSC
rST
eST
SE

Figure S5

A

```
SSTY1        ---------------------MSSLMKKRRR------------KSSSNTLRNIVSCRISH
SSTY2        ---------------------MTSLKKKSRR------------KPSSQALGNIVGCRISH
SPIN1        MKTPFGKTPGQRSRADAGHAGVSANMMKKRTSHKKHRTSVGPSKPVSQPRRNIVGCRIQH
                                  :::   * *             *. *:.  ***.***.*

SSTY1        SWKEGNEPVTQWKAIVLDQLPTNPSLYLVKYDGIDSIYVLELYSDDRILNLKVLPPIVVF
SSTY2        GWKEGNEPVTHWKAIILGQLPTNPSLYLVKYDGIDSVYGQELHSDERILNLKVLPHKVDF
SPIN1        GWREGNGPVTQWKGTVLDQVPVNPSLYLIKYDGFDCVYGLELNKDERVSALEVLPDRVAT
             .*:*** ***:**.  :*.*:*.******:****:*.:*  **  .*:*:   *:***   *

SSTY1        PQVRDAHLARALVGRAVQHKFERKDGSEVNWRGVVLAQVPIMKDLFYITYKKDPALYAYQ
SSTY2        PQVREVHLAGTLVGREVQHKFEGKDGSEDNWSGMVLAQVPFLQDYFYISYKKDPVLYVYQ
SPIN1        SRISDAHLADTMIGKAVEHMFETEDGSKDEWRGMVLARAPVMNTWFYITYEKDPVLYMYQ
             .:: :.*** :::*: *:* ** :***: :* *:***:.*.::  ***:*:***.** **

SSTY1        LLDDYKEGNLHMIP---DTPPAEERSGDDSDVLIGNWVEYTRKDGSKKFGKVVYQVLANP
SSTY2        LLDDYKEGNLHIIP---ETPLAEARSGDDNDFLIGSWVQYTRDDGSKKFGKVVYKVLANP
SPIN1        LLDDYKEGDLRIMPDSNDSPPAEREPGEVVDSLVGKQVEYAKEDGSKRTGMVIHQVEAKP
             ********:*:::*   ::* **  ..*:  * *:*. *:*::.****: * *:::* *:*

SSTY1        SVYFIKFHGDIHIYVYTMVPKILEVEKS
SSTY2        TVYFIKFLGDLHIYVYTLVSNIT-----
SPIN1        SVYFIKFDDDFHIYVYDLVKTS------
             :****** .*:***** :* .
```

B

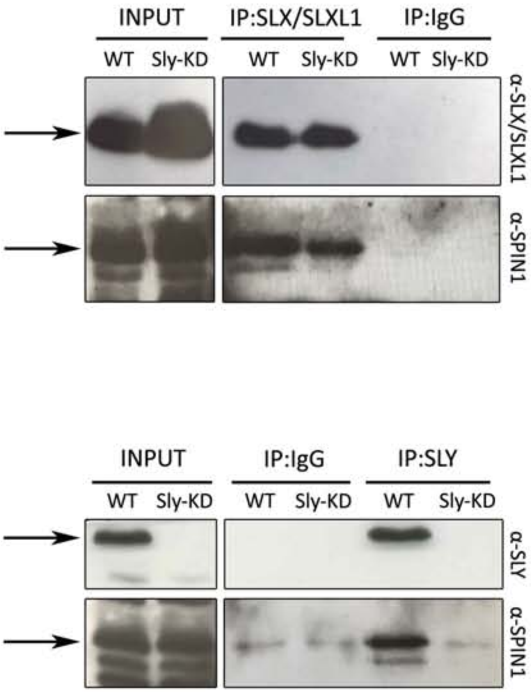

C

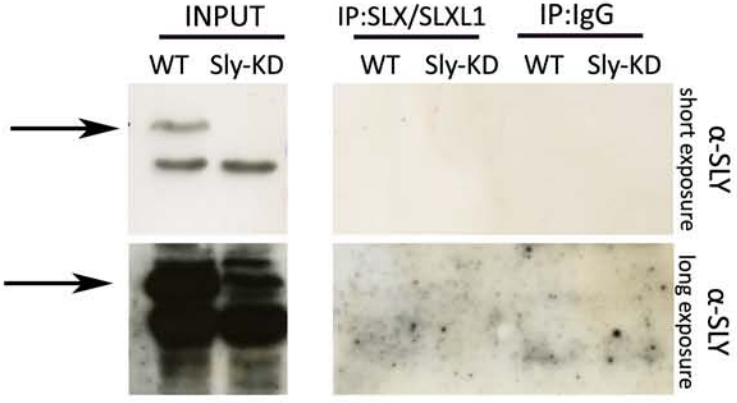

Figure S6

A

Overlap between SSTY1-ChIP-Seq replicates

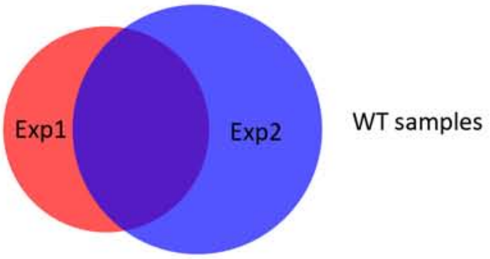

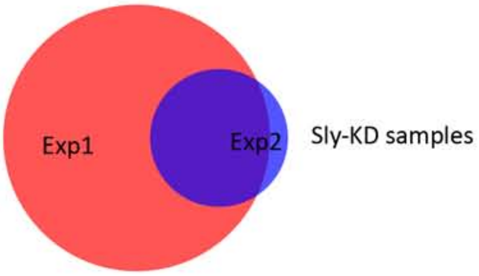

WT replicates	Exp1	Exp2
Exp1	100%	61%
Exp2	41%	100%

Sly-KD replicates	Exp1	Exp2
Exp1	100%	24%
Exp2	90%	100%

B

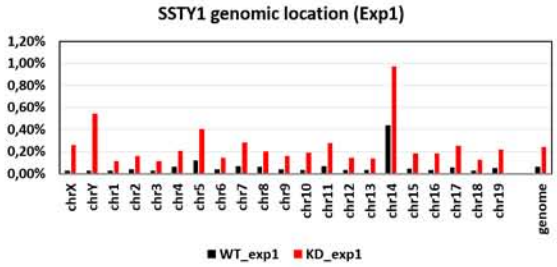

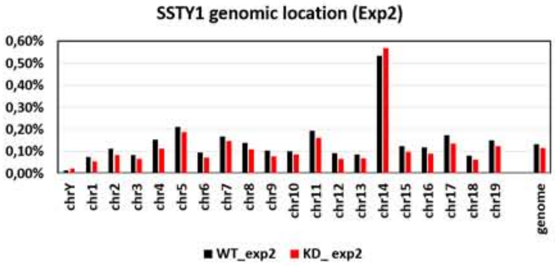

C

SSTY1 genomic location

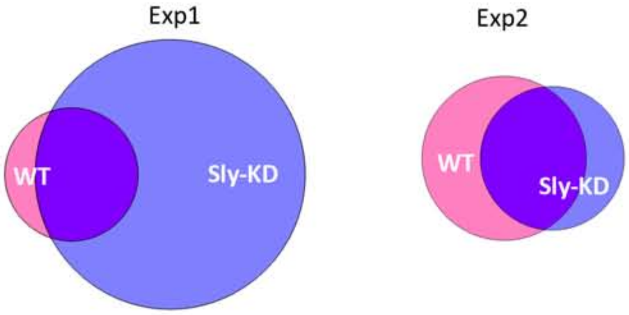

Exp1+2 regions in common

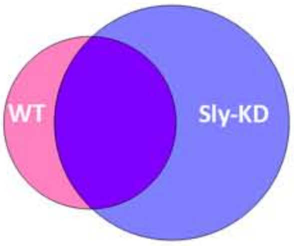

D

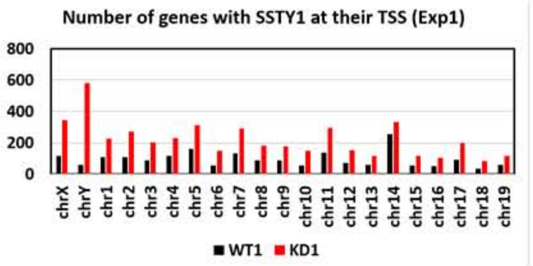

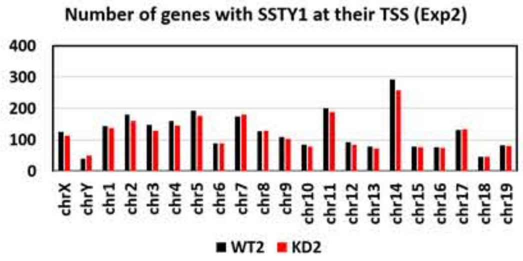

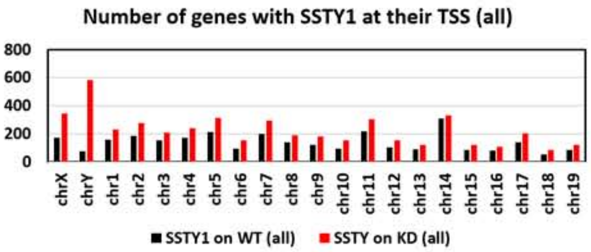

E

SSTY1 target genes
(common in both replicates)

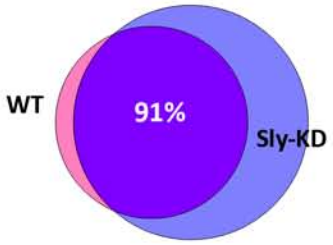

Figure S6

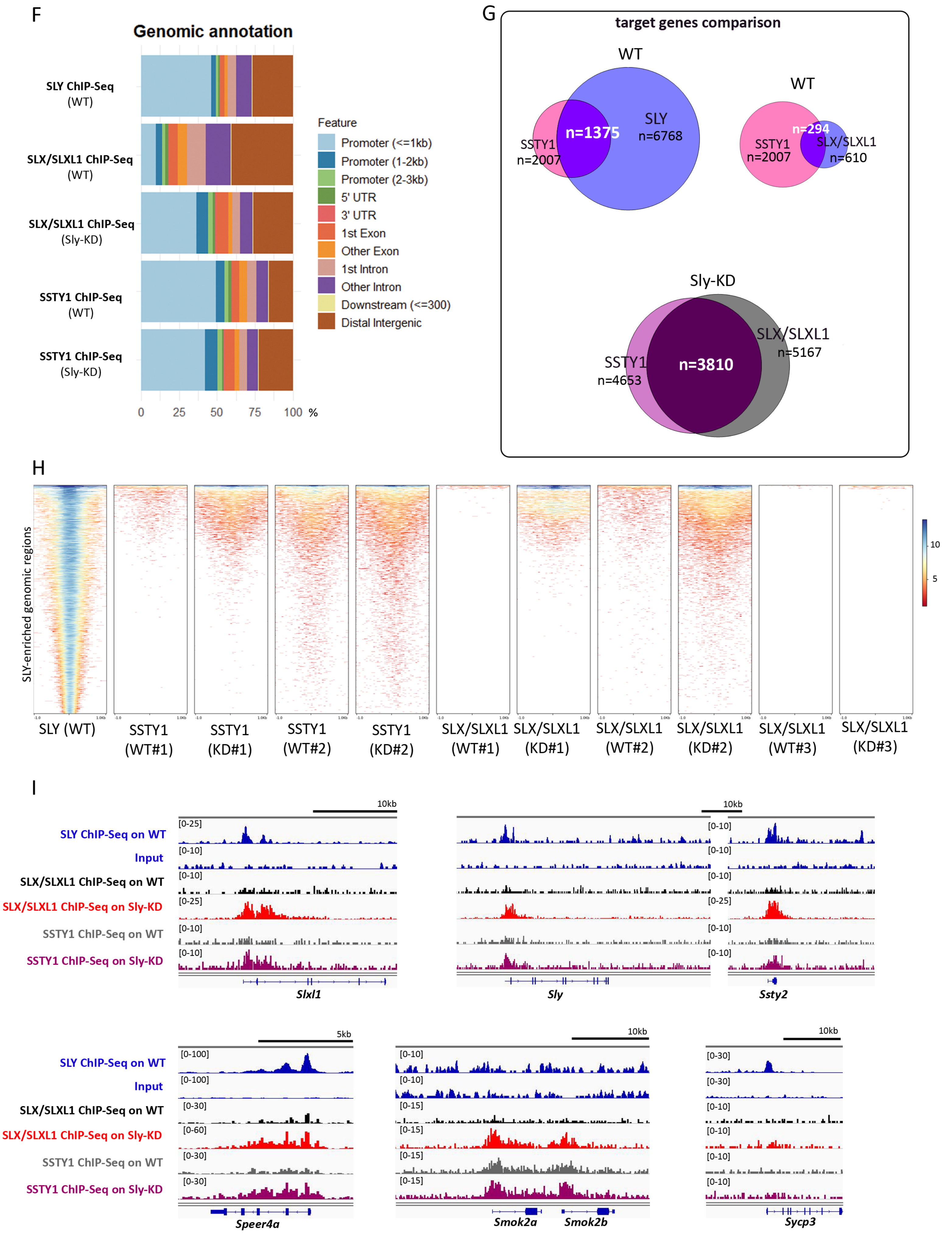
F
Genomic annotation
SLY ChIP-Seq (WT)
SLX/SLXL1 ChIP-Seq (WT)
SLX/SLXL1 ChIP-Seq (Sly-KD)
SSTY1 ChIP-Seq (WT)
SSTY1 ChIP-Seq (Sly-KD)
0 25 50 75 100 %
Feature
Promoter (<=1kb)
Promoter (1-2kb)
Promoter (2-3kb)
5' UTR
3' UTR
1st Exon
Other Exon
1st Intron
Other Intron
Downstream (<=300)
Distal Intergenic
G
target genes comparison
WT
SSTY1 n=2007
n=1375
SLY n=6768
WT
SSTY1 n=2007
n=294
SLX/SLXL1 n=610
Sly-KD
SSTY1 n=4653
n=3810
SLX/SLXL1 n=5167
H
SLY-enriched genomic regions
SLY (WT)
SSTY1 (WT#1)
SSTY1 (KD#1)
SSTY1 (WT#2)
SSTY1 (KD#2)
SLX/SLXL1 (WT#1)
SLX/SLXL1 (KD#1)
SLX/SLXL1 (WT#2)
SLX/SLXL1 (KD#2)
SLX/SLXL1 (WT#3)
SLX/SLXL1 (KD#3)
10
5
I
SLY ChIP-Seq on WT
Input
SLX/SLXL1 ChIP-Seq on WT
SLX/SLXL1 ChIP-Seq on Sly-KD
SSTY1 ChIP-Seq on WT
SSTY1 ChIP-Seq on Sly-KD
10kb
Slxl1
Sly
Ssty2
5kb
Speer4a
Smok2a
Smok2b
Sycp3

Figure S7

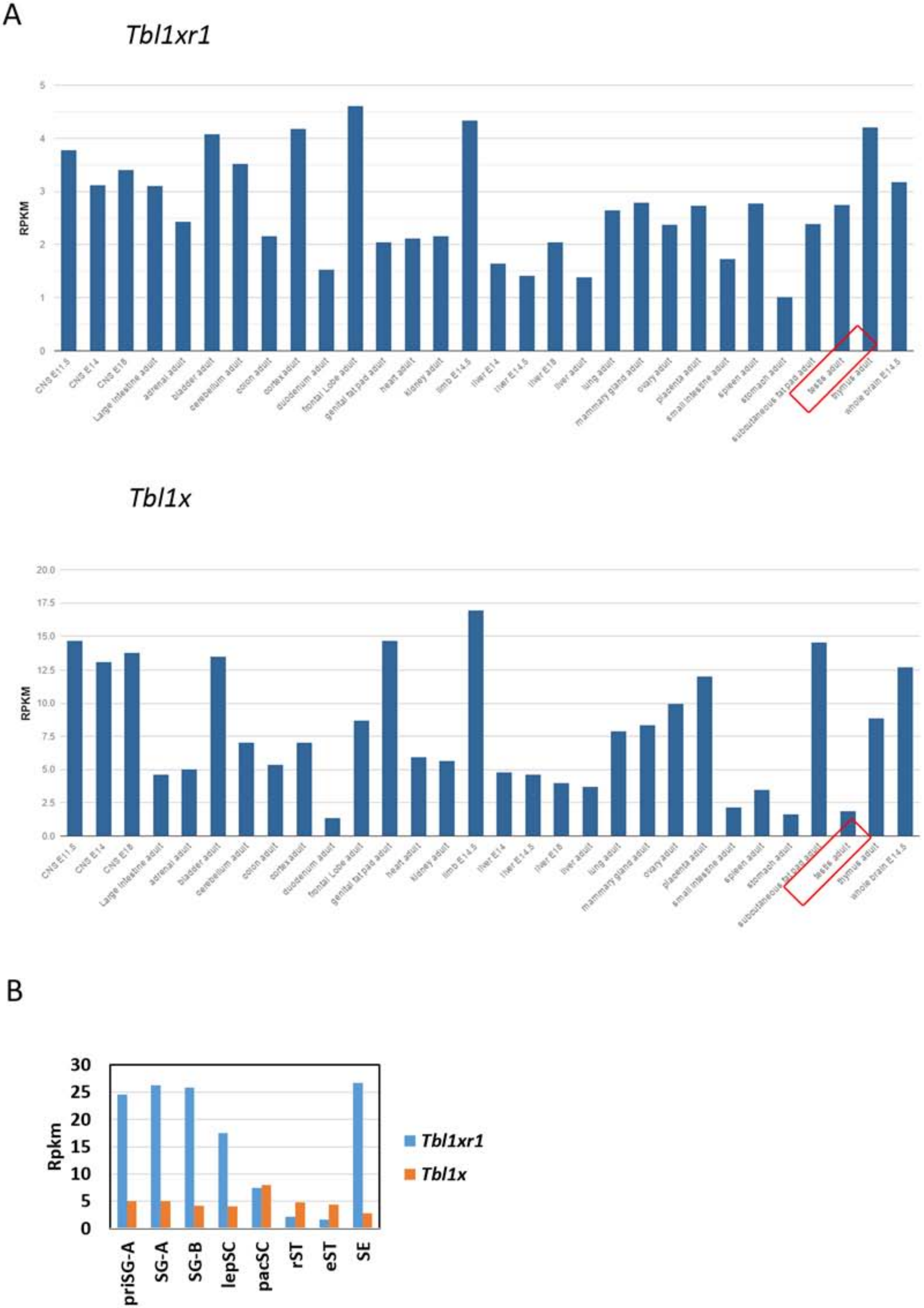
A
Tbl1xr1
RPKM
Tbl1x
RPKM
CNS E11.5
CNS E14
CNS E18
Large intestine adult
adrenal adult
bladder adult
cerebellum adult
colon adult
cortex adult
duodenum adult
frontal Lobe adult
genital fat pad adult
heart adult
kidney adult
limb E14.5
liver E14
liver E14.5
liver E18
liver adult
lung adult
mammary gland adult
ovary adult
placenta adult
small intestine adult
spleen adult
stomach adult
subcutaneous fat pad adult
testis adult
thymus adult
whole brain E14.5
B
Rpkm
Tbl1xr1
Tbl1x
priSG-A
SG-A
SG-B
lepSC
pacSC
rST
eST
SE

C

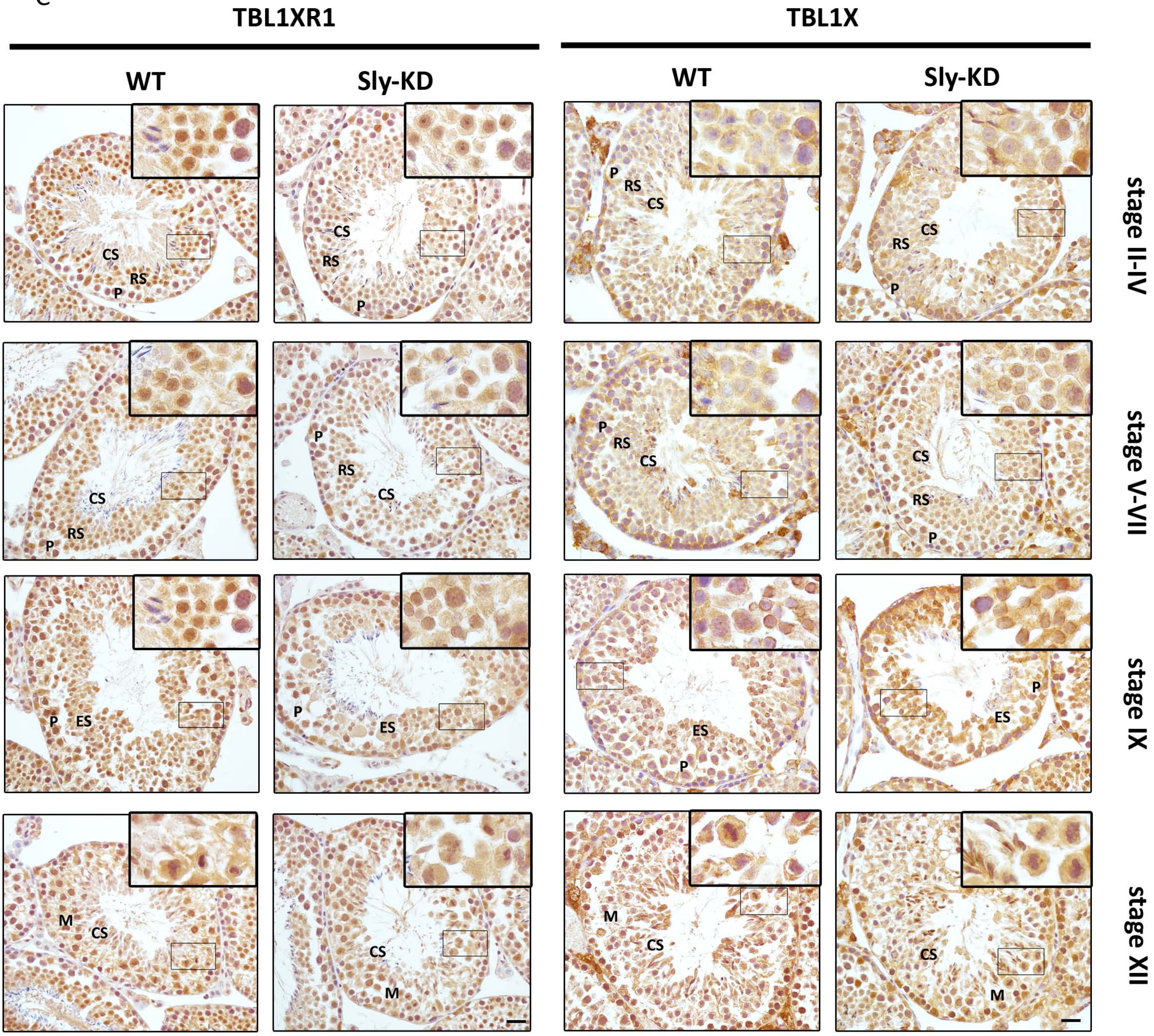
TBL1XR1
TBL1X
WT
Sly-KD
WT
Sly-KD
stage II-IV
stage V-VII
stage IX
stage XII
P
RS
CS
ES
M

D

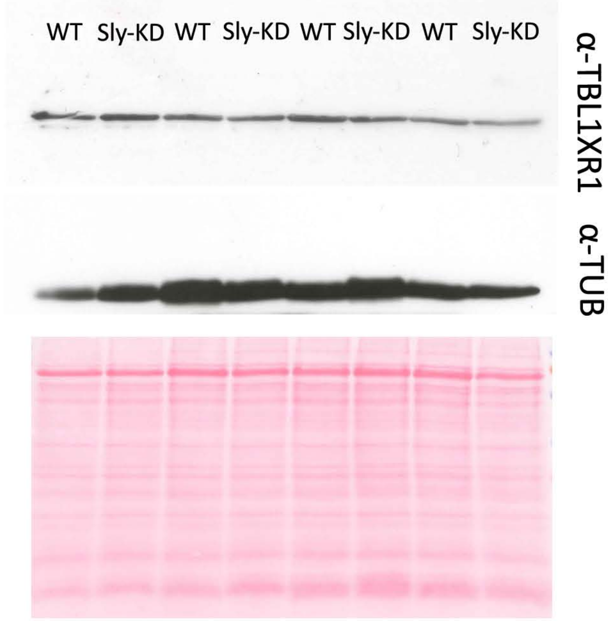
WT
Sly-KD
WT
Sly-KD
WT
Sly-KD
WT
Sly-KD
α-TBL1XR1
α-TUB

Table S1. SLX/SLXL1 ChIP-Seq details (comparison with SLY ChIP-Seq from Moretti et al 2017)

**From Moretti et al 2017*

	SLX/SLXL1 ChIP DNA	SLY ChIP DNA*	Input DNA*
Total number of reads	45 652 544	37 495 530	26 337 788
Total number of alignments	39 648 478	23 699 247	25 636 677
Final number of tags (no duplicate reads)	22 557 157	9 754 313	20 829 154

**same parameters as in Moretti et al. 2017**	**MACS 1.4.2 peak calling**	
	# band width = 200; # model fold = 12,32; # pvalue cutoff = 1.00e-07	
	SLX/SLXL1 ChIP DNA	SLY ChIP DNA*
MACS peaks number	3976	13 664
“Active Regions” (union of overlapping peak intervals)	3912	12 953
Number of genes at +/- 1Kb of TSS (Ensembl80)	1327	7 280

**parameters from current study**	**MACS 1.4.2 peak calling**	
	# band width = 300; # model fold = 10,32; # pvalue cutoff = 1.00e-05	
	SLX/SLXL1 ChIP DNA	SLY ChIP DNA*
MACS peaks number	8150	17 793
Number of genes at +/- 1Kb of TSS (Ensembl80)	2475	6768

Table S2. Summary of the experimental procedures used for 3 independent SLX/SLXL1 ChIP Seq analyses performed in WT and *Sly* -KD spermatids

	**exp#1**	**#exp 2**	**#exp 3**
Technique used to collect round spermatids (RS)	elutriation		
Nb of cells	10 millions of WT RS and 10 millions of Sly-KD RS	30 millions of WT RS and 30 millions of Sly-KD RS	8 millions of WT RS and 8 millions of Sly-KD RS
purity (% of RS)	~84%	~65%	~85%
PFA crosslink	cross link was perform <u>after</u> freezing		cross link was perform <u>before</u> freezing
quantity of antibody	~ 2ug of anti SLX/SLXL1 antibody per 10 millions of cells		

	**exp#1**		
	**ChIP SLX/SLXL1 on WT RS**	**ChIP SLX/SLXL1 on Sly-KD RS**	**WT/Sly-KD Input mix**
Total number of reads	52 691 282	33 131 450	51 312 862
Total number of alignments	34 699 709	21 579 603	50 672 079
Final number of tags (no duplicate reads)	13 864 225	11 496 353	42 881 615
	**MACS 1.4.2 peak calling**		
	# band width = 300; # model fold = 10,32; # pvalue cutoff = 1.00e-05		
MACS peaks number	2956	17929	
Number of genes at +/- 1Kb of TSS (Ensembl80)	610	5167	

exp#2				
	ChIP SLX/SLXL1 on WT RS	ChIP SLX/SLXL1 on Sly-KD RS	WT Input	Sly-KD Input
Total number of reads	48 857 160,00	58 386 413,00	30 875 930,00	32 496 874,00
Total number of alignments	48 151 334,00	57 604 116,00	28 668 549,00	32076756
Final number of tags (no duplicate reads)	38 972 467,00	46 218 186,00	12 194 525,00	26 012 643,00
Normalized				
	**MACS 1.4.2 peak calling**			
	# band width = 300; # model fold = 10,32; # pvalue cutoff = 1.00e-05			
MACS peaks number	255	4996		
Number of genes at +/- 1Kb of TSS (Ensembl80)	122	1859		

exp#3			
	ChIP SLX/SLXL1 on WT RS	ChIP SLX/SLXL1 on Sly-KD RS	WT/Sly-KD Input mix
Total number of reads	34 202 302	32 315 391	42 118 494
Total number of alignments	21 930 293	14 788 162	41 116 113
Final number of tags (no duplicate reads)	9 632 901	5 664 829	14 770 428
Normalized			
	**MACS 1.4.2 peak calling**		
	# band width = 300; # model fold = 10,32; # pvalue cutoff = 1.00e-05		
MACS peaks number	1006	5981	
Number of genes at +/- 1Kb of TSS (Ensembl80)	178	1054	

Table S3. Summary of the experimental procedures used for 2 independent SSTY1 ChIP Seq analyses performed in WT and Sly-KD spermatids

	exp#1 (10/18)	#exp 2 (07/19)
Technique used to collect round spermatids	elutriation	
Nb of cells	5 millions of WT RS and 5 millions of Sly-KD RS	10 millions of WT RS and 10 millions of Sly-KD RS
purity (% of RS)	~87% for WT and ~82% for KD	~70% for WT and KD
PFA crosslink	cross link was performed after freezing	
quantity of antibody	~ 3ug of antibody per 5 millions of cells	~ 8ug of antibody per 10 millions of cells

exp#1

	ChIP SSTY1 on WT RS	ChIP SSTY1 on Sly-KD RS	WT/Sly-KD Input mix
Total number of reads	49 428 018,00	57 596 807,00	48 437 133,00
Total number of alignments	30 709 696	39 845 973	47 866 899
Final number of tags (no duplicate reads)	22 456 198	27 461 015	37 491 556
Redundant rate	0,27	0,31	0,22
**MACS 1.4.2 peak calling**			
# band width = 300; # model fold = 10,32; # pvalue cutoff = 1.00e-05			
Nb of Peaks (mapped)	5777	16603	
Number of genes at +/- 1Kb of TSS	2007	4653	

exp#2

	ChIP SSTY1 on WT RS	ChIP SSTY1 on Sly-KD RS	WT/Sly-KD Input mix
Total number of reads	63268055	67 980 616	54 442 650,00
Total number of alignments	61 672 975,00	66 909 545,00	53 836 132
Final number of tags (no duplicate reads)	44 621 924	50 398 252	40 801 302
Redundant rate	0,28	0,25	0,24
**MACS 1.4.2 peak calling**			
# band width = 300; # model fold = 10,32; # pvalue cutoff = 1.00e-05			
Nb of Peaks (mapped)	6513	5240	
Number of genes at +/- 1Kb of TSS	2659	2512	

Table S4. Common target genes (TSS +/-1Kb) between SLY, SLX/SLXL1 and SSTY1 (all target genes found in WT and *Sly*-KD round spermatids)

**ENSMUSG00000092165**	chr14	ENSMUSG00000041028	chr14	ENSMUSG00000096527	chr5
**ENSMUSG00000090713**	chr14	ENSMUSG00000021811	chr14	ENSMUSG00000096798	chr5
**ENSMUSG00000096793**	chr14	ENSMUSG00000044186	chr14	ENSMUSG00000041505	chr5
**ENSMUSG00000096685**	chr14	ENSMUSG00000043881	chr14	ENSMUSG00000029620	chr5
**ENSMUSG00000091569**	chr14	ENSMUSG00000075502	chr14	ENSMUSG00000061707	chr5
**ENSMUSG00000091400**	chr14	ENSMUSG00000035067	chr14	ENSMUSG00000048520	chr5
**ENSMUSG00000093985**	chr14	ENSMUSG00000072680	chr14	ENSMUSG00000093445	chr5
**ENSMUSG00000091584**	chr14	ENSMUSG00000021867	chr14	ENSMUSG00000029720	chr5
**ENSMUSG00000095797**	chr14	ENSMUSG00000021961	chr14	ENSMUSG00000070639	chr5
**ENSMUSG00000094021**	chr14	ENSMUSG00000091980	chr14	ENSMUSG00000053293	chr5
**ENSMUSG00000094370**	chr14	ENSMUSG00000038792	chr14	ENSMUSG00000040731	chr5
**ENSMUSG00000093979**	chr14	ENSMUSG00000103520	chr15	ENSMUSG00000000568	chr5
**ENSMUSG00000071613**	chr14	ENSMUSG00000091050	chr15	ENSMUSG00000029599	chr5
**ENSMUSG00000090690**	chr14	ENSMUSG00000097259	chr15	ENSMUSG00000029203	chr5
**ENSMUSG00000090594**	chr14	ENSMUSG00000049687	chr15	ENSMUSG00000063447	chr5
**ENSMUSG00000095384**	chr14	ENSMUSG00000087331	chr15	ENSMUSG00000029128	chr5
**ENSMUSG00000090627**	chr14	ENSMUSG00000022255	chr15	ENSMUSG00000029131	chr5
**ENSMUSG00000079389**	chr14	ENSMUSG00000089647	chr15	ENSMUSG00000001467	chr5
**ENSMUSG00000079380**	chr14	ENSMUSG00000056851	chr15	ENSMUSG00000079215	chr5
**ENSMUSG00000090872**	chr14	ENSMUSG00000098625	chr15	ENSMUSG00000043059	chr5
**ENSMUSG00000079271**	chr14	ENSMUSG00000055024	chr15	ENSMUSG00000028986	chr5
**ENSMUSG00000091114**	chr14	ENSMUSG00000022200	chr15	ENSMUSG00000029640	chr5
**ENSMUSG00000090440**	chr14	ENSMUSG00000035845	chr15	ENSMUSG00000037822	chr5
**ENSMUSG00000094954**	chr14	ENSMUSG00000063480	chr15	ENSMUSG00000029660	chr5
**ENSMUSG00000091733**	chr14	ENSMUSG00000097232	chr15	ENSMUSG00000029535	chr5
**ENSMUSG00000095533**	chr14	ENSMUSG00000022564	chr15	ENSMUSG00000089734	chr5
**ENSMUSG00000091022**	chr14	ENSMUSG00000097221	chr15	ENSMUSG00000053656	chr5
**ENSMUSG00000091477**	chr14	ENSMUSG00000080527	chr15	ENSMUSG00000045709	chr6
**ENSMUSG00000094349**	chr14	ENSMUSG00000067547	chr15	ENSMUSG00000030335	chr6
**ENSMUSG00000095056**	chr14	ENSMUSG00000022401	chr15	ENSMUSG00000004980	chr6
**ENSMUSG00000095912**	chr14	ENSMUSG00000039458	chr15	ENSMUSG00000099354	chr6
**ENSMUSG00000095368**	chr14	ENSMUSG00000037627	chr15	ENSMUSG00000101404	chr6
**ENSMUSG00000091429**	chr14	ENSMUSG00000022201	chr15	ENSMUSG00000038836	chr6
**ENSMUSG00000090487**	chr14	ENSMUSG00000022365	chr15	ENSMUSG00000058706	chr6
**ENSMUSG00000090740**	chr14	ENSMUSG00000048385	chr15	ENSMUSG00000004270	chr6
**ENSMUSG00000095360**	chr14	ENSMUSG00000022453	chr15	ENSMUSG00000084950	chr6
**ENSMUSG00000091142**	chr14	ENSMUSG00000037458	chr15	ENSMUSG00000089781	chr6
**ENSMUSG00000090827**	chr14	ENSMUSG00000043091	chr15	ENSMUSG00000023089	chr6
**ENSMUSG00000079371**	chr14	ENSMUSG00000022280	chr15	ENSMUSG00000048794	chr6
**ENSMUSG00000095113**	chr14	ENSMUSG00000068206	chr15	ENSMUSG00000098762	chr6
**ENSMUSG00000079386**	chr14	ENSMUSG00000022388	chr15	ENSMUSG00000041791	chr6
**ENSMUSG00000094543**	chr14	ENSMUSG00000037075	chr15	ENSMUSG00000029833	chr6
**ENSMUSG00000094784**	chr14	ENSMUSG00000037646	chr15	ENSMUSG00000097048	chr6
**ENSMUSG00000090634**	chr14	ENSMUSG00000022999	chr15	ENSMUSG00000099662	chr6
**ENSMUSG00000094634**	chr14	ENSMUSG00000042406	chr15	ENSMUSG00000047515	chr6
**ENSMUSG00000092152**	chr14	ENSMUSG00000016619	chr15	ENSMUSG00000038252	chr6
**ENSMUSG00000095226**	chr14	ENSMUSG00000016541	chr15	ENSMUSG00000084772	chr6
**ENSMUSG00000090505**	chr14	ENSMUSG00000022426	chr15	ENSMUSG00000030216	chr6

**ENSMUSG00000093945**	chr14	ENSMUSG00000022451	chr15	ENSMUSG00000084861	chr6
**ENSMUSG00000091296**	chr14	ENSMUSG00000022431	chr15	ENSMUSG00000106591	chr6
**ENSMUSG00000096904**	chr14	ENSMUSG00000022236	chr15	ENSMUSG00000097675	chr6
**ENSMUSG00000096003**	chr14	ENSMUSG00000022412	chr15	ENSMUSG00000042087	chr6
**ENSMUSG00000063277**	chr14	ENSMUSG00000061923	chr15	ENSMUSG00000052241	chr6
**ENSMUSG00000093926**	chr14	ENSMUSG00000022285	chr15	ENSMUSG00000062995	chr6
**ENSMUSG00000091140**	chr14	ENSMUSG00000022432	chr15	ENSMUSG00000005069	chr6
**ENSMUSG00000091227**	chr14	ENSMUSG00000062397	chr15	ENSMUSG00000051695	chr6
**ENSMUSG00000090512**	chr14	ENSMUSG00000022487	chr15	ENSMUSG00000030230	chr6
**ENSMUSG00000091494**	chr14	ENSMUSG00000022428	chr15	ENSMUSG00000034245	chr6
**ENSMUSG00000079409**	chr14	ENSMUSG00000022403	chr15	ENSMUSG00000042213	chr6
**ENSMUSG00000096024**	chr14	ENSMUSG00000042535	chr15	ENSMUSG00000030206	chr6
**ENSMUSG00000079396**	chr14	ENSMUSG00000054277	chr15	ENSMUSG00000063870	chr6
**ENSMUSG00000091568**	chr14	ENSMUSG00000023010	chr15	ENSMUSG00000029992	chr6
**ENSMUSG00000094132**	chr14	ENSMUSG00000022283	chr15	ENSMUSG00000030161	chr6
**ENSMUSG00000094811**	chr14	ENSMUSG00000002524	chr15	ENSMUSG00000030304	chr6
**ENSMUSG00000091676**	chr14	ENSMUSG00000055065	chr15	ENSMUSG00000052738	chr6
**ENSMUSG00000093898**	chr14	ENSMUSG00000022621	chr15	ENSMUSG00000044927	chr6
**ENSMUSG00000095318**	chr14	ENSMUSG00000023034	chr15	ENSMUSG00000020440	chr6
**ENSMUSG00000095490**	chr14	ENSMUSG00000022391	chr15	ENSMUSG00000030104	chr6
**ENSMUSG00000091122**	chr14	ENSMUSG00000022246	chr15	ENSMUSG00000029686	chr6
**ENSMUSG00000091725**	chr14	ENSMUSG00000033055	chr15	ENSMUSG00000038451	chr6
**ENSMUSG00000095518**	chr14	ENSMUSG00000022622	chr15	ENSMUSG00000029823	chr6
**ENSMUSG00000096372**	chr14	ENSMUSG00000023272	chr15	ENSMUSG00000053470	chr6
**ENSMUSG00000072595**	chr14	ENSMUSG00000029875	chr15	ENSMUSG00000030323	chr6
**ENSMUSG00000095195**	chr14	ENSMUSG00000022452	chr15	ENSMUSG00000030327	chr6
**ENSMUSG00000090472**	chr14	ENSMUSG00000042564	chr15	ENSMUSG00000045896	chr6
**ENSMUSG00000090691**	chr14	ENSMUSG00000078938	chr15	ENSMUSG00000030291	chr6
**ENSMUSG00000096901**	chr14	ENSMUSG00000097058	chr15	ENSMUSG00000004285	chr6
**ENSMUSG00000090363**	chr14	ENSMUSG00000022790	chr16	ENSMUSG00000030322	chr6
**ENSMUSG00000092148**	chr14	ENSMUSG00000086550	chr16	ENSMUSG00000033933	chr6
**ENSMUSG00000091754**	chr14	ENSMUSG00000034243	chr16	ENSMUSG00000029817	chr6
**ENSMUSG00000091563**	chr14	ENSMUSG00000099968	chr16	ENSMUSG00000040234	chr6
**ENSMUSG00000079383**	chr14	ENSMUSG00000102101	chr16	ENSMUSG00000038538	chr6
**ENSMUSG00000094460**	chr14	ENSMUSG00000088544	chr16	ENSMUSG00000052144	chr6
**ENSMUSG00000079388**	chr14	ENSMUSG00000014074	chr16	ENSMUSG00000029802	chr6
**ENSMUSG00000096197**	chr14	ENSMUSG00000101234	chr16	ENSMUSG00000023456	chr6
**ENSMUSG00000091792**	chr14	ENSMUSG00000091416	chr16	ENSMUSG00000072770	chr6
**ENSMUSG00000091700**	chr14	ENSMUSG00000022838	chr16	ENSMUSG00000063810	chr6
**ENSMUSG00000079269**	chr14	ENSMUSG00000085398	chr16	ENSMUSG00000030275	chr6
**ENSMUSG00000094925**	chr14	ENSMUSG00000088524	chr16	ENSMUSG00000038279	chr6
**ENSMUSG00000094715**	chr14	ENSMUSG00000053774	chr16	ENSMUSG00000029836	chr6
**ENSMUSG00000090643**	chr14	ENSMUSG00000022982	chr16	ENSMUSG00000030032	chr6
**ENSMUSG00000092142**	chr14	ENSMUSG00000022962	chr16	ENSMUSG00000030057	chr6
**ENSMUSG00000094258**	chr14	ENSMUSG00000005615	chr16	ENSMUSG00000056429	chr6
**ENSMUSG00000090547**	chr14	ENSMUSG00000005981	chr16	ENSMUSG00000002413	chr6
**ENSMUSG00000095371**	chr14	ENSMUSG00000022911	chr16	ENSMUSG00000039159	chr6
**ENSMUSG00000094804**	chr14	ENSMUSG00000022722	chr16	ENSMUSG00000014747	chr6
**ENSMUSG00000068506**	chr14	ENSMUSG00000022683	chr16	ENSMUSG00000029685	chr6
**ENSMUSG00000095551**	chr14	ENSMUSG00000022974	chr16	ENSMUSG00000029552	chr6

**ENSMUSG00000096023**	chr14	ENSMUSG00000052299	chr16	ENSMUSG00000029754	chr6
**ENSMUSG00000094929**	chr14	ENSMUSG00000047714	chr16	ENSMUSG00000033963	chr6
**ENSMUSG00000072605**	chr14	ENSMUSG00000022855	chr16	ENSMUSG00000029828	chr6
**ENSMUSG00000095686**	chr14	ENSMUSG00000022961	chr16	ENSMUSG00000004535	chr6
**ENSMUSG00000072726**	chr14	ENSMUSG00000022972	chr16	ENSMUSG00000030030	chr6
**ENSMUSG00000091882**	chr14	ENSMUSG00000022773	chr16	ENSMUSG00000033940	chr6
**ENSMUSG00000091756**	chr14	ENSMUSG00000050058	chr16	ENSMUSG00000068250	chr6
**ENSMUSG00000079378**	chr14	ENSMUSG00000005982	chr16	ENSMUSG00000005566	chr7
**ENSMUSG00000079402**	chr14	ENSMUSG00000092470	chr16	ENSMUSG00000073856	chr7
**ENSMUSG00000090379**	chr14	ENSMUSG00000032965	chr16	ENSMUSG00000101548	chr7
**ENSMUSG00000091148**	chr14	ENSMUSG00000022914	chr16	ENSMUSG00000094419	chr7
**ENSMUSG00000096446**	chr14	ENSMUSG00000071533	chr16	ENSMUSG00000030788	chr7
**ENSMUSG00000095466**	chr14	ENSMUSG00000006134	chr16	ENSMUSG00000015149	chr7
**ENSMUSG00000095015**	chr14	ENSMUSG00000039738	chr16	ENSMUSG00000055159	chr7
**ENSMUSG00000095024**	chr14	ENSMUSG00000052363	chr16	ENSMUSG00000085039	chr7
**ENSMUSG00000096183**	chr14	ENSMUSG00000062203	chr16	ENSMUSG00000030990	chr7
**ENSMUSG00000091740**	chr14	ENSMUSG00000022656	chr16	ENSMUSG00000035632	chr7
**ENSMUSG00000096470**	chr14	ENSMUSG00000004071	chr16	ENSMUSG00000035623	chr7
**ENSMUSG00000091131**	chr14	ENSMUSG00000022867	chr16	ENSMUSG00000074903	chr7
**ENSMUSG00000095743**	chr14	ENSMUSG00000022748	chr16	ENSMUSG00000102017	chr7
**ENSMUSG00000091110**	chr14	ENSMUSG00000022884	chr16	ENSMUSG00000061119	chr7
**ENSMUSG00000093833**	chr14	ENSMUSG00000022674	chr16	ENSMUSG00000099981	chr7
**ENSMUSG00000091718**	chr14	ENSMUSG00000022515	chr16	ENSMUSG00000097595	chr7
**ENSMUSG00000091472**	chr14	ENSMUSG00000045521	chr16	ENSMUSG00000100721	chr7
**ENSMUSG00000093968**	chr14	ENSMUSG00000022601	chr16	ENSMUSG00000094365	chr7
**ENSMUSG00000091923**	chr14	ENSMUSG00000022768	chr16	ENSMUSG00000106236	chr7
**ENSMUSG00000091657**	chr14	ENSMUSG00000055692	chr16	ENSMUSG00000099765	chr7
**ENSMUSG00000090404**	chr14	ENSMUSG00000022915	chr16	ENSMUSG00000094920	chr7
**ENSMUSG00000093863**	chr14	ENSMUSG00000040356	chr17	ENSMUSG00000097075	chr7
**ENSMUSG00000094151**	chr14	ENSMUSG00000085521	chr17	ENSMUSG00000001829	chr7
**ENSMUSG00000094706**	chr14	ENSMUSG00000097103	chr17	ENSMUSG00000049676	chr7
**ENSMUSG00000095044**	chr14	ENSMUSG00000090273	chr17	ENSMUSG00000090457	chr7
**ENSMUSG00000091698**	chr14	ENSMUSG00000084751	chr17	ENSMUSG00000078737	chr7
**ENSMUSG00000090539**	chr14	ENSMUSG00000090952	chr17	ENSMUSG00000074141	chr7
**ENSMUSG00000091185**	chr14	ENSMUSG00000097047	chr17	ENSMUSG00000064767	chr7
**ENSMUSG00000094578**	chr14	ENSMUSG00000097125	chr17	ENSMUSG00000097842	chr7
**ENSMUSG00000096869**	chr14	ENSMUSG00000094843	chr17	ENSMUSG00000085886	chr7
**ENSMUSG00000090764**	chr14	ENSMUSG00000080316	chr17	ENSMUSG00000087376	chr7
**ENSMUSG00000095710**	chr5	ENSMUSG00000093514	chr17	ENSMUSG00000061099	chr7
**ENSMUSG00000067848**	chr5	ENSMUSG00000019578	chr17	ENSMUSG00000042178	chr7
**ENSMUSG00000106445**	chr5	ENSMUSG00000071054	chr17	ENSMUSG00000097170	chr7
**ENSMUSG00000099762**	chr5	ENSMUSG00000090069	chr17	ENSMUSG00000092381	chr7
**ENSMUSG00000096846**	chr5	ENSMUSG00000071172	chr17	ENSMUSG00000093238	chr7
**ENSMUSG00000072188**	chr5	ENSMUSG00000044477	chr17	ENSMUSG00000093599	chr7
**ENSMUSG00000096732**	chr5	ENSMUSG00000039220	chr17	ENSMUSG00000066175	chr7
**ENSMUSG00000094205**	chr5	ENSMUSG00000090101	chr17	ENSMUSG00000097527	chr7
**ENSMUSG00000091049**	chr5	ENSMUSG00000065479	chr17	ENSMUSG00000100841	chr7
**ENSMUSG00000051940**	chr5	ENSMUSG00000077709	chr17	ENSMUSG00000065016	chr7
**ENSMUSG00000091255**	chr5	ENSMUSG00000023952	chr17	ENSMUSG00000101360	chr7
**ENSMUSG00000091827**	chr5	ENSMUSG00000105758	chr17	ENSMUSG00000101855	chr7

**ENSMUSG00000096878**	chr5	ENSMUSG00000099474	chr17	ENSMUSG00000085725	chr7
**ENSMUSG00000069720**	chr5	ENSMUSG00000095869	chr17	ENSMUSG00000054499	chr7
**ENSMUSG00000094025**	chr5	ENSMUSG00000095677	chr17	ENSMUSG00000103887	chr7
**ENSMUSG00000095550**	chr5	ENSMUSG00000092074	chr17	ENSMUSG00000097162	chr7
**ENSMUSG00000095829**	chr5	ENSMUSG00000021545	chr17	ENSMUSG00000030714	chr7
**ENSMUSG00000059645**	chr5	ENSMUSG00000092799	chr17	ENSMUSG00000033961	chr7
**ENSMUSG00000073117**	chr5	ENSMUSG00000054957	chr17	ENSMUSG00000030722	chr7
**ENSMUSG00000073208**	chr5	ENSMUSG00000065396	chr17	ENSMUSG00000003269	chr7
**ENSMUSG00000091903**	chr5	ENSMUSG00000093118	chr17	ENSMUSG00000038973	chr7
**ENSMUSG00000033219**	chr5	ENSMUSG00000089255	chr17	ENSMUSG00000051618	chr7
**ENSMUSG00000069355**	chr5	ENSMUSG00000057863	chr17	ENSMUSG00000003429	chr7
**ENSMUSG00000094036**	chr5	ENSMUSG00000034254	chr17	ENSMUSG00000039202	chr7
**ENSMUSG00000058643**	chr5	ENSMUSG00000024097	chr17	ENSMUSG00000002984	chr7
**ENSMUSG00000104824**	chr5	ENSMUSG00000003200	chr17	ENSMUSG00000030655	chr7
**ENSMUSG00000048703**	chr5	ENSMUSG00000033826	chr17	ENSMUSG00000037239	chr7
**ENSMUSG00000091897**	chr5	ENSMUSG00000036315	chr17	ENSMUSG00000060791	chr7
**ENSMUSG00000067698**	chr5	ENSMUSG00000024095	chr17	ENSMUSG00000025584	chr7
**ENSMUSG00000106627**	chr5	ENSMUSG00000024146	chr17	ENSMUSG00000036427	chr7
**ENSMUSG00000073119**	chr5	ENSMUSG00000047407	chr17	ENSMUSG00000070568	chr7
**ENSMUSG00000096045**	chr5	ENSMUSG00000092519	chr17	ENSMUSG00000000605	chr7
**ENSMUSG00000094105**	chr5	ENSMUSG00000015605	chr17	ENSMUSG00000046364	chr7
**ENSMUSG00000095918**	chr5	ENSMUSG00000007029	chr17	ENSMUSG00000040364	chr7
**ENSMUSG00000095296**	chr5	ENSMUSG00000002625	chr17	ENSMUSG00000045467	chr7
**ENSMUSG00000070933**	chr5	ENSMUSG00000024160	chr17	ENSMUSG00000048787	chr7
**ENSMUSG00000091933**	chr5	ENSMUSG00000015478	chr17	ENSMUSG00000030407	chr7
**ENSMUSG00000067700**	chr5	ENSMUSG00000024426	chr17	ENSMUSG00000030747	chr7
**ENSMUSG00000073116**	chr5	ENSMUSG00000040482	chr17	ENSMUSG00000011349	chr7
ENSMUSG00000085526	chr1	ENSMUSG00000040771	chr17	ENSMUSG00000030591	chr7
ENSMUSG00000055833	chr1	ENSMUSG00000024193	chr17	ENSMUSG00000002968	chr7
ENSMUSG00000053286	chr1	ENSMUSG00000023994	chr17	ENSMUSG00000038502	chr7
ENSMUSG00000026171	chr1	ENSMUSG00000040888	chr17	ENSMUSG00000035545	chr7
ENSMUSG00000073486	chr1	ENSMUSG00000062859	chr17	ENSMUSG00000052296	chr7
ENSMUSG00000100954	chr1	ENSMUSG00000002658	chr17	ENSMUSG00000040824	chr7
ENSMUSG00000097744	chr1	ENSMUSG00000024436	chr17	ENSMUSG00000032637	chr7
ENSMUSG00000086350	chr1	ENSMUSG00000002767	chr17	ENSMUSG00000038539	chr7
ENSMUSG00000026568	chr1	ENSMUSG00000039770	chr17	ENSMUSG00000038695	chr7
ENSMUSG00000053153	chr1	ENSMUSG00000024180	chr17	ENSMUSG00000040811	chr7
ENSMUSG00000104111	chr1	ENSMUSG00000024251	chr17	ENSMUSG00000006024	chr7
ENSMUSG00000102408	chr1	ENSMUSG00000024145	chr17	ENSMUSG00000005609	chr7
ENSMUSG00000026034	chr1	ENSMUSG00000024392	chr17	ENSMUSG00000030718	chr7
ENSMUSG00000039630	chr1	ENSMUSG00000061207	chr17	ENSMUSG00000030922	chr7
ENSMUSG00000102425	chr1	ENSMUSG00000069135	chr17	ENSMUSG00000059891	chr7
ENSMUSG00000044854	chr1	ENSMUSG00000036568	chr17	ENSMUSG00000046750	chr7
ENSMUSG00000026353	chr1	ENSMUSG00000042625	chr17	ENSMUSG00000038618	chr7
ENSMUSG00000041763	chr1	ENSMUSG00000019320	chr17	ENSMUSG00000030682	chr7
ENSMUSG00000070489	chr1	ENSMUSG00000024223	chr17	ENSMUSG00000042606	chr7
ENSMUSG00000104312	chr1	ENSMUSG00000002768	chr17	ENSMUSG00000036882	chr7
ENSMUSG00000025977	chr1	ENSMUSG00000024369	chr17	ENSMUSG00000035642	chr7
ENSMUSG00000102231	chr1	ENSMUSG00000035051	chr17	ENSMUSG00000078794	chr7
ENSMUSG00000026113	chr1	ENSMUSG00000043445	chr17	ENSMUSG00000041328	chr7

ENSMUSG00000043716	chr1	ENSMUSG00000007033	chr17	ENSMUSG00000062732	chr7
ENSMUSG00000101503	chr1	ENSMUSG00000091971	chr17	ENSMUSG00000004371	chr7
ENSMUSG00000100850	chr1	ENSMUSG00000003546	chr17	ENSMUSG00000030878	chr7
ENSMUSG00000039210	chr1	ENSMUSG00000096255	chr17	ENSMUSG00000063802	chr7
ENSMUSG00000026274	chr1	ENSMUSG00000000579	chr17	ENSMUSG00000043831	chr7
ENSMUSG00000073485	chr1	ENSMUSG00000023806	chr17	ENSMUSG00000047248	chr7
ENSMUSG00000102865	chr1	ENSMUSG00000073471	chr17	ENSMUSG00000000131	chr7
ENSMUSG00000026200	chr1	ENSMUSG00000023971	chr17	ENSMUSG00000043432	chr7
ENSMUSG00000092705	chr1	ENSMUSG00000044526	chr17	ENSMUSG00000025482	chr7
ENSMUSG00000026495	chr1	ENSMUSG00000024014	chr17	ENSMUSG00000038187	chr7
ENSMUSG00000099811	chr1	ENSMUSG00000036438	chr17	ENSMUSG00000064307	chr7
ENSMUSG00000102457	chr1	ENSMUSG00000045257	chr17	ENSMUSG00000044465	chr7
ENSMUSG00000087213	chr1	ENSMUSG00000052031	chr17	ENSMUSG00000030689	chr7
ENSMUSG00000090394	chr1	ENSMUSG00000063576	chr17	ENSMUSG00000030980	chr7
ENSMUSG00000102833	chr1	ENSMUSG00000039183	chr17	ENSMUSG00000092518	chr7
ENSMUSG00000087561	chr1	ENSMUSG00000048602	chr17	ENSMUSG00000051113	chr7
ENSMUSG00000026275	chr1	ENSMUSG00000015120	chr17	ENSMUSG00000060279	chr7
ENSMUSG00000101904	chr1	ENSMUSG00000077450	chr17	ENSMUSG00000042308	chr7
ENSMUSG00000067851	chr1	ENSMUSG00000017831	chr17	ENSMUSG00000039361	chr7
ENSMUSG00000096141	chr1	ENSMUSG00000024209	chr17	ENSMUSG00000031493	chr7
ENSMUSG00000042207	chr1	ENSMUSG00000045409	chr17	ENSMUSG00000019370	chr7
ENSMUSG00000026469	chr1	ENSMUSG00000024242	chr17	ENSMUSG00000055826	chr7
ENSMUSG00000026219	chr1	ENSMUSG00000062202	chr17	ENSMUSG00000060260	chr7
ENSMUSG00000042197	chr1	ENSMUSG00000071073	chr17	ENSMUSG00000070802	chr7
ENSMUSG00000060743	chr1	ENSMUSG00000059791	chr17	ENSMUSG00000015980	chr7
ENSMUSG00000037434	chr1	ENSMUSG00000036552	chr17	ENSMUSG00000063550	chr7
ENSMUSG00000026179	chr1	ENSMUSG00000049760	chr17	ENSMUSG00000030380	chr7
ENSMUSG00000026341	chr1	ENSMUSG00000046196	chr17	ENSMUSG00000030795	chr7
ENSMUSG00000025982	chr1	ENSMUSG00000037689	chr17	ENSMUSG00000042246	chr7
ENSMUSG00000025935	chr1	ENSMUSG00000036214	chr17	ENSMUSG00000073894	chr7
ENSMUSG00000026502	chr1	ENSMUSG00000056692	chr17	ENSMUSG00000002409	chr7
ENSMUSG00000026361	chr1	ENSMUSG00000097746	chr18	ENSMUSG00000040424	chr7
ENSMUSG00000026511	chr1	ENSMUSG00000044906	chr18	ENSMUSG00000060860	chr7
ENSMUSG00000026111	chr1	ENSMUSG00000103985	chr18	ENSMUSG00000030595	chr7
ENSMUSG00000040297	chr1	ENSMUSG00000087170	chr18	ENSMUSG00000041343	chr7
ENSMUSG00000026049	chr1	ENSMUSG00000024566	chr18	ENSMUSG00000035390	chr7
ENSMUSG00000040423	chr1	ENSMUSG00000073598	chr18	ENSMUSG00000015981	chr7
ENSMUSG00000046404	chr1	ENSMUSG00000024276	chr18	ENSMUSG00000062944	chr7
ENSMUSG00000026107	chr1	ENSMUSG00000103616	chr18	ENSMUSG00000020420	chr7
ENSMUSG00000033671	chr1	ENSMUSG00000097712	chr18	ENSMUSG00000038797	chr7
ENSMUSG00000051223	chr1	ENSMUSG00000046487	chr18	ENSMUSG00000047371	chr7
ENSMUSG00000070871	chr1	ENSMUSG00000024360	chr18	ENSMUSG00000043290	chr7
ENSMUSG00000026301	chr1	ENSMUSG00000025423	chr18	ENSMUSG00000074220	chr7
ENSMUSG00000013973	chr1	ENSMUSG00000024480	chr18	ENSMUSG00000055652	chr7
ENSMUSG00000026571	chr1	ENSMUSG00000079608	chr18	ENSMUSG00000070563	chr7
ENSMUSG00000042772	chr1	ENSMUSG00000024513	chr18	ENSMUSG00000072259	chr7
ENSMUSG00000037568	chr1	ENSMUSG00000041923	chr18	ENSMUSG00000053742	chr7
ENSMUSG00000056708	chr1	ENSMUSG00000024537	chr18	ENSMUSG00000060565	chr7
ENSMUSG00000046337	chr1	ENSMUSG00000024603	chr18	ENSMUSG00000048312	chr7
ENSMUSG00000073725	chr1	ENSMUSG00000037058	chr18	ENSMUSG00000043964	chr7

ENSMUSG00000052748	chr1	ENSMUSG00000032905	chr18	ENSMUSG00000062028	chr7
ENSMUSG00000046062	chr1	ENSMUSG00000024260	chr18	ENSMUSG00000030641	chr7
ENSMUSG00000050565	chr1	ENSMUSG00000024317	chr18	ENSMUSG00000044362	chr7
ENSMUSG00000026466	chr1	ENSMUSG00000024430	chr18	ENSMUSG00000078762	chr7
ENSMUSG00000026399	chr1	ENSMUSG00000024232	chr18	ENSMUSG00000030792	chr7
ENSMUSG00000006299	chr1	ENSMUSG00000037236	chr18	ENSMUSG00000013091	chr7
ENSMUSG00000079658	chr1	ENSMUSG00000036501	chr18	ENSMUSG00000030877	chr7
ENSMUSG00000052428	chr1	ENSMUSG00000024357	chr18	ENSMUSG00000066500	chr7
ENSMUSG00000058407	chr1	ENSMUSG00000073542	chr18	ENSMUSG00000030967	chr7
ENSMUSG00000040629	chr1	ENSMUSG00000057561	chr18	ENSMUSG00000008140	chr7
ENSMUSG00000026491	chr1	ENSMUSG00000033184	chr18	ENSMUSG00000043858	chr7
ENSMUSG00000041570	chr1	ENSMUSG00000024534	chr18	ENSMUSG00000093542	chr7
ENSMUSG00000027520	chr1	ENSMUSG00000024384	chr18	ENSMUSG00000031970	chr8
ENSMUSG00000047528	chr1	ENSMUSG00000063889	chr18	ENSMUSG00000049476	chr8
ENSMUSG00000026004	chr1	ENSMUSG00000024456	chr18	ENSMUSG00000103925	chr8
ENSMUSG00000026611	chr1	ENSMUSG00000051469	chr18	ENSMUSG00000063049	chr8
ENSMUSG00000026142	chr1	ENSMUSG00000037253	chr18	ENSMUSG00000037982	chr8
ENSMUSG00000038733	chr1	ENSMUSG00000102748	chr18	ENSMUSG00000097706	chr8
ENSMUSG00000073633	chr1	ENSMUSG00000091896	chr18	ENSMUSG00000063932	chr8
ENSMUSG00000026201	chr1	ENSMUSG00000024350	chr18	ENSMUSG00000094421	chr8
ENSMUSG00000026499	chr1	ENSMUSG00000034300	chr18	ENSMUSG00000080058	chr8
ENSMUSG00000033276	chr1	ENSMUSG00000024352	chr18	ENSMUSG00000033594	chr8
ENSMUSG00000026083	chr1	ENSMUSG00000100969	chr19	ENSMUSG00000031736	chr8
ENSMUSG00000044340	chr1	ENSMUSG00000085196	chr19	ENSMUSG00000048484	chr8
ENSMUSG00000056050	chr1	ENSMUSG00000099923	chr19	ENSMUSG00000097637	chr8
ENSMUSG00000026064	chr1	ENSMUSG00000092341	chr19	ENSMUSG00000071138	chr8
ENSMUSG00000039224	chr1	ENSMUSG00000092274	chr19	ENSMUSG00000099881	chr8
ENSMUSG00000025939	chr1	ENSMUSG00000098986	chr19	ENSMUSG00000074178	chr8
ENSMUSG00000081984	chr1	ENSMUSG00000035171	chr19	ENSMUSG00000054247	chr8
ENSMUSG00000026203	chr1	ENSMUSG00000079467	chr19	ENSMUSG00000069925	chr8
ENSMUSG00000026311	chr1	ENSMUSG00000043488	chr19	ENSMUSG00000097120	chr8
ENSMUSG00000025921	chr1	ENSMUSG00000082848	chr19	ENSMUSG00000085464	chr8
ENSMUSG00000026094	chr1	ENSMUSG00000092655	chr19	ENSMUSG00000031796	chr8
ENSMUSG00000023150	chr1	ENSMUSG00000033760	chr19	ENSMUSG00000098211	chr8
ENSMUSG00000026112	chr1	ENSMUSG00000058816	chr19	ENSMUSG00000048478	chr8
ENSMUSG00000026500	chr1	ENSMUSG00000089397	chr19	ENSMUSG00000043794	chr8
ENSMUSG00000100725	chr1	ENSMUSG00000087367	chr19	ENSMUSG00000097308	chr8
ENSMUSG00000097086	chr10	ENSMUSG00000102349	chr19	ENSMUSG00000045746	chr8
ENSMUSG00000086241	chr10	ENSMUSG00000098974	chr19	ENSMUSG00000023235	chr8
ENSMUSG00000093674	chr10	ENSMUSG00000098543	chr19	ENSMUSG00000074357	chr8
ENSMUSG00000105203	chr10	ENSMUSG00000038498	chr19	ENSMUSG00000103341	chr8
ENSMUSG00000020212	chr10	ENSMUSG00000010097	chr19	ENSMUSG00000031893	chr8
ENSMUSG00000078427	chr10	ENSMUSG00000037418	chr19	ENSMUSG00000097424	chr8
ENSMUSG00000069540	chr10	ENSMUSG00000024759	chr19	ENSMUSG00000041438	chr8
ENSMUSG00000097129	chr10	ENSMUSG00000024831	chr19	ENSMUSG00000019470	chr8
ENSMUSG00000097407	chr10	ENSMUSG00000025231	chr19	ENSMUSG00000031532	chr8
ENSMUSG00000045391	chr10	ENSMUSG00000024949	chr19	ENSMUSG00000056753	chr8
ENSMUSG00000038010	chr10	ENSMUSG00000024772	chr19	ENSMUSG00000097409	chr8
ENSMUSG00000087163	chr10	ENSMUSG00000025069	chr19	ENSMUSG00000054320	chr8
ENSMUSG00000099088	chr10	ENSMUSG00000071644	chr19	ENSMUSG00000054910	chr8

ENSMUSG00000087177	chr10	ENSMUSG00000024695	chr19	ENSMUSG00000011832	chr8
ENSMUSG00000025354	chr10	ENSMUSG00000056201	chr19	ENSMUSG00000037415	chr8
ENSMUSG00000090535	chr10	ENSMUSG00000033417	chr19	ENSMUSG00000041679	chr8
ENSMUSG00000061032	chr10	ENSMUSG00000025228	chr19	ENSMUSG00000031984	chr8
ENSMUSG00000020064	chr10	ENSMUSG00000023307	chr19	ENSMUSG00000031530	chr8
ENSMUSG00000097007	chr10	ENSMUSG00000047379	chr19	ENSMUSG00000036840	chr8
ENSMUSG00000035242	chr10	ENSMUSG00000047368	chr19	ENSMUSG00000000743	chr8
ENSMUSG00000092593	chr10	ENSMUSG00000024873	chr19	ENSMUSG00000003813	chr8
ENSMUSG00000003226	chr10	ENSMUSG00000071659	chr19	ENSMUSG00000090137	chr8
ENSMUSG00000091358	chr10	ENSMUSG00000039652	chr19	ENSMUSG00000031715	chr8
ENSMUSG00000044475	chr10	ENSMUSG00000040929	chr19	ENSMUSG00000071076	chr8
ENSMUSG00000040054	chr10	ENSMUSG00000038274	chr19	ENSMUSG00000004319	chr8
ENSMUSG00000097164	chr10	ENSMUSG00000025024	chr19	ENSMUSG00000031860	chr8
ENSMUSG00000087993	chr10	ENSMUSG00000025178	chr19	ENSMUSG00000033009	chr8
ENSMUSG00000069713	chr10	ENSMUSG00000025220	chr19	ENSMUSG00000031729	chr8
ENSMUSG00000020131	chr10	ENSMUSG00000024906	chr19	ENSMUSG00000036990	chr8
ENSMUSG00000097370	chr10	ENSMUSG00000007338	chr19	ENSMUSG00000031751	chr8
ENSMUSG00000050994	chr10	ENSMUSG00000024973	chr19	ENSMUSG00000031985	chr8
ENSMUSG00000019878	chr10	ENSMUSG00000024991	chr19	ENSMUSG00000057116	chr8
ENSMUSG00000019841	chr10	ENSMUSG00000039148	chr19	ENSMUSG00000031706	chr8
ENSMUSG00000020184	chr10	ENSMUSG00000024663	chr19	ENSMUSG00000039530	chr8
ENSMUSG00000004934	chr10	ENSMUSG00000075227	chr19	ENSMUSG00000031585	chr8
ENSMUSG00000020018	chr10	ENSMUSG00000035173	chr19	ENSMUSG00000035151	chr8
ENSMUSG00000015757	chr10	ENSMUSG00000024974	chr19	ENSMUSG00000036199	chr8
ENSMUSG00000019808	chr10	ENSMUSG00000024862	chr19	ENSMUSG00000031754	chr8
ENSMUSG00000034024	chr10	ENSMUSG00000024764	chr19	ENSMUSG00000036686	chr8
ENSMUSG00000019795	chr10	ENSMUSG00000024829	chr19	ENSMUSG00000048967	chr8
ENSMUSG00000060904	chr10	ENSMUSG00000074746	chr19	ENSMUSG00000015341	chr8
ENSMUSG00000038774	chr10	ENSMUSG00000035049	chr19	ENSMUSG00000031781	chr8
ENSMUSG00000003345	chr10	ENSMUSG00000074922	chr19	ENSMUSG00000050079	chr8
ENSMUSG00000033307	chr10	ENSMUSG00000024843	chr19	ENSMUSG00000039509	chr8
ENSMUSG00000020079	chr10	ENSMUSG00000056209	chr19	ENSMUSG00000031774	chr8
ENSMUSG00000034674	chr10	ENSMUSG00000090291	chr19	ENSMUSG00000014856	chr8
ENSMUSG00000078429	chr10	ENSMUSG00000033863	chr19	ENSMUSG00000031600	chr8
ENSMUSG00000025402	chr10	ENSMUSG00000025047	chr19	ENSMUSG00000033624	chr8
ENSMUSG00000025353	chr10	ENSMUSG00000006456	chr19	ENSMUSG00000042870	chr8
ENSMUSG00000039810	chr10	ENSMUSG00000096370	chr19	ENSMUSG00000014470	chr8
ENSMUSG00000025374	chr10	ENSMUSG00000024969	chr19	ENSMUSG00000037363	chr8
ENSMUSG00000038122	chr10	ENSMUSG00000035179	chr19	ENSMUSG00000051648	chr8
ENSMUSG00000020072	chr10	ENSMUSG00000071528	chr19	ENSMUSG00000008129	chr8
ENSMUSG00000020069	chr10	ENSMUSG00000026931	chr19	ENSMUSG00000031809	chr8
ENSMUSG00000020074	chr10	ENSMUSG00000047604	chr19	ENSMUSG00000022322	chr8
ENSMUSG00000009291	chr10	ENSMUSG00000067199	chr19	ENSMUSG00000045248	chr8
ENSMUSG00000037608	chr10	ENSMUSG00000024970	chr19	ENSMUSG00000031776	chr8
ENSMUSG00000025373	chr10	ENSMUSG00000049670	chr19	ENSMUSG00000038069	chr8
ENSMUSG00000040462	chr10	ENSMUSG00000074507	chr2	ENSMUSG00000031727	chr8
ENSMUSG00000047648	chr10	ENSMUSG00000086515	chr2	ENSMUSG00000000759	chr8
ENSMUSG00000025409	chr10	ENSMUSG00000075014	chr2	ENSMUSG00000049482	chr8
ENSMUSG00000060843	chr10	ENSMUSG00000081496	chr2	ENSMUSG00000069633	chr8
ENSMUSG00000036955	chr10	ENSMUSG00000027327	chr2	ENSMUSG00000031782	chr8

ENSMUSG00000061589	chr10	ENSMUSG00000103542	chr2	ENSMUSG00000033545	chr8
ENSMUSG00000019814	chr10	ENSMUSG00000085436	chr2	ENSMUSG00000059854	chr8
ENSMUSG00000061315	chr10	ENSMUSG00000005510	chr2	ENSMUSG00000031545	chr8
ENSMUSG00000009115	chr10	ENSMUSG00000085818	chr2	ENSMUSG00000031913	chr8
ENSMUSG00000035890	chr10	ENSMUSG00000087205	chr2	ENSMUSG00000031967	chr8
ENSMUSG00000020198	chr10	ENSMUSG00000058812	chr2	ENSMUSG00000055148	chr8
ENSMUSG00000071072	chr10	ENSMUSG00000099632	chr2	ENSMUSG00000070002	chr8
ENSMUSG00000036478	chr10	ENSMUSG00000086348	chr2	ENSMUSG00000052906	chr8
ENSMUSG00000019977	chr10	ENSMUSG00000080374	chr2	ENSMUSG00000038250	chr8
ENSMUSG00000020024	chr10	ENSMUSG00000026842	chr2	ENSMUSG00000040028	chr8
ENSMUSG00000048696	chr10	ENSMUSG00000104603	chr2	ENSMUSG00000033658	chr8
ENSMUSG00000020124	chr10	ENSMUSG00000026806	chr2	ENSMUSG00000018796	chr8
ENSMUSG00000020075	chr10	ENSMUSG00000093242	chr2	ENSMUSG00000074449	chr8
ENSMUSG00000020076	chr10	ENSMUSG00000098024	chr2	ENSMUSG00000096265	chr8
ENSMUSG00000009293	chr10	ENSMUSG00000002718	chr2	ENSMUSG00000095294	chr8
ENSMUSG00000074781	chr10	ENSMUSG00000075036	chr2	ENSMUSG00000031529	chr8
ENSMUSG00000020307	chr10	ENSMUSG00000087549	chr2	ENSMUSG00000047654	chr8
ENSMUSG00000020109	chr10	ENSMUSG00000059540	chr2	ENSMUSG00000002948	chr8
ENSMUSG00000003068	chr10	ENSMUSG00000087405	chr2	ENSMUSG00000031728	chr8
ENSMUSG00000040021	chr10	ENSMUSG00000017767	chr2	ENSMUSG00000036934	chr8
ENSMUSG00000020234	chr10	ENSMUSG00000027620	chr2	ENSMUSG00000074247	chr8
ENSMUSG00000020107	chr10	ENSMUSG00000086714	chr2	ENSMUSG00000046691	chr8
ENSMUSG00000040006	chr10	ENSMUSG00000075015	chr2	ENSMUSG00000087687	chr8
ENSMUSG00000020096	chr10	ENSMUSG00000035877	chr2	ENSMUSG00000031847	chr8
ENSMUSG00000019809	chr10	ENSMUSG00000087633	chr2	ENSMUSG00000031452	chr8
ENSMUSG00000020224	chr10	ENSMUSG00000043909	chr2	ENSMUSG00000003824	chr8
ENSMUSG00000047025	chr10	ENSMUSG00000087519	chr2	ENSMUSG00000093619	chr9
ENSMUSG00000035504	chr10	ENSMUSG00000027641	chr2	ENSMUSG00000034252	chr9
ENSMUSG00000085143	chr11	ENSMUSG00000038180	chr2	ENSMUSG00000065579	chr9
ENSMUSG00000080152	chr11	ENSMUSG00000044916	chr2	ENSMUSG00000074256	chr9
ENSMUSG00000053263	chr11	ENSMUSG00000055897	chr2	ENSMUSG00000042688	chr9
ENSMUSG00000087184	chr11	ENSMUSG00000005882	chr2	ENSMUSG00000084786	chr9
ENSMUSG00000080833	chr11	ENSMUSG00000046229	chr2	ENSMUSG00000098875	chr9
ENSMUSG00000069890	chr11	ENSMUSG00000039463	chr2	ENSMUSG00000102244	chr9
ENSMUSG00000042549	chr11	ENSMUSG00000027071	chr2	ENSMUSG00000039048	chr9
ENSMUSG00000085609	chr11	ENSMUSG00000002733	chr2	ENSMUSG00000090207	chr9
ENSMUSG00000086808	chr11	ENSMUSG00000101645	chr2	ENSMUSG00000102300	chr9
ENSMUSG00000099630	chr11	ENSMUSG00000017707	chr2	ENSMUSG00000103103	chr9
ENSMUSG00000086493	chr11	ENSMUSG00000027282	chr2	ENSMUSG00000038717	chr9
ENSMUSG00000087574	chr11	ENSMUSG00000034154	chr2	ENSMUSG00000105072	chr9
ENSMUSG00000036309	chr11	ENSMUSG00000026739	chr2	ENSMUSG00000086918	chr9
ENSMUSG00000009079	chr11	ENSMUSG00000002458	chr2	ENSMUSG00000065587	chr9
ENSMUSG00000081296	chr11	ENSMUSG00000093752	chr2	ENSMUSG00000093803	chr9
ENSMUSG00000086193	chr11	ENSMUSG00000078919	chr2	ENSMUSG00000032316	chr9
ENSMUSG00000056962	chr11	ENSMUSG00000002546	chr2	ENSMUSG00000105497	chr9
ENSMUSG00000075420	chr11	ENSMUSG00000025816	chr2	ENSMUSG00000092915	chr9
ENSMUSG00000065537	chr11	ENSMUSG00000026915	chr2	ENSMUSG00000059237	chr9
ENSMUSG00000059278	chr11	ENSMUSG00000074576	chr2	ENSMUSG00000032349	chr9
ENSMUSG00000018379	chr11	ENSMUSG00000027602	chr2	ENSMUSG00000042487	chr9
ENSMUSG00000018238	chr11	ENSMUSG00000039050	chr2	ENSMUSG00000074146	chr9

ENSMUSG00000064437	chr11	ENSMUSG00000037885	chr2	ENSMUSG00000032300	chr9
ENSMUSG00000014195	chr11	ENSMUSG00000007659	chr2	ENSMUSG00000065492	chr9
ENSMUSG00000075576	chr11	ENSMUSG00000026643	chr2	ENSMUSG00000103785	chr9
ENSMUSG00000038217	chr11	ENSMUSG00000005802	chr2	ENSMUSG00000086914	chr9
ENSMUSG00000086841	chr11	ENSMUSG00000036752	chr2	ENSMUSG00000031927	chr9
ENSMUSG00000086716	chr11	ENSMUSG00000027603	chr2	ENSMUSG00000097021	chr9
ENSMUSG00000047284	chr11	ENSMUSG00000074881	chr2	ENSMUSG00000079564	chr9
ENSMUSG00000001323	chr11	ENSMUSG00000026926	chr2	ENSMUSG00000097099	chr9
ENSMUSG00000087039	chr11	ENSMUSG00000061136	chr2	ENSMUSG00000057367	chr9
ENSMUSG00000087120	chr11	ENSMUSG00000090100	chr2	ENSMUSG00000007656	chr9
ENSMUSG00000099681	chr11	ENSMUSG00000078129	chr2	ENSMUSG00000032171	chr9
ENSMUSG00000043644	chr11	ENSMUSG00000042854	chr2	ENSMUSG00000032280	chr9
ENSMUSG00000040667	chr11	ENSMUSG00000033411	chr2	ENSMUSG00000062270	chr9
ENSMUSG00000085890	chr11	ENSMUSG00000074673	chr2	ENSMUSG00000032178	chr9
ENSMUSG00000081749	chr11	ENSMUSG00000026885	chr2	ENSMUSG00000032328	chr9
ENSMUSG00000034587	chr11	ENSMUSG00000027433	chr2	ENSMUSG00000033543	chr9
ENSMUSG00000083175	chr11	ENSMUSG00000043415	chr2	ENSMUSG00000032540	chr9
ENSMUSG00000081297	chr11	ENSMUSG00000026857	chr2	ENSMUSG00000032185	chr9
ENSMUSG00000065590	chr11	ENSMUSG00000027079	chr2	ENSMUSG00000025794	chr9
ENSMUSG00000044795	chr11	ENSMUSG00000040812	chr2	ENSMUSG00000050912	chr9
ENSMUSG00000020859	chr11	ENSMUSG00000027502	chr2	ENSMUSG00000032479	chr9
ENSMUSG00000086826	chr11	ENSMUSG00000043241	chr2	ENSMUSG00000032412	chr9
ENSMUSG00000018541	chr11	ENSMUSG00000027573	chr2	ENSMUSG00000032293	chr9
ENSMUSG00000053574	chr11	ENSMUSG00000027304	chr2	ENSMUSG00000025234	chr9
ENSMUSG00000043648	chr11	ENSMUSG00000026924	chr2	ENSMUSG00000064145	chr9
ENSMUSG00000065734	chr11	ENSMUSG00000027067	chr2	ENSMUSG00000032294	chr9
ENSMUSG00000085991	chr11	ENSMUSG00000027288	chr2	ENSMUSG00000003131	chr9
ENSMUSG00000085148	chr11	ENSMUSG00000047841	chr2	ENSMUSG00000025894	chr9
ENSMUSG00000086725	chr11	ENSMUSG00000038914	chr2	ENSMUSG00000032599	chr9
ENSMUSG00000096983	chr11	ENSMUSG00000026812	chr2	ENSMUSG00000032594	chr9
ENSMUSG00000105025	chr11	ENSMUSG00000038932	chr2	ENSMUSG00000032575	chr9
ENSMUSG00000085088	chr11	ENSMUSG00000035666	chr2	ENSMUSG00000040325	chr9
ENSMUSG00000098948	chr11	ENSMUSG00000051149	chr2	ENSMUSG00000010044	chr9
ENSMUSG00000084079	chr11	ENSMUSG00000050043	chr2	ENSMUSG00000042396	chr9
ENSMUSG00000045942	chr11	ENSMUSG00000034723	chr2	ENSMUSG00000032437	chr9
ENSMUSG00000017802	chr11	ENSMUSG00000026864	chr2	ENSMUSG00000032580	chr9
ENSMUSG00000098943	chr11	ENSMUSG00000075271	chr2	ENSMUSG00000032567	chr9
ENSMUSG00000050860	chr11	ENSMUSG00000075273	chr2	ENSMUSG00000032212	chr9
ENSMUSG00000056579	chr11	ENSMUSG00000075272	chr2	ENSMUSG00000019039	chr9
ENSMUSG00000002064	chr11	ENSMUSG00000026887	chr2	ENSMUSG00000032803	chr9
ENSMUSG00000002052	chr11	ENSMUSG00000026889	chr2	ENSMUSG00000042293	chr9
ENSMUSG00000047205	chr11	ENSMUSG00000063145	chr2	ENSMUSG00000039977	chr9
ENSMUSG00000040489	chr11	ENSMUSG00000070708	chr2	ENSMUSG00000031922	chr9
ENSMUSG00000047126	chr11	ENSMUSG00000075302	chr2	ENSMUSG00000032115	chr9
ENSMUSG00000038485	chr11	ENSMUSG00000027236	chr2	ENSMUSG00000064225	chr9
ENSMUSG00000035530	chr11	ENSMUSG00000026940	chr2	ENSMUSG00000013822	chr9
ENSMUSG00000049470	chr11	ENSMUSG00000075249	chr2	ENSMUSG00000070283	chr9
ENSMUSG00000038909	chr11	ENSMUSG00000027344	chr2	ENSMUSG00000046111	chr9
ENSMUSG00000019505	chr11	ENSMUSG00000009207	chr2	ENSMUSG00000045620	chr9
ENSMUSG00000018401	chr11	ENSMUSG00000027080	chr2	ENSMUSG00000070284	chr9

ENSMUSG00000018567	chr11	ENSMUSG00000027517	chr2	ENSMUSG00000010067	chr9
ENSMUSG00000020368	chr11	ENSMUSG00000026646	chr2	ENSMUSG00000038664	chr9
ENSMUSG00000035152	chr11	ENSMUSG00000027184	chr2	ENSMUSG00000037808	chr9
ENSMUSG00000046756	chr11	ENSMUSG00000038085	chr2	ENSMUSG00000044791	chr9
ENSMUSG00000020698	chr11	ENSMUSG00000039164	chr2	ENSMUSG00000000167	chr9
ENSMUSG00000038994	chr11	ENSMUSG00000026790	chr2	ENSMUSG00000032051	chr9
ENSMUSG00000035198	chr11	ENSMUSG00000039804	chr2	ENSMUSG00000049932	chr9
ENSMUSG00000048076	chr11	ENSMUSG00000026817	chr2	ENSMUSG00000032010	chr9
ENSMUSG00000020882	chr11	ENSMUSG00000026807	chr2	ENSMUSG00000023473	chr9
ENSMUSG00000034227	chr11	ENSMUSG00000059005	chr2	ENSMUSG00000032285	chr9
ENSMUSG00000020483	chr11	ENSMUSG00000027519	chr2	ENSMUSG00000032489	chr9
ENSMUSG00000025162	chr11	ENSMUSG00000026819	chr2	ENSMUSG00000036768	chr9
ENSMUSG00000062825	chr11	ENSMUSG00000027605	chr2	ENSMUSG00000037112	chr9
ENSMUSG00000036620	chr11	ENSMUSG00000027361	chr2	ENSMUSG00000035032	chr9
ENSMUSG00000020134	chr11	ENSMUSG00000039834	chr2	ENSMUSG00000047412	chr9
ENSMUSG00000020723	chr11	ENSMUSG00000005505	chr2	ENSMUSG00000054792	chr9
ENSMUSG00000044894	chr11	ENSMUSG00000051154	chr2	ENSMUSG00000025893	chr9
ENSMUSG00000020867	chr11	ENSMUSG00000026927	chr2	ENSMUSG00000032297	chr9
ENSMUSG00000059474	chr11	ENSMUSG00000050592	chr2	ENSMUSG00000053070	chr9
ENSMUSG00000039110	chr11	ENSMUSG00000038375	chr2	ENSMUSG00000040729	chr9
ENSMUSG00000020448	chr11	ENSMUSG00000026809	chr2	ENSMUSG00000032551	chr9
ENSMUSG00000020328	chr11	ENSMUSG00000045794	chr2	ENSMUSG00000025652	chr9
ENSMUSG00000018570	chr11	ENSMUSG00000044627	chr2	ENSMUSG00000059820	chr9
ENSMUSG00000020803	chr11	ENSMUSG00000026960	chr2	ENSMUSG00000097298	chr9
ENSMUSG00000005204	chr11	ENSMUSG00000027284	chr2	ENSMUSG00000095293	chrX
ENSMUSG00000018733	chr11	ENSMUSG00000026963	chr2	ENSMUSG00000094714	chrX
ENSMUSG00000014245	chr11	ENSMUSG00000074884	chr2	ENSMUSG00000067909	chrX
ENSMUSG00000014177	chr11	ENSMUSG00000105350	chr3	ENSMUSG00000054727	chrX
ENSMUSG00000020801	chr11	ENSMUSG00000097724	chr3	ENSMUSG00000080725	chrX
ENSMUSG00000046010	chr11	ENSMUSG00000086923	chr3	ENSMUSG00000073247	chrX
ENSMUSG00000020514	chr11	ENSMUSG00000085956	chr3	ENSMUSG00000071788	chrX
ENSMUSG00000038290	chr11	ENSMUSG00000090408	chr3	ENSMUSG00000082639	chrX
ENSMUSG00000020530	chr11	ENSMUSG00000105535	chr3	ENSMUSG00000096468	chrX
ENSMUSG00000044949	chr11	ENSMUSG00000105852	chr3	ENSMUSG00000095546	chrX
ENSMUSG00000017146	chr11	ENSMUSG00000076255	chr3	ENSMUSG00000096620	chrX
ENSMUSG00000020525	chr11	ENSMUSG00000076456	chr3	ENSMUSG00000073245	chrX
ENSMUSG00000020740	chr11	ENSMUSG00000097032	chr3	ENSMUSG00000073257	chrX
ENSMUSG00000007850	chr11	ENSMUSG00000038712	chr3	ENSMUSG00000073255	chrX
ENSMUSG00000018501	chr11	ENSMUSG00000057359	chr3	ENSMUSG00000096457	chrX
ENSMUSG00000020733	chr11	ENSMUSG00000105201	chr3	ENSMUSG00000073267	chrX
ENSMUSG00000078627	chr11	ENSMUSG00000038619	chr3	ENSMUSG00000093923	chrX
ENSMUSG00000018415	chr11	ENSMUSG00000036745	chr3	ENSMUSG00000094759	chrX
ENSMUSG00000034757	chr11	ENSMUSG00000105057	chr3	ENSMUSG00000094624	chrX
ENSMUSG00000090266	chr11	ENSMUSG00000039831	chr3	ENSMUSG00000095887	chrX
ENSMUSG00000015837	chr11	ENSMUSG00000028188	chr3	ENSMUSG00000096867	chrX
ENSMUSG00000049396	chr11	ENSMUSG00000093553	chr3	ENSMUSG00000094577	chrX
ENSMUSG00000020807	chr11	ENSMUSG00000103848	chr3	ENSMUSG00000096915	chrX
ENSMUSG00000041623	chr11	ENSMUSG00000053957	chr3	ENSMUSG00000094596	chrX
ENSMUSG00000037573	chr11	ENSMUSG00000028140	chr3	ENSMUSG00000095934	chrX
ENSMUSG00000020849	chr11	ENSMUSG00000027968	chr3	ENSMUSG00000094309	chrX

ENSMUSG00000020330	chr11	ENSMUSG00000103041	chr3	ENSMUSG00000094876	chrX
ENSMUSG00000005198	chr11	ENSMUSG00000044468	chr3	ENSMUSG00000094391	chrX
ENSMUSG00000038178	chr11	ENSMUSG00000050936	chr3	ENSMUSG00000095229	chrX
ENSMUSG00000017119	chr11	ENSMUSG00000028059	chr3	ENSMUSG00000095558	chrX
ENSMUSG00000042709	chr11	ENSMUSG00000070392	chr3	ENSMUSG00000094307	chrX
ENSMUSG00000020894	chr11	ENSMUSG00000084863	chr3	ENSMUSG00000095814	chrX
ENSMUSG00000032889	chr11	ENSMUSG00000028121	chr3	ENSMUSG00000101294	chrX
ENSMUSG00000020831	chr11	ENSMUSG00000093577	chr3	ENSMUSG00000101500	chrX
ENSMUSG00000049811	chr11	ENSMUSG00000019338	chr3	ENSMUSG00000094273	chrX
ENSMUSG00000020462	chr11	ENSMUSG00000028137	chr3	ENSMUSG00000094558	chrX
ENSMUSG00000076433	chr11	ENSMUSG00000028035	chr3	ENSMUSG00000096426	chrX
ENSMUSG00000035992	chr11	ENSMUSG00000004896	chr3	ENSMUSG00000095637	chrX
ENSMUSG00000018479	chr11	ENSMUSG00000105610	chr3	ENSMUSG00000094352	chrX
ENSMUSG00000018412	chr11	ENSMUSG00000027804	chr3	ENSMUSG00000094155	chrX
ENSMUSG00000018372	chr11	ENSMUSG00000097204	chr3	ENSMUSG00000101381	chrX
ENSMUSG00000049287	chr11	ENSMUSG00000106587	chr3	ENSMUSG00000095716	chrX
ENSMUSG00000034175	chr11	ENSMUSG00000097786	chr3	ENSMUSG00000100249	chrX
ENSMUSG00000037275	chr11	ENSMUSG00000027829	chr3	ENSMUSG00000094361	chrX
ENSMUSG00000018776	chr11	ENSMUSG00000049940	chr3	ENSMUSG00000096194	chrX
ENSMUSG00000007646	chr11	ENSMUSG00000028034	chr3	ENSMUSG00000094860	chrX
ENSMUSG00000010342	chr11	ENSMUSG00000036432	chr3	ENSMUSG00000078324	chrX
ENSMUSG00000034120	chr11	ENSMUSG00000027668	chr3	ENSMUSG00000099711	chrX
ENSMUSG00000020456	chr11	ENSMUSG00000027680	chr3	ENSMUSG00000078218	chrX
ENSMUSG00000020818	chr11	ENSMUSG00000027719	chr3	ENSMUSG00000078213	chrX
ENSMUSG00000038057	chr11	ENSMUSG00000001416	chr3	ENSMUSG00000094727	chrX
ENSMUSG00000020463	chr11	ENSMUSG00000081058	chr3	ENSMUSG00000096097	chrX
ENSMUSG00000034543	chr11	ENSMUSG00000093769	chr3	ENSMUSG00000101819	chrX
ENSMUSG00000078249	chr11	ENSMUSG00000074403	chr3	ENSMUSG00000099443	chrX
ENSMUSG00000061666	chr11	ENSMUSG00000059743	chr3	ENSMUSG00000100626	chrX
ENSMUSG00000000804	chr11	ENSMUSG00000028271	chr3	ENSMUSG00000095445	chrX
ENSMUSG00000020358	chr11	ENSMUSG00000068747	chr3	ENSMUSG00000095655	chrX
ENSMUSG00000069769	chr11	ENSMUSG00000064068	chr3	ENSMUSG00000104702	chrX
ENSMUSG00000020719	chr11	ENSMUSG00000047213	chr3	ENSMUSG00000087133	chrX
ENSMUSG00000059796	chr11	ENSMUSG00000027706	chr3	ENSMUSG00000083089	chrX
ENSMUSG00000003119	chr11	ENSMUSG00000000563	chr3	ENSMUSG00000082297	chrX
ENSMUSG00000020389	chr11	ENSMUSG00000027822	chr3	ENSMUSG00000080498	chrX
ENSMUSG00000020390	chr11	ENSMUSG00000048039	chr3	ENSMUSG00000045217	chrX
ENSMUSG00000020794	chr11	ENSMUSG00000048416	chr3	ENSMUSG00000078214	chrX
ENSMUSG00000017837	chr11	ENSMUSG00000044098	chr3	ENSMUSG00000104042	chrX
ENSMUSG00000018740	chr11	ENSMUSG00000038495	chr3	ENSMUSG00000094698	chrX
ENSMUSG00000040365	chr11	ENSMUSG00000042390	chr3	ENSMUSG00000025287	chrX
ENSMUSG00000046755	chr11	ENSMUSG00000046722	chr3	ENSMUSG00000080792	chrX
ENSMUSG00000020481	chr11	ENSMUSG00000075700	chr3	ENSMUSG00000031105	chrX
ENSMUSG00000018932	chr11	ENSMUSG00000068523	chr3	ENSMUSG00000096716	chrX
ENSMUSG00000048732	chr11	ENSMUSG00000002228	chr3	ENSMUSG00000082403	chrX
ENSMUSG00000018449	chr11	ENSMUSG00000053192	chr3	ENSMUSG00000071721	chrX
ENSMUSG00000050628	chr11	ENSMUSG00000105204	chr3	ENSMUSG00000094999	chrX
ENSMUSG00000040850	chr11	ENSMUSG00000068860	chr3	ENSMUSG00000105122	chrX
ENSMUSG00000049154	chr11	ENSMUSG00000042520	chr3	ENSMUSG00000057805	chrX
ENSMUSG00000020482	chr11	ENSMUSG00000025757	chr3	ENSMUSG00000056815	chrX

ENSMUSG00000018543	chr11	ENSMUSG00000001017	chr3	ENSMUSG00000096374	chrX
ENSMUSG00000042200	chr11	ENSMUSG00000036580	chr3	ENSMUSG00000080933	chrX
ENSMUSG00000034031	chr11	ENSMUSG00000027942	chr3	ENSMUSG00000085157	chrX
ENSMUSG00000044787	chr11	ENSMUSG00000015711	chr3	ENSMUSG00000081606	chrX
ENSMUSG00000020434	chr11	ENSMUSG00000036825	chr3	ENSMUSG00000095890	chrX
ENSMUSG00000045377	chr11	ENSMUSG00000039068	chr3	ENSMUSG00000096376	chrX
ENSMUSG00000010841	chr11	ENSMUSG00000040412	chr3	ENSMUSG00000079871	chrX
ENSMUSG00000041629	chr11	ENSMUSG00000000561	chr3	ENSMUSG00000105455	chrX
ENSMUSG00000054079	chr11	ENSMUSG00000063052	chr3	ENSMUSG00000084324	chrX
ENSMUSG00000051890	chr12	ENSMUSG00000048109	chr3	ENSMUSG00000094317	chrX
ENSMUSG00000052673	chr12	ENSMUSG00000041842	chr3	ENSMUSG00000035522	chrX
ENSMUSG00000097055	chr12	ENSMUSG00000027782	chr3	ENSMUSG00000104666	chrX
ENSMUSG00000086315	chr12	ENSMUSG00000033502	chr3	ENSMUSG00000094118	chrX
ENSMUSG00000071265	chr12	ENSMUSG00000105827	chr3	ENSMUSG00000096235	chrX
ENSMUSG00000099759	chr12	ENSMUSG00000063954	chr3	ENSMUSG00000105318	chrX
ENSMUSG00000085664	chr12	ENSMUSG00000064220	chr3	ENSMUSG00000081224	chrX
ENSMUSG00000021101	chr12	ENSMUSG00000089911	chr3	ENSMUSG00000095850	chrX
ENSMUSG00000059695	chr12	ENSMUSG00000068856	chr3	ENSMUSG00000085880	chrX
ENSMUSG00000085820	chr12	ENSMUSG00000027905	chr3	ENSMUSG00000025527	chrX
ENSMUSG00000100315	chr12	ENSMUSG00000008730	chr3	ENSMUSG00000025271	chrX
ENSMUSG00000093579	chr12	ENSMUSG00000033147	chr3	ENSMUSG00000009941	chrX
ENSMUSG00000020669	chr12	ENSMUSG00000078578	chr3	ENSMUSG00000025037	chrX
ENSMUSG00000089371	chr12	ENSMUSG00000034317	chr3	ENSMUSG00000025862	chrX
ENSMUSG00000093618	chr12	ENSMUSG00000055865	chr3	ENSMUSG00000031378	chrX
ENSMUSG00000021096	chr12	ENSMUSG00000055436	chr3	ENSMUSG00000035491	chrX
ENSMUSG00000076432	chr12	ENSMUSG00000051278	chr3	ENSMUSG00000031118	chrX
ENSMUSG00000091071	chr12	ENSMUSG00000010538	chr3	ENSMUSG00000025151	chrX
ENSMUSG00000092233	chr12	ENSMUSG00000074579	chr3	ENSMUSG00000093806	chrX
ENSMUSG00000097494	chr12	ENSMUSG00000037266	chr4	ENSMUSG00000100194	chrX
ENSMUSG00000035983	chr12	ENSMUSG00000096930	chr4	ENSMUSG00000100133	chrX
ENSMUSG00000064663	chr12	ENSMUSG00000085525	chr4	ENSMUSG00000031095	chrX
ENSMUSG00000020590	chr12	ENSMUSG00000095054	chr4	ENSMUSG00000046615	chrX
ENSMUSG00000021248	chr12	ENSMUSG00000087044	chr4	ENSMUSG00000035232	chrX
ENSMUSG00000079179	chr12	ENSMUSG00000044125	chr4	ENSMUSG00000072100	chrX
ENSMUSG00000020948	chr12	ENSMUSG00000087513	chr4	ENSMUSG00000049775	chrX
ENSMUSG00000100789	chr12	ENSMUSG00000085085	chr4	ENSMUSG00000025038	chrX
ENSMUSG00000021290	chr12	ENSMUSG00000028612	chr4	ENSMUSG00000051582	chrX
ENSMUSG00000006360	chr12	ENSMUSG00000082925	chr4	ENSMUSG00000002015	chrX
ENSMUSG00000021087	chr12	ENSMUSG00000059482	chr4	ENSMUSG00000041718	chrX
ENSMUSG00000008822	chr12	ENSMUSG00000012126	chr4	ENSMUSG00000073139	chrX
ENSMUSG00000062198	chr12	ENSMUSG00000054351	chr4	ENSMUSG00000056537	chrX
ENSMUSG00000020570	chr12	ENSMUSG00000086046	chr4	ENSMUSG00000001964	chrX
ENSMUSG00000021067	chr12	ENSMUSG00000088351	chr4	ENSMUSG00000060090	chrX
ENSMUSG00000021134	chr12	ENSMUSG00000078492	chr4	ENSMUSG00000034403	chrX
ENSMUSG00000021111	chr12	ENSMUSG00000082195	chr4	ENSMUSG00000067194	chrX
ENSMUSG00000041781	chr12	ENSMUSG00000085037	chr4	ENSMUSG00000079606	chrX
ENSMUSG00000011179	chr12	ENSMUSG00000097661	chr4	ENSMUSG00000031330	chrX
ENSMUSG00000079108	chr12	ENSMUSG00000073888	chr4	ENSMUSG00000048970	chrX
ENSMUSG00000021024	chr12	ENSMUSG00000052912	chr4	ENSMUSG00000052676	chrX
ENSMUSG00000047446	chr12	ENSMUSG00000083027	chr4	ENSMUSG00000071686	chrX

ENSMUSG00000044573	chr12	ENSMUSG00000106355	chr4	ENSMUSG00000105993	chrX
ENSMUSG00000020955	chr12	ENSMUSG00000085614	chr4	ENSMUSG00000078320	chrX
ENSMUSG00000021236	chr12	ENSMUSG00000048406	chr4	ENSMUSG00000031256	chrX
ENSMUSG00000062929	chr12	ENSMUSG00000046667	chr4	ENSMUSG00000031386	chrX
ENSMUSG00000021097	chr12	ENSMUSG00000086654	chr4	ENSMUSG00000047079	chrX
ENSMUSG00000021076	chr12	ENSMUSG00000073731	chr4	ENSMUSG00000035725	chrX
ENSMUSG00000020636	chr12	ENSMUSG00000096233	chr4	ENSMUSG00000031181	chrX
ENSMUSG00000021257	chr12	ENSMUSG00000064387	chr4	ENSMUSG00000079536	chrX
ENSMUSG00000049792	chr12	ENSMUSG00000097337	chr4	ENSMUSG00000079532	chrX
ENSMUSG00000035293	chr12	ENSMUSG00000081179	chr4	ENSMUSG00000079534	chrX
ENSMUSG00000020585	chr12	ENSMUSG00000065353	chr4	ENSMUSG00000079531	chrX
ENSMUSG00000044715	chr12	ENSMUSG00000085241	chr4	ENSMUSG00000100708	chrY
ENSMUSG00000021127	chr12	ENSMUSG00000081169	chr4	ENSMUSG00000095135	chrY
ENSMUSG00000020650	chr12	ENSMUSG00000073716	chr4	ENSMUSG00000101471	chrY
ENSMUSG00000021203	chr12	ENSMUSG00000100396	chr4	ENSMUSG00000101766	chrY
ENSMUSG00000041341	chr12	ENSMUSG00000093617	chr4	ENSMUSG00000101915	chrY
ENSMUSG00000021244	chr12	ENSMUSG00000029068	chr4	ENSMUSG00000096016	chrY
ENSMUSG00000020659	chr12	ENSMUSG00000048003	chr4	ENSMUSG00000101933	chrY
ENSMUSG00000041712	chr12	ENSMUSG00000028745	chr4	ENSMUSG00000101286	chrY
ENSMUSG00000021235	chr12	ENSMUSG00000005045	chr4	ENSMUSG00000102122	chrY
ENSMUSG00000021189	chr12	ENSMUSG00000045004	chr4	ENSMUSG00000099856	chrY
ENSMUSG00000064326	chr12	ENSMUSG00000028559	chr4	ENSMUSG00000099531	chrY
ENSMUSG00000045064	chr12	ENSMUSG00000028964	chr4	ENSMUSG00000102668	chrY
ENSMUSG00000057265	chr12	ENSMUSG00000028651	chr4	ENSMUSG00000100055	chrY
ENSMUSG00000021007	chr12	ENSMUSG00000028790	chr4	ENSMUSG00000099541	chrY
ENSMUSG00000021003	chr12	ENSMUSG00000028789	chr4	ENSMUSG00000104191	chrY
ENSMUSG00000002996	chr12	ENSMUSG00000028793	chr4	ENSMUSG00000100726	chrY
ENSMUSG00000049106	chr12	ENSMUSG00000042446	chr4	ENSMUSG00000101158	chrY
ENSMUSG00000020954	chr12	ENSMUSG00000028234	chr4	ENSMUSG00000100667	chrY
ENSMUSG00000037594	chr12	ENSMUSG00000058183	chr4	ENSMUSG00000095606	chrY
ENSMUSG00000047832	chr12	ENSMUSG00000053510	chr4	ENSMUSG00000101396	chrY
ENSMUSG00000010608	chr12	ENSMUSG00000051276	chr4	ENSMUSG00000100902	chrY
ENSMUSG00000021188	chr12	ENSMUSG00000070980	chr4	ENSMUSG00000096178	chrY
ENSMUSG00000054003	chr12	ENSMUSG00000029001	chr4	ENSMUSG00000100892	chrY
ENSMUSG00000021281	chr12	ENSMUSG00000041556	chr4	ENSMUSG00000099925	chrY
ENSMUSG00000021234	chr12	ENSMUSG00000028669	chr4	ENSMUSG00000094570	chrY
ENSMUSG00000035614	chr12	ENSMUSG00000048772	chr4	ENSMUSG00000096902	chrY
ENSMUSG00000020562	chr12	ENSMUSG00000038024	chr4	ENSMUSG00000101399	chrY
ENSMUSG00000066637	chr12	ENSMUSG00000028851	chr4	ENSMUSG00000103919	chrY
ENSMUSG00000020664	chr12	ENSMUSG00000040550	chr4	ENSMUSG00000100939	chrY
ENSMUSG00000021282	chr12	ENSMUSG00000028411	chr4	ENSMUSG00000099894	chrY
ENSMUSG00000054383	chr12	ENSMUSG00000040659	chr4	ENSMUSG00000100485	chrY
ENSMUSG00000014905	chr12	ENSMUSG00000028647	chr4	ENSMUSG00000100535	chrY
ENSMUSG00000059114	chr12	ENSMUSG00000028291	chr4	ENSMUSG00000096650	chrY
ENSMUSG00000037904	chr12	ENSMUSG00000023075	chr4	ENSMUSG00000100240	chrY
ENSMUSG00000006356	chr12	ENSMUSG00000049225	chr4	ENSMUSG00000100467	chrY
ENSMUSG00000037787	chr12	ENSMUSG00000052137	chr4	ENSMUSG00000094746	chrY
ENSMUSG00000047227	chr12	ENSMUSG00000073758	chr4	ENSMUSG00000101528	chrY
ENSMUSG00000098530	chr12	ENSMUSG00000046671	chr4	ENSMUSG00000095793	chrY
ENSMUSG00000041716	chr12	ENSMUSG00000078716	chr4	ENSMUSG00000094647	chrY

ENSMUSG00000091931	chr12	ENSMUSG00000028718	chr4	ENSMUSG00000099740	chrY
ENSMUSG00000089875	chr13	ENSMUSG00000028437	chr4	ENSMUSG00000094399	chrY
ENSMUSG00000068184	chr13	ENSMUSG00000040859	chr4	ENSMUSG00000101928	chrY
ENSMUSG00000106200	chr13	ENSMUSG00000034401	chr4	ENSMUSG00000095578	chrY
ENSMUSG00000021519	chr13	ENSMUSG00000051435	chr4	ENSMUSG00000099840	chrY
ENSMUSG00000093930	chr13	ENSMUSG00000028572	chr4	ENSMUSG00000100032	chrY
ENSMUSG00000091383	chr13	ENSMUSG00000028427	chr4	ENSMUSG00000094821	chrY
ENSMUSG00000032846	chr13	ENSMUSG00000028436	chr4	ENSMUSG00000100972	chrY
ENSMUSG00000089827	chr13	ENSMUSG00000028845	chr4	ENSMUSG00000094616	chrY
ENSMUSG00000039219	chr13	ENSMUSG00000066324	chr4	ENSMUSG00000095979	chrY
ENSMUSG00000090497	chr13	ENSMUSG00000049488	chr4	ENSMUSG00000099782	chrY
ENSMUSG00000021694	chr13	ENSMUSG00000028443	chr4	ENSMUSG00000093895	chrY
ENSMUSG00000097292	chr13	ENSMUSG00000028294	chr4	ENSMUSG00000100608	chrY
ENSMUSG00000097276	chr13	ENSMUSG00000028706	chr4	ENSMUSG00000100856	chrY
ENSMUSG00000085393	chr13	ENSMUSG00000028409	chr4	ENSMUSG00000100645	chrY
ENSMUSG00000087202	chr13	ENSMUSG00000028896	chr4	ENSMUSG00000099792	chrY
ENSMUSG00000035762	chr13	ENSMUSG00000028878	chr4	ENSMUSG00000093993	chrY
ENSMUSG00000103373	chr13	ENSMUSG00000042608	chr4	ENSMUSG00000101155	chrY
ENSMUSG00000094870	chr13	ENSMUSG00000028965	chr4	ENSMUSG00000102739	chrY
ENSMUSG00000044792	chr13	ENSMUSG00000001089	chr4	ENSMUSG00000094325	chrY
ENSMUSG00000038462	chr13	ENSMUSG00000003032	chr4	ENSMUSG00000095634	chrY
ENSMUSG00000021576	chr13	ENSMUSG00000028760	chr4	ENSMUSG00000095725	chrY
ENSMUSG00000021669	chr13	ENSMUSG00000041459	chr4	ENSMUSG00000096275	chrY
ENSMUSG00000021546	chr13	ENSMUSG00000028277	chr4	ENSMUSG00000100203	chrY
ENSMUSG00000060093	chr13	ENSMUSG00000036241	chr4	ENSMUSG00000100353	chrY
ENSMUSG00000009470	chr13	ENSMUSG00000039768	chr4	ENSMUSG00000101010	chrY
ENSMUSG00000021668	chr13	ENSMUSG00000028438	chr4	ENSMUSG00000101675	chrY
ENSMUSG00000069265	chr13	ENSMUSG00000028551	chr4	ENSMUSG00000094623	chrY
ENSMUSG00000055137	chr13	ENSMUSG00000028383	chr4	ENSMUSG00000101417	chrY
ENSMUSG00000021302	chr13	ENSMUSG00000000411	chr4	ENSMUSG00000101993	chrY
ENSMUSG00000052293	chr13	ENSMUSG00000028952	chr4	ENSMUSG00000101110	chrY
ENSMUSG00000021635	chr13	ENSMUSG00000028389	chr4	ENSMUSG00000101541	chrY
ENSMUSG00000021476	chr13	ENSMUSG00000070999	chr4	ENSMUSG00000100171	chrY
ENSMUSG00000078941	chr13	ENSMUSG00000043633	chr4	ENSMUSG00000100520	chrY
ENSMUSG00000057069	chr13	ENSMUSG00000028248	chr4	ENSMUSG00000100677	chrY
ENSMUSG00000021518	chr13	ENSMUSG00000047613	chr4	ENSMUSG00000101221	chrY
ENSMUSG00000069237	chr13	ENSMUSG00000028813	chr4	ENSMUSG00000100582	chrY
ENSMUSG00000044444	chr13	ENSMUSG00000078554	chr4	ENSMUSG00000101022	chrY
ENSMUSG00000034928	chr13	ENSMUSG00000041399	chr4	ENSMUSG00000096272	chrY
ENSMUSG00000032745	chr13	ENSMUSG00000060268	chr4	ENSMUSG00000096698	chrY
ENSMUSG00000035367	chr13	ENSMUSG00000028637	chr4	ENSMUSG00000099791	chrY
ENSMUSG00000033799	chr13	ENSMUSG00000050608	chr4	ENSMUSG00000102294	chrY
ENSMUSG00000034686	chr13	ENSMUSG00000087611	chr5	ENSMUSG00000101518	chrY
ENSMUSG00000051054	chr13	ENSMUSG00000099144	chr5	ENSMUSG00000103112	chrY
ENSMUSG00000012429	chr13	ENSMUSG00000099391	chr5	ENSMUSG00000101131	chrY
ENSMUSG00000057649	chr13	ENSMUSG00000090750	chr5	ENSMUSG00000099653	chrY
ENSMUSG00000046957	chr13	ENSMUSG00000105052	chr5	ENSMUSG00000095192	chrY
ENSMUSG00000016982	chr13	ENSMUSG00000085720	chr5	ENSMUSG00000099834	chrY
ENSMUSG00000021374	chr13	ENSMUSG00000091422	chr5	ENSMUSG00000101310	chrY
ENSMUSG00000044934	chr13	ENSMUSG00000052154	chr5	ENSMUSG00000099915	chrY

ENSMUSG00000001542	chr13	ENSMUSG00000106594	chr5	ENSMUSG00000101748	chrY
ENSMUSG00000021690	chr13	ENSMUSG00000029384	chr5	ENSMUSG00000100964	chrY
ENSMUSG00000033102	chr13	ENSMUSG00000106026	chr5	ENSMUSG00000101670	chrY
ENSMUSG00000050799	chr13	ENSMUSG00000072612	chr5	ENSMUSG00000099505	chrY
ENSMUSG00000060081	chr13	ENSMUSG00000104789	chr5	ENSMUSG00000099371	chrY
ENSMUSG00000007836	chr13	ENSMUSG00000098230	chr5	ENSMUSG00000101490	chrY
ENSMUSG00000021758	chr13	ENSMUSG00000075569	chr5	ENSMUSG00000102079	chrY
ENSMUSG00000021326	chr13	ENSMUSG00000104873	chr5	ENSMUSG00000100072	chrY
ENSMUSG00000021701	chr13	ENSMUSG00000053121	chr5	ENSMUSG00000100473	chrY
ENSMUSG00000034751	chr13	ENSMUSG00000105978	chr5	ENSMUSG00000100343	chrY
ENSMUSG00000090641	chr13	ENSMUSG00000104811	chr5	ENSMUSG00000101228	chrY
ENSMUSG00000006720	chr13	ENSMUSG00000106506	chr5	ENSMUSG00000100644	chrY
ENSMUSG00000097333	chr13	ENSMUSG00000097561	chr5	ENSMUSG00000100669	chrY
ENSMUSG00000097565	chr13	ENSMUSG00000029386	chr5	ENSMUSG00000101005	chrY
ENSMUSG00000021534	chr13	ENSMUSG00000101856	chr5	ENSMUSG00000101990	chrY
ENSMUSG00000019132	chr13	ENSMUSG00000106229	chr5	ENSMUSG00000099645	chrY
ENSMUSG00000021569	chr13	ENSMUSG00000093355	chr5	ENSMUSG00000101837	chrY
ENSMUSG00000099315	chr14	ENSMUSG00000106004	chr5	ENSMUSG00000099593	chrY
ENSMUSG00000037536	chr14	ENSMUSG00000086368	chr5	ENSMUSG00000094064	chrY
ENSMUSG00000090772	chr14	ENSMUSG00000091273	chr5	ENSMUSG00000100581	chrY
ENSMUSG00000007589	chr14	ENSMUSG00000047635	chr5	ENSMUSG00000101688	chrY
ENSMUSG00000097997	chr14	ENSMUSG00000106261	chr5	ENSMUSG00000100973	chrY
ENSMUSG00000091595	chr14	ENSMUSG00000040407	chr5	ENSMUSG00000104085	chrY
ENSMUSG00000097775	chr14	ENSMUSG00000036555	chr5	ENSMUSG00000100116	chrY
ENSMUSG00000090104	chr14	ENSMUSG00000035364	chr5	ENSMUSG00000101593	chrY
ENSMUSG00000085829	chr14	ENSMUSG00000105881	chr5	ENSMUSG00000100442	chrY
ENSMUSG00000091116	chr14	ENSMUSG00000106393	chr5	ENSMUSG00000101379	chrY
ENSMUSG00000072145	chr14	ENSMUSG00000106164	chr5	ENSMUSG00000101472	chrY
ENSMUSG00000085615	chr14	ENSMUSG00000073147	chr5	ENSMUSG00000100142	chrY
ENSMUSG00000102611	chr14	ENSMUSG00000050050	chr5	ENSMUSG00000100226	chrY
ENSMUSG00000090396	chr14	ENSMUSG00000105157	chr5	ENSMUSG00000102230	chrY
ENSMUSG00000091788	chr14	ENSMUSG00000105618	chr5	ENSMUSG00000101408	chrY
ENSMUSG00000033712	chr14	ENSMUSG00000105479	chr5	ENSMUSG00000101844	chrY
ENSMUSG00000098626	chr14	ENSMUSG00000055424	chr5	ENSMUSG00000095626	chrY
ENSMUSG00000079379	chr14	ENSMUSG00000103864	chr5	ENSMUSG00000101072	chrY
ENSMUSG00000079407	chr14	ENSMUSG00000106148	chr5	ENSMUSG00000101752	chrY
ENSMUSG00000091325	chr14	ENSMUSG00000054909	chr5	ENSMUSG00000100464	chrY
ENSMUSG00000092499	chr14	ENSMUSG00000106034	chr5	ENSMUSG00000101787	chrY
ENSMUSG00000048628	chr14	ENSMUSG00000028995	chr5	ENSMUSG00000103909	chrY
ENSMUSG00000097172	chr14	ENSMUSG00000086047	chr5	ENSMUSG00000099489	chrY
ENSMUSG00000093949	chr14	ENSMUSG00000029600	chr5	ENSMUSG00000103005	chrY
ENSMUSG00000097199	chr14	ENSMUSG00000105299	chr5	ENSMUSG00000100421	chrY
ENSMUSG00000097822	chr14	ENSMUSG00000029414	chr5	ENSMUSG00000100501	chrY
ENSMUSG00000093167	chr14	ENSMUSG00000029314	chr5	ENSMUSG00000100449	chrY
ENSMUSG00000091849	chr14	ENSMUSG00000104845	chr5	ENSMUSG00000101623	chrY
ENSMUSG00000090788	chr14	ENSMUSG00000106652	chr5	ENSMUSG00000099700	chrY
ENSMUSG00000091605	chr14	ENSMUSG00000101678	chr5	ENSMUSG00000101259	chrY
ENSMUSG00000095280	chr14	ENSMUSG00000104982	chr5	ENSMUSG00000100994	chrY
ENSMUSG00000091995	chr14	ENSMUSG00000036323	chr5	ENSMUSG00000101980	chrY
ENSMUSG00000090796	chr14	ENSMUSG00000023104	chr5	ENSMUSG00000100148	chrY

ENSMUSG00000091281	chr14	ENSMUSG00000105688	chr5	ENSMUSG00000096532	chrY
ENSMUSG00000090462	chr14	ENSMUSG00000018143	chr5	ENSMUSG00000095179	chrY
ENSMUSG00000097278	chr14	ENSMUSG00000063015	chr5	ENSMUSG00000100275	chrY
ENSMUSG00000093954	chr14	ENSMUSG00000029422	chr5	ENSMUSG00000100355	chrY
ENSMUSG00000097589	chr14	ENSMUSG00000029166	chr5	ENSMUSG00000095433	chrY
ENSMUSG00000095528	chr14	ENSMUSG00000008348	chr5	ENSMUSG00000101998	chrY
ENSMUSG00000090635	chr14	ENSMUSG00000029405	chr5	ENSMUSG00000095049	chrY
ENSMUSG00000038925	chr14	ENSMUSG00000042594	chr5	ENSMUSG00000100390	chrY
ENSMUSG00000079272	chr14	ENSMUSG00000029168	chr5	ENSMUSG00000099527	chrY
ENSMUSG00000101797	chr14	ENSMUSG00000014956	chr5	ENSMUSG00000102103	chrY
ENSMUSG00000079266	chr14	ENSMUSG00000028938	chr5	ENSMUSG00000099975	chrY
ENSMUSG00000084951	chr14	ENSMUSG00000009013	chr5	ENSMUSG00000099709	chrY
ENSMUSG00000021840	chr14	ENSMUSG00000029580	chr5	ENSMUSG00000101147	chrY
ENSMUSG00000091627	chr14	ENSMUSG00000079562	chr5	ENSMUSG00000101474	chrY
ENSMUSG00000072676	chr14	ENSMUSG00000051674	chr5	ENSMUSG00000102081	chrY
ENSMUSG00000101261	chr14	ENSMUSG00000038582	chr5	ENSMUSG00000099992	chrY
ENSMUSG00000072731	chr14	ENSMUSG00000035851	chr5	ENSMUSG00000101821	chrY
ENSMUSG00000098609	chr14	ENSMUSG00000029196	chr5	ENSMUSG00000100978	chrY
ENSMUSG00000060373	chr14	ENSMUSG00000039782	chr5	ENSMUSG00000101340	chrY
ENSMUSG00000087652	chr14	ENSMUSG00000010721	chr5	ENSMUSG00000101747	chrY
ENSMUSG00000090798	chr14	ENSMUSG00000038095	chr5	ENSMUSG00000099944	chrY
ENSMUSG00000090861	chr14	ENSMUSG00000029165	chr5	ENSMUSG00000095345	chrY
ENSMUSG00000068758	chr14	ENSMUSG00000089984	chr5	ENSMUSG00000101709	chrY
ENSMUSG00000102151	chr14	ENSMUSG00000029186	chr5	ENSMUSG00000100661	chrY
ENSMUSG00000090742	chr14	ENSMUSG00000025858	chr5	ENSMUSG00000094232	chrY
ENSMUSG00000033730	chr14	ENSMUSG00000041740	chr5	ENSMUSG00000101719	chrY
ENSMUSG00000064128	chr14	ENSMUSG00000029439	chr5	ENSMUSG00000101161	chrY
ENSMUSG00000022174	chr14	ENSMUSG00000001687	chr5	ENSMUSG00000100719	chrY
ENSMUSG00000025544	chr14	ENSMUSG00000037795	chr5	ENSMUSG00000094529	chrY
ENSMUSG00000025277	chr14	ENSMUSG00000029427	chr5	ENSMUSG00000099684	chrY
ENSMUSG00000040997	chr14	ENSMUSG00000029152	chr5	ENSMUSG00000099972	chrY
ENSMUSG00000021748	chr14	ENSMUSG00000066278	chr5	ENSMUSG00000100643	chrY
ENSMUSG00000021273	chr14	ENSMUSG00000029147	chr5	ENSMUSG00000095553	chrY
ENSMUSG00000021877	chr14	ENSMUSG00000029148	chr5	ENSMUSG00000099460	chrY
ENSMUSG00000021786	chr14	ENSMUSG00000040473	chr5	ENSMUSG00000100256	chrY
ENSMUSG00000021993	chr14	ENSMUSG00000037979	chr5	ENSMUSG00000100958	chrY
ENSMUSG00000022052	chr14	ENSMUSG00000038126	chr5	ENSMUSG00000101478	chrY
ENSMUSG00000060126	chr14	ENSMUSG00000029536	chr5	ENSMUSG00000101149	chrY
ENSMUSG00000021785	chr14	ENSMUSG00000029469	chr5	ENSMUSG00000101128	chrY
ENSMUSG00000002324	chr14	ENSMUSG00000050677	chr5	ENSMUSG00000104070	chrY
ENSMUSG00000022013	chr14	ENSMUSG00000038525	chr5	ENSMUSG00000099524	chrY
ENSMUSG00000035161	chr14	ENSMUSG00000029248	chr5	ENSMUSG00000102906	chrY
ENSMUSG00000022035	chr14	ENSMUSG00000029125	chr5	ENSMUSG00000101105	chrY
ENSMUSG00000021917	chr14	ENSMUSG00000041930	chr5	ENSMUSG00000104404	chrY
ENSMUSG00000022009	chr14	ENSMUSG00000029004	chr5	ENSMUSG00000101788	chrY
ENSMUSG00000045107	chr14	ENSMUSG00000095789	chr5	ENSMUSG00000101017	chrY
ENSMUSG00000021916	chr14	ENSMUSG00000028953	chr5	ENSMUSG00000100519	chrY
ENSMUSG00000034327	chr14	ENSMUSG00000038722	chr5	ENSMUSG00000100325	chrY
ENSMUSG00000037697	chr14	ENSMUSG00000005103	chr5	ENSMUSG00000099755	chrY
ENSMUSG00000052584	chr14	ENSMUSG00000000148	chr5	ENSMUSG00000101954	chrY

ENSMUSG00000022008	chr14	ENSMUSG00000048439	chr5	ENSMUSG00000100179	chrY
ENSMUSG00000021890	chr14	ENSMUSG00000079065	chr5	ENSMUSG00000095968	chrY
ENSMUSG00000042682	chr14	ENSMUSG00000029155	chr5	ENSMUSG00000100244	chrY
ENSMUSG00000048922	chr14	ENSMUSG00000066900	chr5	ENSMUSG00000099425	chrY
ENSMUSG00000041408	chr14	ENSMUSG00000029623	chr5	ENSMUSG00000100076	chrY
ENSMUSG00000042888	chr14	ENSMUSG00000036087	chr5	ENSMUSG00000100509	chrY
ENSMUSG00000021986	chr14	ENSMUSG00000002748	chr5	ENSMUSG00000096332	chrY
ENSMUSG00000045211	chr14	ENSMUSG00000029110	chr5	ENSMUSG00000101369	chrY
ENSMUSG00000021891	chr14	ENSMUSG00000029477	chr5	ENSMUSG00000101489	chrY
ENSMUSG00000021969	chr14	ENSMUSG00000096344	chr5	ENSMUSG00000104024	chrY

Table S5. Number of genes (with coding potential) identified using Ensembl protein domain search

	Mouse*	Rat	Steppe mouse	Chinese hamster PICR	Mongolian gerbil	Northern American deer mouse	Ryukyu mouse
**Speer/*Takusan* gene family (chr5 and 14)**	219	68	17	11	4	4	3
***Sycp3* gene family (XY chr)**	116	7	4	9	3	2	3
***Spindlin* gene family (XY chr)**	64	38	12	8	4	4	3
***Btbd35f/Mgclh* (X chr)**	30	1	1	1	0	1	0
***H2al* gene family (X chr)**	17	1	4	2	3	2	3
***Samt* gene family (X chr)**	8	9	6	11	4	13	1
***Smok2a* (var autosomes - including chr5,17)**	23	15	8	24	6	19	7

**Mus musculus*

Table S6. Summary of the experimental procedure used for TBL1XR1 ChIP Seq analyses performed in WT and *Sly* -KD spermatids

	**TBL1XR1**
technique to collect round spermatids (RS)	elutriation
Nb of cells	5 millions of WT RS and 5 millions of Sly-KD RS
purity (% of RS)	~90% for WT and KD
PFA crosslink	cross link was performed <u>after</u> freezing
quantity of antibody	~ 3ul of antibody per 5 millions of cells

	**ChIP TBL1XR1 on WT RS**	**ChIP TBL1XR1 on Sly-KD RS**	**WT_KD Input**
Total number of reads	47 003 281	51 104 049	51 312 862
Total number of alignments	18 032 922	43 088 807	50 672 079
Final number of tags (no duplicate reads)	8 816 317	28 259 310	42 881 615
Redundant rate	0,51	0,34	0,15
	**MACS 1.4.2 peak calling**		
	# band width = 300; # model fold = 10,32; # pvalue cutoff = 1.00e-05		
Nb of Peaks (mapped)	1 812	164	
Number of genes at +/- 1Kb of TSS (Ensembl80)	318	19	

Supplementary Table 7. List of primers designed for the study.

Primers used for RTqPCR

Gene	Primer sequence	Location	Annealing T°C
Speer4RT *(Speer4c)*	TGGAGGCCCAAGAGAACAAG<br>TGATGGCCCAATCAGCCTGT	gene	60°C
TakusanRT (Gm21162)	AGAGACGAACTTCAGGGAATCC<br>TGACTTTGTGCTGGATGAGGTT	gene	60°C
Prr13RT	AGCTCATAAGAAGTCGCACAAG<br>TGCGTTCAGTCAGAGTCACT	gene	60°C

Primers used for ChIPqPCR

Name	Primer sequence	Location	Annealing T°C
SlxChIP	CATTCTTCCTACGCCACTCC<br>CCTTAGGGTCCAACTTGTCG	TSS (420 bp downstream TSS)	55°C
H2afb3ChIP	CAGAGTGCGATGTCACAGGA<br>GTCCGGTACAAAGGGAGACA	TSS	55°C
Speer4ChIP *(Speer4a)*	TAGCATCCTTCCTCCCCTCT<br>TCCGTGATGTCACCAGTGTT	TSS	55°C
TakusanChIP *(Gm10376)*	AAGCTGGCAAGCAGGTAAGC<br>GGAATCCCTCACAGGCAACT	TSS	60°C
Dot1lChIP	GGGATTCCTTGCCTTTCGTG<br>CCCCTGACTTCCTAGGGTTCT	TSS (541 bp downstream TSS)	55°C
Prr13ChIP	GCAACGGTTTCCTCCTTGTT<br>TCAGTCTGGGTTCGCACTTC	TSS	55°C
Zfy2ChIP	AGCTTGAATCTTTTGCTGGAGG<br>CCAGGGTGGTCAGAACTATGG	TSS	55°C
NC	TGGCATTGTGGGCTAGATTT<br>TGGAGATAAGATATGCGTCAAG	intragenic (170kb downstream TSS)	55°C
NC2	GCTGAATGAGCCAGAGTGGTA<br>AGCCTCCCACGTCTTAATCC	intragenic (40kb downstream TSS)	55°C
